# Controlling aggregation-induced emission by supramolecular interactions and colloidal stability in ionic emitters for light-emitting electrochemical cells†

**DOI:** 10.1039/d3sc05941c

**Published:** 2024-01-25

**Authors:** Alba Sanz-Velasco, Olivia Amargós-Reyes, Aya Kähäri, Sophia Lipinski, Luca M. Cavinato, Rubén D. Costa, Mauri A. Kostiainen, Eduardo Anaya-Plaza

**Affiliations:** a Department of Bioproducts and Biosystems, Aalto University Kemistintie 1 02150 Espoo Finland eduardo.anaya@aalto.fi; b Technical University of Munich, Campus Straubing for Biotechnology and Sustainability Schulgasse 22 94315 Straubing Germany ruben.costa@tum.de

## Abstract

Chromophores face applicability limitations due to their natural tendency to aggregate, with a subsequent deactivation of their emission features. Hence, there has been a fast development of aggregation induced emission (AIE) emitters, in which non-radiative motional deactivation is inhibited. However, a fine control of their colloidal properties governing the emitting performance is fundamental for their application in thin film optoelectronics. In addition, ion-based lighting devices, such as light emitting electrochemical cells (LECs), requires the design of ionic AIE emitters, whose structure allows (i) an easy ion polarizability to assist charge injection and (ii) a reversible electrochemical behavior. To date, these fundamental questions have not been addressed. Herein, the hydrophilic/hydrophobic balance of a family of cationic tetraphenyl ethene (TPE) derivatives is finely tuned by chemical design. The hydrophilic yet repulsive effect of pyridinium-based cationic moieties is balanced with hydrophobic variables (long alkyl chains or counterion chemistry), leading to (i) a control between monomeric/aggregate state ruling photoluminescence, (ii) redox behavior, and (iii) enhanced ion conductivity in thin films. This resulted in a LEC enhancement with the first ionic AIE emitters, reaching values of 0.19 lm W^−1^ at *ca.* 50 cd m^−2^. Overall, this design rule will be key to advance ionic active species for optoelectronics.

## Abstract 2

In this work, we study the interplay between hydrophilicity/hydrophobicity in cationic aggregation induced emitters, and their application in light emitting electrochemical cells.

## Graphical abstract

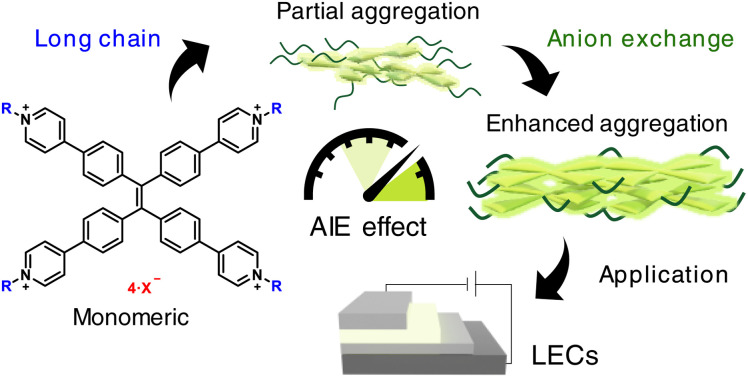

## Introduction

Organic fluorophores have drawn intensive attention during last decades for their practical applications, named medical imaging, light emitting diodes (LEDs), or chemical sensors.^1–4^ However, their use finds limitations due to the partial or complete emission quenching derived from aggregation, leading to the ‘aggregation caused quenching’ (ACQ). This is consequence of their usually large aromatic structures that lead to π–π stacking, and subsequently deactivates the photoexcited state(s) through non-radiative pathways.^5^ This phenomenon typically occurs at high concentrations, aqueous media given the high hydrophobicity of these derivatives, or the condensed/solid state.^5,6^ Thus, aggregation limits their applicability as high payload imaging agents, phototherapeutic agents, or in lighting devices (*e.g.*, OLEDs or solar cells), respectively. While chemical design of non-aggregating dyes,^7,8^ or directed aggregation^9,10^ has been pursued with success, a radically different approach has been explored to turn dye aggregation into a desirable feature.

Aggregation induced emission (AIE) is a photophysical phenomenon coined in 2001, in which aggregate formation enhances the light emitting process.^11,12^ AIE emitters (AIEgens) in their monomeric state suffer from emission quenching caused by intramolecular vibrations and rotations in *e.g.*, organic solvents, leading to emission quenching. In contrast, rigidification caused by aggregation blocks intramolecular motion, recovering their emitting properties.^13–15^ Consequently, AIEgens exhibit emitting properties in aggregate state. Such exceptional characteristics have made them suitable for fields where traditional photosensitizers performance is hindered.^16–18^

Despite the plethora of AIEgen families, ionic AIE structures are understudied, likely given the *a priori* counterproductive properties: charged molecules will present enhanced aqueous solubility that, together with the coulombic repulsion, hinder self-assembly and, therefore, the AIE properties.^19^ For instance, ionic AIE probes have been used as protein labelling agents, with the AIE properties arising upon biomolecular recognition.^20^ However, a deep understanding of factors influencing aggregation and monomeric states of ionic AIEgens in aqueous media and solid-state is crucial for controlling system forces and then, expanding applications (*e.g.* ultra-sensitive mechanochromic luminescent materials).^21^ In this context, ionic AIEgens present a unique potential for ionic-based optoelectronics, such as light-emitting electrochemical cells (LECs).^22^ They are considered the simplest thin film lighting device, in which the presence of ions in the active layer allows (i) an efficient charge injection from air stable electrodes, and (ii) a controlled electrochemical doping of the emitter forming a stable p-i-n junction, in which exciton radiative recombination occurs. Recently, Edman's group showed for the first time that neutral AIE emitters with a rigid and emissive structure will be compatible with ionic electrolytes, realizing better devices than analogous emitters with typical ACQ.^23^ However, the use of ionic AIE emitters as well as the design principles to simultaneously merge desired photophysical, electrochemical, and ion transport for LECs remains unanswered.

Herein we present a new versatile platform of positively charged structures derived from tetra-(4-pyridylphenyl)-ethylene 1,1,2,2-tetrakis(4-(pyridin-4-yl)phenyl)ethene (TPPE) that exhibits AIE effect upon aggregation.^24–28^ They present a propeller-like structure formed by four phenyl groups moving freely in the monomeric state. The hydrophilic/hydrophobic balance governing the AIE effect is finely tuned by the length of alkyl chain and the counterion: on one hand, the hydrophilicity is introduced by the positively charged pyridinium moieties that increase the solubility in aqueous media. On the other hand, the alkyl chain and the counterion nature modulate the hydrophobicity, and thus, overcoming electrostatic repulsions and promoting self-aggregation. We demonstrate the impact of the mentioned forces on the optical properties in organic solvent (monomeric) and aqueous media (monomeric or aggregate) at different concentrations in the series of halide TPPE-alkyl salts and hexafluorophosphate (PF_6_^−^) TPPE-alkyl salts. The length of the alkyl chains plays a key role on self-aggregation for TPPE-alkyl salts: C_12_ chains are needed in order to turn on the AIE effect. PF_6_TPPE-alkyl salts, however, exhibit an enhanced AIE at lower concentrations and with shorter alkyl chains as well as a more reversible electrochemical behavior. Furthermore, we demonstrate the impact of the molecular structure in the electrochemical behavior and ion conductivity in thin films. This series was further applied to LECs nicely showing that (i) the emission features of the films enhance with the AIE behavior, (ii) the ion-assisted mechanism is fully operative and (iii) a linear trend to increase device figures of merit upon increasing the alkyl chain. This resulted in the first LECs with ionic AIE emitters, exhibiting a yellow emission associated to *ca.* 50 cd m^−2^ and 0.19 lm W^−1^, which is in the range of small molecules LECs.^29^

## Results and discussion

### Aggregation induced emission of cationic TPPE derivatives

#### Synthesis of the halide TPPE-alkyl salts

**Scheme 1:**
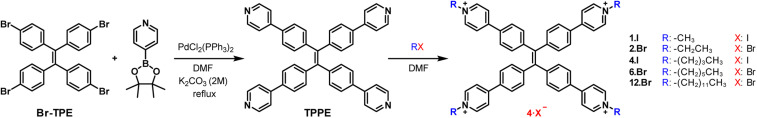
Synthesis of the family of TPPE-alkyl salts *via* Suzuki cross-coupling reaction and alkylation.

Synthesis of the TPPE core was carried out through a Pd-catalyzed Suzuki coupling of commercially available 1,1,2,2-tetrakis(4-bromophenyl)-ethylene (Br-TPE) (see ESI Fig. S1†), followed by the alkylation of the pyridine moiety with the corresponding alkyl halide (Scheme 1).^28^ The selected library displays equal number and nature of pyridinium-based cationic charges, ranging from methyl (1.I), ethyl (2.Br), *n*-butyl (4.I), hexyl (6.Br) to dodecyl (12.Br) salts (Scheme 1). The resulting compounds were thoroughly characterized by means of NMR spectroscopy and mass spectrometry (see ESI, Fig. S2 to S31†).

#### Photophysical properties of the halide TPPE-alkyl salts in solution

**Fig. 1:**
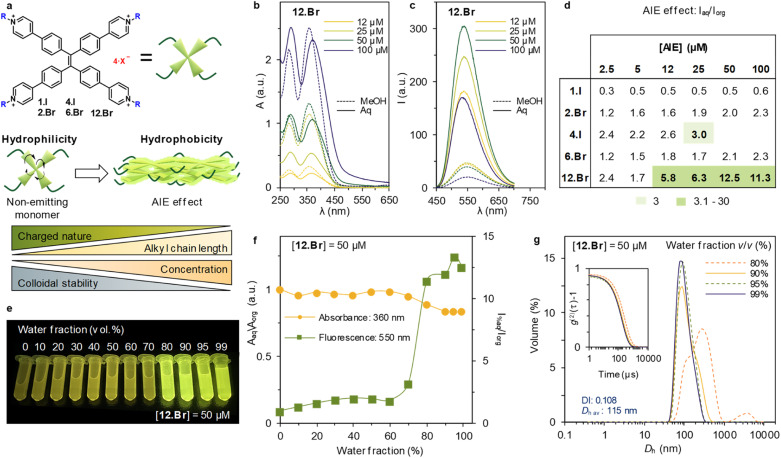
(a) Schematic representation of the TPE derivatives, as well as the key design features and supramolecular driving forces leading to monomeric or aggregating state in aqueous media. (b) UV-vis absorbance spectra of 12.Br at different concentrations (full and dashed lines for sample in aqueous and organic media, respectively). (c) Emission spectra of 12.Br at different concentrations (full and dashed lines for sample in aqueous and organic media, respectively) upon excitation at 360 nm. (d) Ratio *I*_aq_/*I*_org_ at maximum of emission (550 nm) at varying concentrations of the series. In light green and green are marked results with a ratio of 3 and 3.1–30, respectively. (e) Image of 12.Br ([12.Br] = 50 μM) titration at different water fraction content, showing increased emission above 80% of water. (f) Titration of [12.Br] = 50 μM at varying water percentage. Absorbance (orange line) at 360 nm and emission (green line) at 550 nm upon excitation at 360 nm. A sharp increase can be found above 80% water/MeOH mixture. (g) Volume-averaged particle size distribution of [12.Br] = 50 μM measured by DLS, showing high dispersity at 80% and size stabilization at 90% and above. No data was obtained for solutions below 80% water, indicating lack of aggregates. Inset: second order normalized autocorrelation function of the selected samples. Dispersity index (DI) and volume-averaged hydrodynamic diameter (*D*_h_ av.) correspond to average obtained after three repetitions of 12.Br at 99% water/MeOH mixture.

The synthesized family presents a fine-tuned hydrophilicity/hydrophobicity balance between the positively charged pyridinium moieties and the increasing chain length, respectively. On one hand, positive charges render aqueous solubility and coulombic repulsion render monomeric derivatives. On the other hand, long alkyl chains render aggregation in aqueous media, counterbalancing the electrostatic repulsion (Fig. 1a). The subtle interplay of forces has direct impact on the optical properties, in particular between the luminescence in organic solvent, *i.e.* methanol, and aqueous media (containing a 1% v/v fraction of the organic solvent). The first renders the AIEgen in monomeric state, while the aqueous dispersion will either exhibit monomeric or aggregated (*i.e.*, emitting) state, depending on the overall hydrophilicity/hydrophobicity balance.

First, the absorbance spectra of the halide TPPE-alkyl salts are recorded at different concentrations, both in organic and aqueous media (*A*_org_ and *A*_aq_, respectively), meaning aqueous media 99% of water content in the sample (ESI Fig. S62†). The synthetized family of compounds shows consistent absorption maxima, centered at *ca.* 280 and 360 nm (Fig. 1b) with minor changes between organic and aqueous solvent. However, 12.Br shows a minor shift in aqueous media, that becomes more evident with the increase in concentration (Fig. 1b). Further analysis of the *A*_360_ dependence with the concentration (see S62†) shows linearity, in good agreement with the Lambert–Beer law, for the whole series.

Subsequently, the emission spectra upon excitation at 360 nm is recorded in organic and aqueous media (*I*_org_ and *I*_aq_, respectively). The photoluminescence intensity is shown at different concentrations for 12.Br in Fig. 1c and S62† for the rest of the series. The general trend shows enhanced emission in aqueous media (*I*_aq_ > *I*_org_), which is in agreement with the AIE effect. In order to determine the extent of this increase through the series, Fig. 1d shows the ratio between *I*_aq_ and *I*_org_ at different concentrations. 4.I shows limited solubility in organic solvents, and therefore cannot be dissolved in a concentration higher than 2.5 mM, limiting the concentration to 25 μM.

Furthermore, we consider a minimum threshold of three-fold increase (marked as light green on the table) to be a relevant AIE effect. This threshold is a desirable noise-to-signal ratio in *e.g.*, microscopy. The table at Fig. 1d highlights the interplay between the alkyl side chains and the electrostatic repulsions: only 12.Br above 12 μM shows an emission increase of 5.8, whereas 6.Br at the same concentration only displays an increase of 1.8 times. This exemplifies the role of alkyl chains in the formation of emitting aggregates. Therefore, the optimized conditions of the series are obtained for [12.Br] = 50 μM, and thus, a thorough study at different water percentages was carried out at this concentration under these conditions.

First, the emitting properties at decreasing organic fraction solvents are recorded. The AIE effect as described above is turned on when the sample contains over 80% of water content (Fig. 1e). Notwithstanding, once aggregates are formed, the emission obtained for the sample at 99% of water is almost 13 times higher compared to in organic solvent (Fig. 1f). The relationship between aggregation and emitting properties is further confirmed by monitoring the size distribution measured by DLS at different water fractions. It was observed that aggregates start forming at 80% of water content in the sample (see ESI Fig. S66†). After, structures reduce the diameter from 185 nm (80% water, DI: 0.178) to 115 nm (99% water, DI: 0.108).

### Counterion chemistry as aggregation inducing emission parameter

#### Synthesis of the PF_6_TPPE-alkyl salts

**Fig. 2:**
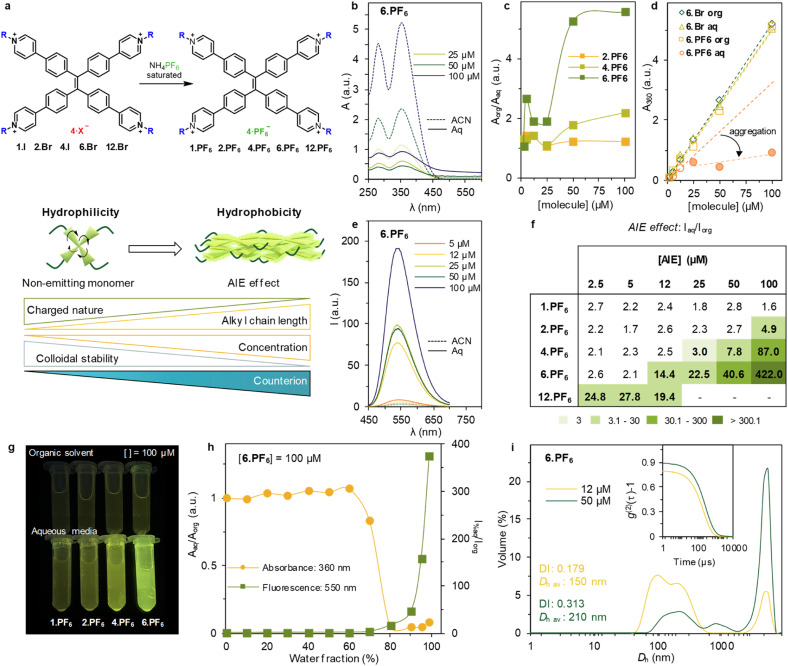
(a) Synthetic path for ion exchange of the series. Scheme of the effect of the counterion modification in supramolecular driving forces that lead to monomeric or aggregated state in aqueous media. (b) UV-vis absorbance spectra of 6.PF_6_ at different concentrations (full and dashed lines for sample in aqueous and organic media, respectively). (c) Ratio *A*_org_/*A*_aq_ at 360 nm at different concentrations for 2.PF_6_, 4.PF_6_ and 6.PF_6_, showing a sharp increase due to aggregation for 6.PF_6_. (d) *A*_360_ at different concentrations for 6.Br in organic solvent (blue rhombus), 6.Br in aqueous media (green triangles), 6.PF_6_ in organic solvent (yellow squares) and 6.PF_6_ in aqueous media (red circles). The trendlines are marked with the same colour. In the case of 6.PF_6_, there is a trendline based on the three first concentrations and a second trendline (light red) corresponding to the results at 25, 50 and 100 μM highlighting the self-aggregation at these concentrations. (e) Emission spectra of 6.PF_6_ at different concentrations (full and dashed lines for samples in aqueous and organic media, respectively) upon excitation at 360 nm. (f) Ratio *I*_aq_/*I*_org_ at maximum of emission (550 nm) at varying concentrations of the PF_6_-family compounds. Different green intensities have been used to emphasize the AIE effect. At [12.PF_6_] > 12 μM sample not soluble in water. (g) Images of 1.PF_6_, 2.PF_6_, 4.PF_6_ and 6.PF_6_ (left to right) at 100 μM in organic solvent (upper row) and aqueous media (lower row). (h) Titration of [6.PF_6_] = 50 μM varying water percentage. Absorbance results at 360 nm and fluorescence results at 550 nm by excitation at 360 nm. (i) Volume-averaged particle size distribution of [6.PF_6_] = 12 μM (yellow) and 50 μM (green) in aqueous media measured by DLS. Inset: second order normalized autocorrelation function of the selected samples. DI and *D*_h_ av. correspond to average obtained after three repetitions at 99% water/MeOH mixture.

Although the selected family of positively-charged AIEs has been achieved by side-chain chemistry, the desired AIE effect is only achieved upon conjugation with long (C_12_) chains. Therefore, to further promote the hydrophobicity, the counterion exchange for more hydrophobic derivatives was explored. Hexafluorophosphate anion (PF_6_^−^) was chosen due to its hydrophobic nature and suitability towards LECs – *vide infra*.^30^ The ion exchange was carried out by dissolving the halide TPPE-alkyl salt in water or methanol and combining it with a saturated solution of ammonium hexafluorophosphate (NH_4_PF_6_), in the same solvent, obtain the PF_6_-containing derivatives (*i.e.*, 1.PF_6_, 2.PF_6_, 4.PF_6_, 6.PF_6_ and 12.PF_6_, Fig. 2a). The final structures obtained were thoroughly characterized by spectroscopy (see ESI Fig. S32 to S61†).

#### Photophysical properties of the PF_6_TPPE-alkyl salts in solution

The PF_6_-family exhibits an updated hydrophilicity/hydrophobicity balance, adding the counterion chemistry to the balance between the positively charged pyridinium moieties and the side chain length. This hinders the electrostatic repulsion more efficiently than solely sidechain chemistry and promotes self-aggregation in aqueous media (Fig. 2a). The absorbance spectra of PF_6_TPPE-alkyl salts are recorded at different concentrations for both, organic and aqueous media (Fig. S64†). The PF_6_-family shows absorption maxima consistent with the halide derivatives, centred at *ca.* 280 and 360 nm (Fig. 2b). However, *A*_aq_ displays a remarkable decrease compared to *A*_org_ that becomes more evident with the increase in the concentration and the length of the alkyl chain and exemplified by 6.PF_6_ (Fig. 2c and S64†). The ratio between *A*_org_/*A*_aq_ at 360 nm shows a sharp increase above 25 μM, as consequence of the heavy aggregation in aqueous media, and is accentuated with the increasing side-chain length. The counterion effect is more evident when comparing the halide counterpart. Plotting the *A*_aq,_ at 360 nm for 6.Br and 6.PF_6_ in both organic and aqueous media (Fig. 2d) shows an acute deviation of 6.PF_6_ from the Lambert–Beer law (Fig. 2d, red circles) when concentration reaches 25 μM, a clear indication of saturation of the system, loss of colloidal stability, and eventual precipitation. In addition, the emission spectra upon excitation at 360 nm is recorded for the PF_6_-family in organic and aqueous media (see Fig. S64†). The emission intensity of 6.PF_6_ at different concentrations is shown in Fig. 2e and S64,† exhibiting a major emission enhancement in aqueous media when compared with the one in organic solvent. This is in line with the aggregation shown in the absorbance. To normalize the emission across the concentrations, the ratio between *I*_aq_ and *I*_org_ at given conditions is plotted again in a table (Fig. 2f). The table summarizes the subtle interplay that alkyl side chains, counterion, concentration, and electrostatic repulsions play in this system. The PF_6_-family shows an AIE effect with shorter side chains and lower concentrations (Fig. 2f*vs.*1d). Moreover, the hydrophobicity introduced by C_12_ chains in combination with a concentration increase is critical: [12.PF_6_] = 5 μM exhibits an emission increment of 27.8, whereas [12.PF_6_] = 12 μM is reduced to 19.4. A plausible explanation is the oversaturation of the system that leads to visible flocculation and particle precipitation, hindering the accurate measurement in solution. This effect is observed with higher concentrations, thus reaching the limit of colloidal stability, and leading to quantitative precipitation. This poor colloidal behaviour limits the scope of further studies, and 12.PF_6_ is not further studied. Fig. 2g shows the difference in emission between organic solvent and aqueous media, and the effect of the side chain length at 100 μM for the series. A more detailed understanding of the optimized conditions of the series (*i.e.* [6.PF_6_] = 100 μM) is obtained by stepwise increase of the water content (Fig. 2h). First, the absorbance spectrum and the emitting properties are recorded under these conditions (Fig. 2h). The heavy decrease in *A*_360_ at 80% of water, consistent with the increase of emission at 550 nm marks the conditions for aggregate formation. The size of this aggregates is further studied by DLS measurements (Fig. S66†). At [6.PF_6_] = 100 μM the autocorrelation is not suitable for fitting, most likely due to *i.e.* precipitation. Therefore, further studies were carried out at 12 and 50 μM (Fig. 2i). Under these conditions, aggregates at 99% of water content in the sample shows sizes of 149.8 and 210.1 nm, respectively, although higher dispersities. This points towards a lower colloidal stability in water of the PF_6_-family, when compared with their halide counterparts.

#### Electrochemical characterization of the PF_6_TPPE-alkyl salts in solution

Altogether, the PF_6_ family going from 1.PF_6_ to 6.PF_6_ derivatives show superior AIE properties, promising for their use in lighting devices.^31,32^ Thus, we turned to investigate the effect of the substitution pattern on the electrochemical behaviour using square wave voltammetry (SWV) in MeCN (Table 1 and Fig. S67†). Though two well-defined sequential reductions at 1.36 V and 1.60 V are noted for all compounds, 1.PF_6_ and 2.PF_6_ showed a partial reversibility, while 4.PF_6_ and 6.PF_6_ are almost quasi reversible. This goes in line with the anodic process, in which multiple oxidation waves in the range of 1.0–2.5 V turned to be more defined and reversible with the increase of the alkyl chain length. Thus, it is expected that charge injection and transport will be critical for the device performance, while the increase of the alkyl chain provides the most promising redox behaviour (4.PF_6_ and 6.PF_6_) for the implementation into LECs – *vide infra*.

**Table 1:** Electrochemical features *vs.* Fc/Fc^+^ of 1.PF_6_, 2.PF_6_, 4.PF_6_ and 6.PF_6_ in MeCN/NBu_4_PF_6_ (0.1 M)

Square wave voltammetry			
	*E*_ox_ (V)/*E*_HOMO_ (eV)	*E*_red_ (V)/*E*_LUMO_ (eV)	Δ*E* (HOMO–LUMO) (eV)/(nm)
1.PF_6_	1.3, 1.6/−6.3^a^	−1.4, −1.6/−3.8^a^	2.5/496
2.PF_6_	1.1, 1.3, 1.5/−6.3^a^	−1.4, −1.6/−3.8^a^	2.5/496
4.PF_6_	1.1, 1.5, 1.9/−6.2^a^	−1.4, −1.6/−3.8^a^	2.6/477
6.PF_6_	1.1, 1.3, 1.5/−6.2^a^	−1.4, −1.6/−3.8^a^	2.6/477

a Calculated from the onset of the oxidation–reduction potential.^33^

#### Photophysical studies of the PF_6_TPPE-alkyl salts in thin films

**Fig. 3:**
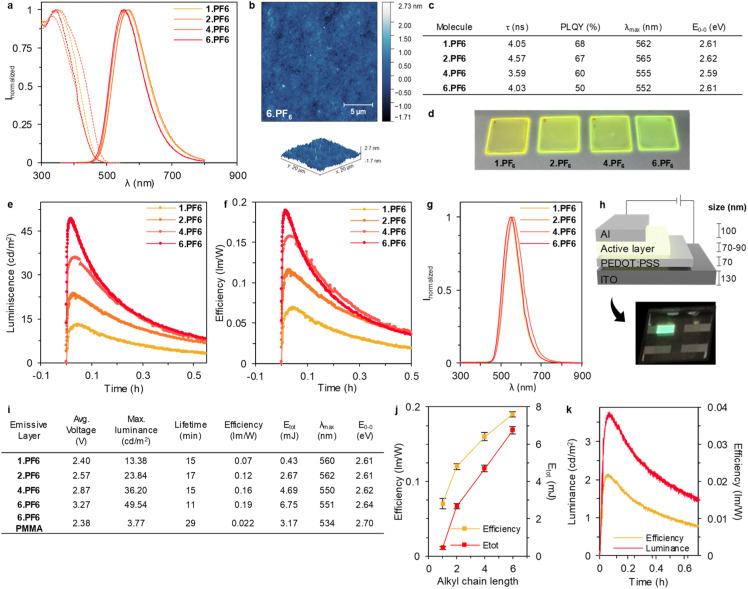
(a) AFM topography of the 6.PF_6_ film dried under vacuum treatment, and its profile (bottom). (b) Excitation and emission spectra (dashed and full lines, respectively) in thin films. (c) Photoluminescence features of the films. (d) Pictures under UV illumination (305 nm) of the thin films deposited on quartz. (e) Luminance and (f) efficiency of the LECs. (g) Normalized electroluminescence spectra of the devices. (h) Schematic of the device's architecture, in addition to an optical image of a device's pixel *in operando*. (i) Devices performance at pulsed current density driving of 100 mA cm^−2^. LECs prepared with 6.PF_6_ mixed with PMMA (6.PF_6_-PMMA) to disrupt aggregation. (j) Efficiency (yellow) and total emitted energy (red) in relationship with alkyl chains length. (k) Luminance (red) and efficiency (yellow) of LECs made with 6.PF_6_-PMMA.

Thin films (∼70–90 nm) were prepared on quartz *via* spin-coating using MeCN solutions (see the Experimental section for details) following a vacuum (3 mbar for 50 min) drying process and a subsequent standard temperature treatment at 90 °C for 30 min under inert atmosphere, since the working temperature of LECs spans from 35–75 °C depending on the driving conditions.^34,35^ Atomic force microscopy (AFM) images (Fig. 3a and S69†) of the thin films confirmed a homogeneous morphology with neither apparent phase separation nor large aggregates and small hillocks formations related to the slow evaporation of MeCN upon film forming. Overall, the root mean square (RMS) roughness value for all the films was around 300 pm. The photoluminescence features (Fig. 3b and c) show broad emission band centred at 562 and 565 nm for 1.PF_6_ and 2.PF_6_, further blue-shifted to 555 nm and 552 nm for 4.PF_6_ and 6.PF_6_, suggesting a different type of aggregation and/or molecular conformation induced by the length of the alkyl chain as expected from the solution studies. This is nicely reflected in the photoluminescence quantum yields (PLQYs) that evolve from 68% and 67% (1.PF_6_ and 2.PF_6_) to 60% and 50% (4.PF_6_ and 6.PF_6_), while the excited state device lifetime values (*τ*) are around 4 ns, as expected for a fluorescence emission process. Thus, the benefits of the AIE 4.PF_6_ and 6.PF_6_ also stand out in terms of thin-film photoluminescence behaviour for LEC application.

#### Light-emitting electrochemical cells (LEC) with ionic AIE emitters

LECs were fabricated with a double-layered architecture ITO/PEDOT:PSS (70 nm)/active layer (∼70 nm)/Al (100 nm) and analysed by (i) static electrochemical impedance spectroscopy (EIS) to determine ion mobility features in the active layer, and (ii) monitoring the luminance, colour (*x*/*y* CIE colour coordinates), and electrical behaviour over time at pulsed current density of 100 mA cm^−2^. EIS technique is very useful to determine electronic and ion polarization effects that rule the formation of electrical double layers (EDLs) allowing injection from the air-stable electrodes (dielectric constant or *ε*) and growing of the electrochemical doped regions (ion conductivity or *σ*) that define the stability of the p-i-n junction (Fig. S70†).^36–40^ In short, we applied a DC voltage at 0 V to an AC signal of 10 mV amplitude and at frequencies from 10^6^ to 10 Hz. The Nyquist plots were analysed using a single resistor/capacitor equivalent circuit (Fig. S70†). A direct comparison between 1.PF_6_ and 2.PF_6_ (low AIE feature) and 4.PF_6_ and 6.PF_6_ (strong AIE feature) films, shows that the lock-structure of AIE emitters slightly impact the *ε* values that hold at *ca.* 8 (Fig. 3c). Likewise, all the devices showed a similar *σ* value in the regime of 5–9 × 10^−7^ S m^−1^. Thus, 6.PF_6_ systems can also build up EDLs and assist the growth of the electrochemical doped regions, regardless of the alkyl chains length compared to 1.PF_6_. Overall, these findings are in line with the recent contribution of Edman,^23^ corroborating that the lock morphology of AIE emitters allows ion polarization regardless of their neutral or ionic nature.

Next, we studied the electroluminescent behaviour of the LECs at pulsed current density of 100 mA cm^−2^. As expected, all the devices exhibited a reduction of the applied voltage along with an increase of luminance response (Fig. 3e, f and S70†). Indeed, an instantaneous brightness response associated to a broad emission spectrum centred at 550 nm and 560 nm for 1.PF_6_/2.PF_6_ and 4.PF_6_/6.PF_6_, respectively. Thus, photo-/electroluminescence band shapes and the maximum emission wavelength remains without notable variation for each molecule, indicating that the same excited state is involved regardless of the photo/electrical excitation process over time. (*E*_0–0_ and *λ*_max_ in Fig. 3c (PL) and Fig. 3i (EL). While the emission band is stable for 4.PF_6_/6.PF_6_ devices, those of 1.PF_6_/2.PF_6_ slightly red-shifts over time due to the increase of the device temperature to *ca.* 45 °C – *vide supra*. Overall, the device chromaticity corresponds to a greenish emission as corroborated by the *x*/*y* CIE colour coordinates of 0.40–4/0.51–4. More interesting, the luminous efficiency and device brightness gradually increases with the length of the alkyl chain, reaching values of 0.07, 0.12, 0.16, and 0.19 lm W^−1^ and 13.38, 23.84, 36.20, and 49.54 cd m^−2^ for 1.PF_6_, 2.PF_6_, 4.PF_6_ and 6.PF_6_ devices (Fig. 3e and f). This is nicely reflected in the device stability studied using the total emitted energy (*E*_tot_) that is calculated by integrating the radiant flux curve until the time needed to reach one-fifth of the maximum luminance as an upper limit definition (Fig. 3i).^22^ Here, *E*_tot_ gradually increases from 0.47 mJ to 6.75 mJ upon increasing the alkyl length chain (Fig. 3i). Indeed, Fig. 3j displays a clear enhancement of the device efficiency and stability with the increase of the alkyl chain as expected by the higher and stable thin-film PLQY under working conditions – *vide supra*.

However, a high PLQY does not directly imply the best performing device, since charge trapping and/or electrochemical doping critically alter the preferred composition of the active material.^41^ In this context, the enhanced reversibility of the electrochemical processes keeping a good ion polarizability in thin films upon increasing the alkyl chain is also key towards circumventing hole injection/transport limitations.

To further confirm the benefits of the AIE behaviour, we prepared another set of devices using an inert polymer matrix to disrupt aggregation, that is, adding 20 wt%. of polymethylmethacrylate (PMMA) with respect to the best emitting molecule 6.PF_6_ (see Experimental section for details).^42^ As showed in Fig. 3k, the device performance with PMMA decreases considerably with a luminance from 49.54 (pristine) to 3.77 (with PMMA) cd m^−2^, and an efficiency from 0.19 (pristine) to 0.022 (with PMMA) lm W^−1^, corroborating that the aggregation induced emission is, indeed, crucial for this type of emitters.

## Conclusions

In summary, two different families of positively charged structures derived from TPPE were successfully synthetized, and the one with optimized performance applied in ion-based lighting devices, such as LECs, to rationalize the effect of the hydrophobic/hydrophilic forces (represented by the positively charged pyridinium moieties and the increasing chain length and counterion, respectively) that govern the AIE behaviour. For instance, halide AIE series require C_12_ side chain lengths to overcome the inherent electrostatic repulsions and self-aggregation, while PF_6_^−^ AIE series increases the hydrophobic nature promoting the aggregate formation and gradually enhancing their emitting properties from C2 up to C6 side chains. This was nicely corroborated by the gradual PLQY increase going from 1.PF_6_ to 6.PF_6_ films. In addition, a reasonable electrochemical behaviour that allows their integration in LECs was also noted in the PF_6_^−^ series. Here, the devices featured a gradually enhanced brightness, efficiency and stability going from 1.PF_6_ to 6.PF_6_ emitters, indicating that the AIE feature is beneficial. Indeed, EIS assays corroborated that the lock structure of AIE emitters allows and efficient ion polarization regardless of their neutral or ionic nature;^23^ a question that has always discouraged the community to apply this type of materials in LECs. Finally, we also state that the AIE feature is key towards meeting high performance by using PMMA additive that disrupt the formation of aggregates LECs and, in turn, dramatically reduced the device performance of 6.PF_6_ AIE emitter. Overall, this work rationalizes a design guideline for ionic AIE emitters with fine-tuned colloidal stability, encouraging their future application in water-based applications such nanomedicine or sensor or, as in this case, light-management technologies like lighting emitting electrochemical cells.

## Data availability

A initial version of the manuscript was deposited on ChemRxiv, DOI: 10.26434/chemrxiv-2023-v464k.

## Author contributions

The experimental work was carried out mainly by ASV (synthesis and structural characterization of molecules, in solution optical properties and size studies of the aggregates by DLS), OA-R (thin films characterization, device fabrication), AK (spectroscopy), SL (electrochemical characterization), and LMC (electrochemical impedance spectroscopy). Project conceptualization and main funding acquisition: EA-P and RDC. Project discussion, experimental design, data analysis: EA-P, MAK and RDC. The manuscript was written through contributions of all authors. All authors have given approval to the final version of the manuscript.

## Conflicts of interest

There are no conflicts to declare.

## Supplementary Material

SC-015-D3SC05941C-s001
