# Supplementary material for: Controlling aggregation-induced emission by supramolecular interactions and colloidal stability in ionic emitters for light-emitting electrochemical cells
Source: Chem Sci. 2024 Jan 25;15(8):2755–62. doi: 10.1039/d3sc05941c (PMC10882460; doi:10.1039/d3sc05941c)
Supplement: SC-015-D3SC05941C-s001 [file SC-015-D3SC05941C-s001.pdf]

Electronic Supplementary Material (ESI) for Chemical Science.
This journal is © The Royal Society of Chemistry 2024

Supporting Information

Controlling Aggregation-Induced Emission by Supramolecular Interactions and Colloidal Stability in Ionic Emitters for Light-Emitting Electrochemical Cells

Alba Sanz-Velasco,† Olivia Amargós-Reyes§, Aya Kähäri,† Sophia Lipinski, § Luca M. Cavinato, § Rubén D. Costa*§, Mauri A. Kostiainen,† E. Anaya-Plaza *†

†*Department of Bioproducts and Biosystems, Aalto University, 02150 Espoo (Finland)*

§*Chair of Biogenic Functional Materials, Technical University of Munich, 94315 Straubing (Germany)*

***Corresponding authors**: ruben.costa@tum.de; eduardo.anaya@aalto.fi

Table of contents

Materials and Equipment S3
Methods S3
UV-visible spectrophotometry (UV-vis) and fluorescence measurements S3
Table of solubilities S3
Dynamic light scattering (DLS) S4
Cyclic voltammetry S4
Thin Film Preparation and Characterization S4
Device Fabrication and Characterization S5
Synthetic protocols S6
Synthesis of TPPE S6
Synthesis of halide TPPE-alkyl salts S7
1.I S7
2.Br S11
4.I S14
6.Br S18
12.Br S22
Synthesis of PF6 TPPE-alkyl salts S26
1.PF6 S26
2.PF6 S30
4.PF6 S34
6.PF6 S37
12.PF6 S40
Additional absorbance and emission experiments S44
Additional DLS experiments S48
Square wave voltammetry S49
Normalized absorbance spectra of films in MeCN S49
Films morphology by AFM S50
Electrochemical impedance spectroscopy (EIS) S50
Bibliography S51

Materials and Equipment

The reagents and solvents used for synthesis were bought from commercial suppliers and directly used without further purification: 1,1,2,2-Tetrakis(4-bromophenyl)ethylene (TCI-chemicals), Bis(triphenyl-phosphine)palladium(II) dichloride (Sigma Aldrich), potassium carbonate (Sigma Aldrich), dimethylformamide (Sigma Aldrich), ammonium hexafluorophosphate (Fisher Scientific), iodomethane (Sigma Aldrich), bromoethane (Sigma Aldrich), 1-iodobutane (Sigma Aldrich), 1-bromohexane (Sigma Aldrich) and 1-bromododecane (Sigma Aldrich). The water used in all the experiments was Milli-Q grade and all solvents were purchased in Sigma Aldrich.

Thin layer chromatography (TLC) was performed with aluminum sheets coated with silica gel 60 F254 (Merck). TLC plates were inspected by UV light (λ = 254 nm, 365 nm). Silica gel column chromatography was carried on with Si 60 Å (particle size 40–63 µm). Photophysical studies were performed using a spectrophotometer (Varian Cary 50 UV-Vis) and a fluorometer (Cary Eclipse Fluorescence Spectrophotometer). NMR results were obtained with Bruker NMR Spectrometer AV NEO 400 and at 293 K, unless stated otherwise. Chemical shifts are indicated in ppm and are referenced relative to deuterated solvent residual peaks. Multiplicities are explained with the following abbreviations: m (multiplet), q (quadruplet), t (triplet), d (doublet), s (singlet). Mass spectrometry was carried out with Agilent HPLC-QTOF Dual ESI.

Methods

UV-visible spectrophotometry (UV-vis) and fluorescence measurements

Stock solutions were prepared by dissolving the different molecules in the organic solvent (see Table S1). In all cases the dye from the stock solution was added over milliQ water and mixed energetically to prepare the different samples. Organic solvents were measured with Hamilton syringes to reduce evaporation. Absorbance studies were done with quartz cuvettes of 1 mm or 10 mm pathlength depending on the sample concentration. Fluorescence measurements were carried out with quartz cuvettes of 10 mm pathlength. All emission data presented have been recorded in slit 5, unless stated otherwise.

Table of solubilities

Table S1. Solubilities of the halide TPPE-alkyl series and PF_6 TPPE-alkyl series.

Molecule	Solvent
**1.I**	MeOH/Water
**2.Br**	MeOH
**4.I**	MeOH
**6.Br**	MeOH
**12.Br**	MeOH

Molecule	Solvent
**1.PF$_6$**	MeCN
**2.PF$_6$**	Acetone
**4.PF$_6$**	MeCN
**6.PF$_6$**	MeCN
**12.PF$_6$**	MeCN

Dynamic light scattering (DLS)

Size distribution (d. nm) of the aggregates was measured using a Malvern Instruments DLS device (Zetasizer Nano ZS Series) with a 4 mW He-Ne ion laser at a wavelength of 633 nm and an avalanche photodiode detector at an angle of 173°. All experiments were carried at room temperature with PMMA cuvettes. Zetasizer software (Malvern Instruments) was used to obtain the particle size distributions. The sample preparation is the same as described in UV-vis spectrophotometry and fluorescence measurements.

Cyclic voltammetry

Square wave voltammetry measurements were carried out in a cylindrical one compartment cell with a three-electrode set up. A platinum disk working electrode with a diameter of 3.0 mm, a Pt wire as counter and Ag wire as quasi reference electrodes were used. Prior to every measurement, the working electrode was polished with Alumina slurry 0.04 mm from Schmitz Metallographie GmbH. The potential was controlled by a Metrohm μAutolabIII potentiostat. All measurements were conducted at 100 mV/s under inert nitrogen atmosphere with dry and degassed acetonitrile containing 0.1 M tetrabutylammonium hexafluorophosphate as supporting electrolyte. Ferrocene was added as internal standard to reference the acquired data to $Cp_2Fc^{+/0}$.

Thin Film Preparation and Characterization

The solutions for each molecule were prepared in MECN at a concentration of 20 mg/mL, stirred for 15 min and then filtered with a 0.22 µm PTFE filter. Thin films were prepared from the filtered solution by spin-coating, in a previously cleaned quartz substrate, at 800 rpm for 30 s and 1500 rpm for 30 s.

AFM measurements were acquired using an MFP-3D Origin+ (Asylum Research), and further elaborate with Gwyddion evaluation software. Absorption spectra were recorded with a Shimadzu UV-vis/2600 spectrometer.

The photoluminescence spectra and PLQY values in thin film were measured with an FS5 Spectrofluorometer with integrating sphere SC-30 (Edinburgh Instruments). Photoluminescence measurements at temperatures ranging from 298 to 360 K were performed upon enclosing the thin film in an Optistat-DN (Oxford Instruments) equipped with a temperature controller. The excited-state lifetimes (τ) were acquired with a TCSPC diode at λ 280 nm and their average value can be obtained using the following equation:

$$\tau = \frac{A_1\tau_1^2 + A_2\tau_2^2}{A_1\tau_1 + A_2\tau_2}$$

Device Fabrication and Characterization

ITO substrates were purchased from Naranjo Substrates with an ITO thickness of 130 nm. They were extensively cleaned using detergent, water, ethanol, and propan-2-ol as solvents in an ultrasonic bath (frequency 37–70 Hz) for 15 min each. Afterward, the substrates were dried with inert gas and put in a UV–ozone cleaner for 15 min. The clean substrates were coated, via spin-coating, with 80 µL of an aqueous solution of PEDOT:PSS mixed with propan-2-ol in a ratio of 3:1 and filtered with a 0.45 µm filter using a rotation speed of 1000 rpm for 60 s and 2500 rpm for 20 s. The resulting layers (thickness of ~70 nm) were dried on a hotplate at 120 °C for 15 min. The active layers were deposited via spin-coating at 800 rpm for 30 s and at 1500 rpm for 30 s, resulting in 70–90 nm thick film. After the deposition of the active layer, the devices were dried under vacuum for 50 min and transferred to an inert atmosphere glovebox. Finally, aluminum cathode (100 nm) was thermally evaporated onto the active layer using a shadow mask under high vacuum (<10–6 mbar) in an Angstrom Covap evaporator integrated into the inert atmosphere glovebox.

For the devices with PMMA, a solution of PMMA was made with a concentration of 5 mg/mL in MeCN and filtered with a 0.22 µm filter. Then 25% of this solution in mass ratio was added to the AIE1.6-PF6 solution. The rest of the process was the same as the explained above.

Time dependence of luminance, voltage, and current was measured by applying constant and/or pulsed voltage and current by monitoring the desired parameters simultaneously by using Avantes spectrophotometer (Avaspec-ULS2048L-USB2) in conjunction with a calibrated integrated sphere Avasphere 30-Irrad and Botest OLT OLED Lifetime-Test System. Electroluminescence spectra were recorded using the above-mentioned spectrophotometer.

Electrochemical impedance spectroscopic assays (EIS) were carried out with a potentiostat/galvanostatic (Metrohm µAutolabIII) equipped with a frequency response analyzer module (FRA2). Measurements were performed at the applied voltage of 0-5 V and fitted with the Nova 2.1 software using the circuit model shown in Figure S70. RLEC represents the effective resistance of the active layer, including injection resistances, CPELEC represents the capacitance of the two electrodes contacting the film. The second resistor (Rseries) represents all external resistances, including those of the ITO and Al electrodes, and Lcables represents the inductance of the measurement cables. The AC signal amplitude was set to 10 mV, modulated in a frequency range from 10 to 106 MHz. Ionic conductivity and dielectric constant are extrapolated from the fitted data following the procedure reported elsewhere.[1]

Synthetic protocols

Synthesis of TPPE. Minor adjustments were done to the protocol described by A. Rananaware.[2] 500 mg of Br-TPE (0.77 mmol) were mixed with 1015 mg of 4-Pyridylboronic acid pinacol ester (4.94 mmol, 1.6 eq) in a round bottomed flask and degassed for 15 min using argon. 4.4 mL of 2 M K_2CO_3 solution were deoxygenated for 15 min with argon stream, as well as 7.1 mL (9.2 mL/mmol) of DMF. Both liquids were added into the round-bottomed flask and the mixture was stirred for 15 min. Then, 265 mg of $PdCl_2(PPh_3)_2$ (0.38 mmol, 0.12 eq) were incorporated into the reaction. The final mixture was heated at 120 ºC for 24 h. TLC analysis was used to follow the reaction completion using as a mobile phase DCM/THF (8/2, v/v) + 1 % Et_3N. Solvent was evaporated under reduced pressure and the solid obtained was dissolved in a mixture of water and DCM. Aqueous phase was washed several times with DCM (3 × 10 mL). The organic layers were washed with brine and dried over with $MgSO_4$, filtered and evaporated under vacuum. The final solid was purified by chromatography column using as eluent DCM/THF (100/0 to 0/100, v/v) to afford 295 mg of TPPE (48 % yield). **^{1}H NMR** (400 MHz, $CDCl_3$): δ (ppm) = 8.65 (dd, J = 4.6, 1.5 Hz, 8 H), 7.50 (dd, J = 5.0, 3.3 Hz, 16 H), 7.25 (d, J = 8.3 Hz, 8 H).

HR-MS (ESI) m/z: $[M]^+$ calcd for $C_{46}H_{32}N_4$, 640.26; found, 641.27 $[M+H]^+$

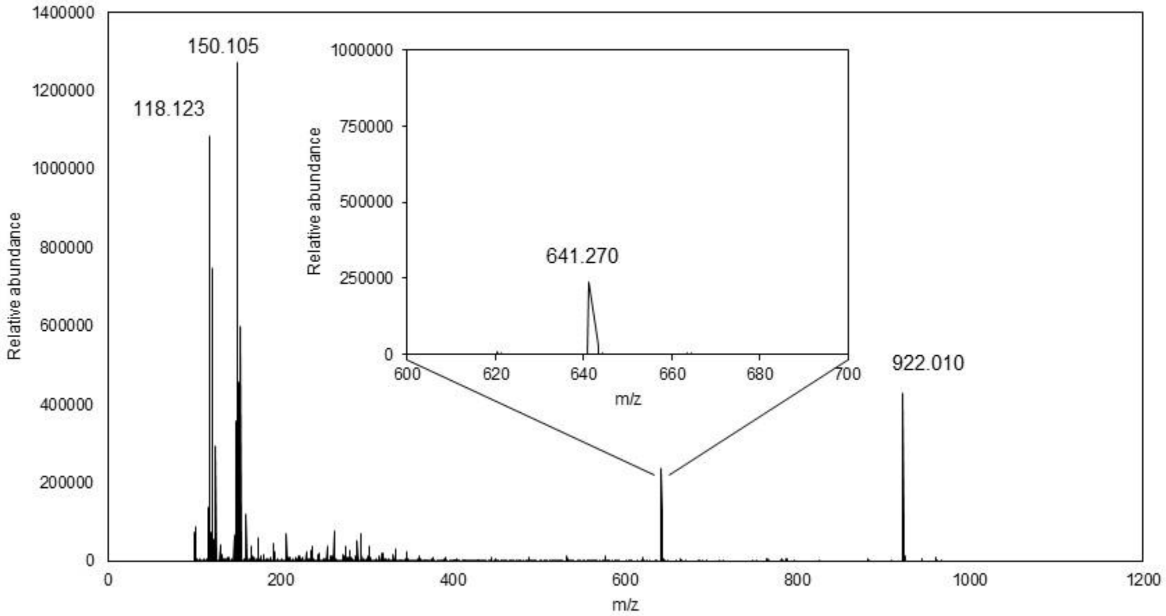

Fig. S1. ESI mass spectrum of TPPE.

Synthesis of halide TPPE-alkyl salts. Depending on the length of the alkyl chain two different conditions were used.

Condition (a) for 1.I and 2.Br. 25 mg of TPPE (0.04 mmol) were dissolved in DMF (26 mL/mmol) and heated at 90 ºC until complete solution. Following by the addition of the corresponding alkyl halide (0.4 mmol, 2.5 eq). Reaction was heated for 4 h. A solid precipitated in the reaction mixture. Reaction was allowed first to cool at room temperature and then, it was quenched with MeOH. Solvent was removed under reduced pressure and the final solid was cleaned by crushing it with Et_2O, followed by filtration.

1.I: 4,4',4",4"'-(ethene-1,1,2,2-tetrayltetrakis(benzene-4,1-diyl))tetrakis(1-methylpyridin-1-ium) iodine with a 54 % yield. **^{1}H NMR** (400 MHz, DMSO-d_6): δ (ppm) = 8.97 (d, J = 6.9 Hz, 8H), 8.46 (d, J = 7.0 Hz, 8H), 8.01 (d, J = 8.5 Hz, 8H), 7.39 (d, J = 8.4 Hz, 8H), 4.32 (s, 12H). **^{13}C NMR** (400 MHz, DMSO-d_6): δ (ppm)= 153.6, 146.4, 146.1, 132.6, 132.5, 128.5, 124.3, 47.6

HR-MS (ESI) m/z: $[M]^+$ calcd for $C_{50}H_{44}I_4N_4$, 1207.97; found, 1081.07 $[M-I]^+$

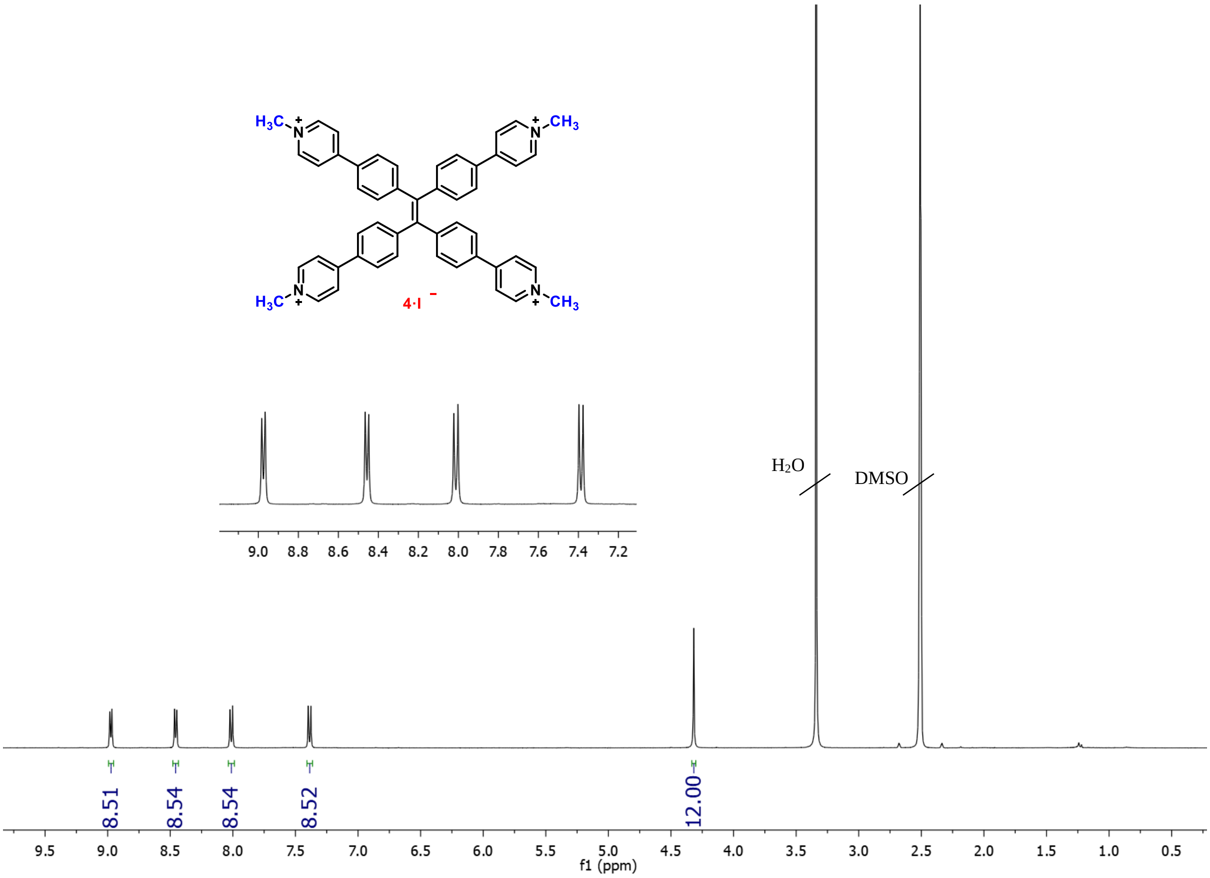

Fig. S2. ^{1}H-NMR (400 MHz, DMSO-d_6) spectrum of **1.I.**

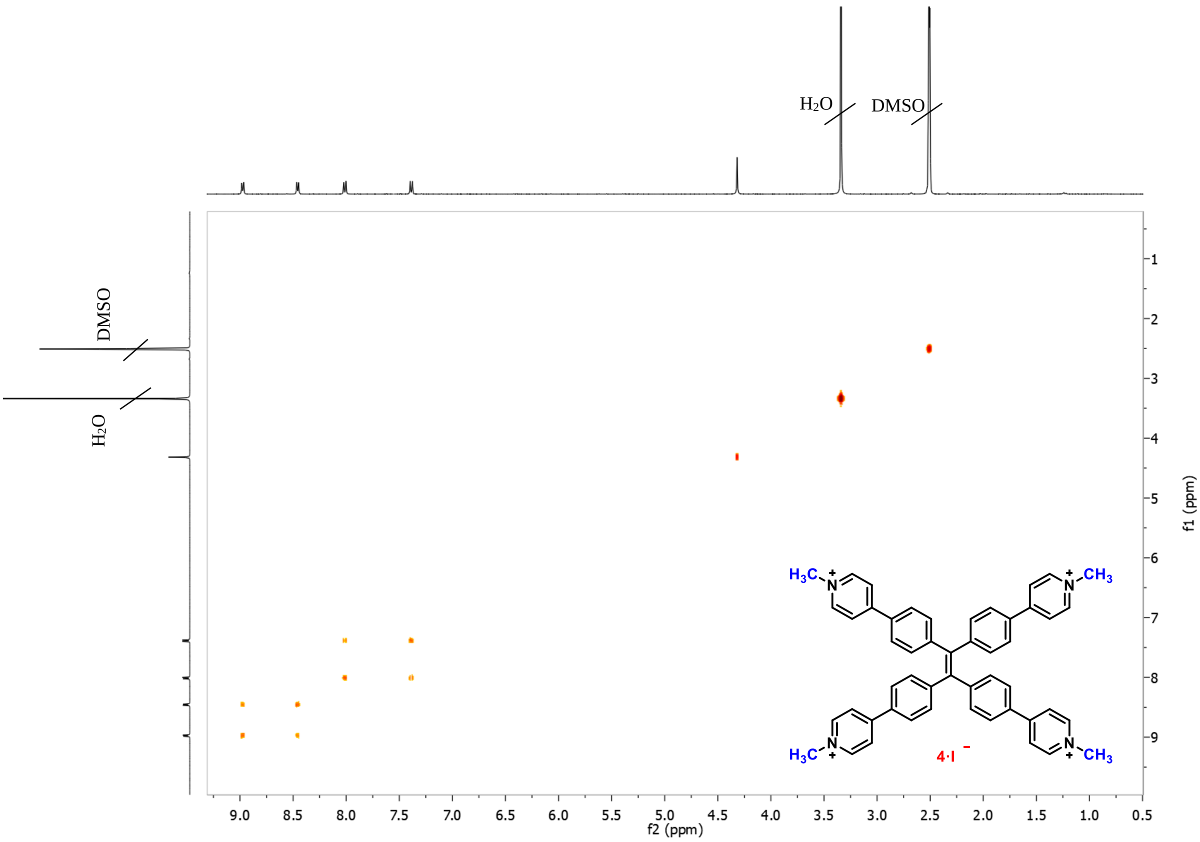

Fig. S3. COSY NMR (DMSO-d_6) spectrum of **1.I.**

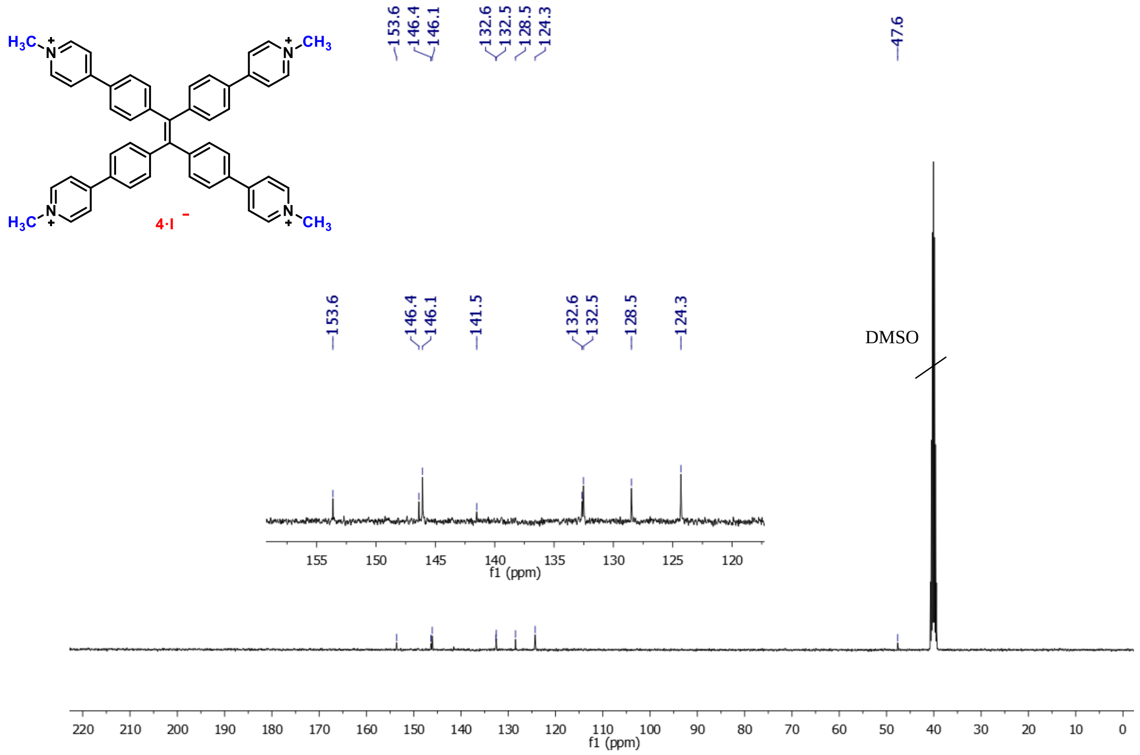

Fig. S4. ^{13}C-NMR (DMSO-d_6, 100MHz) spectrum of **1.I**

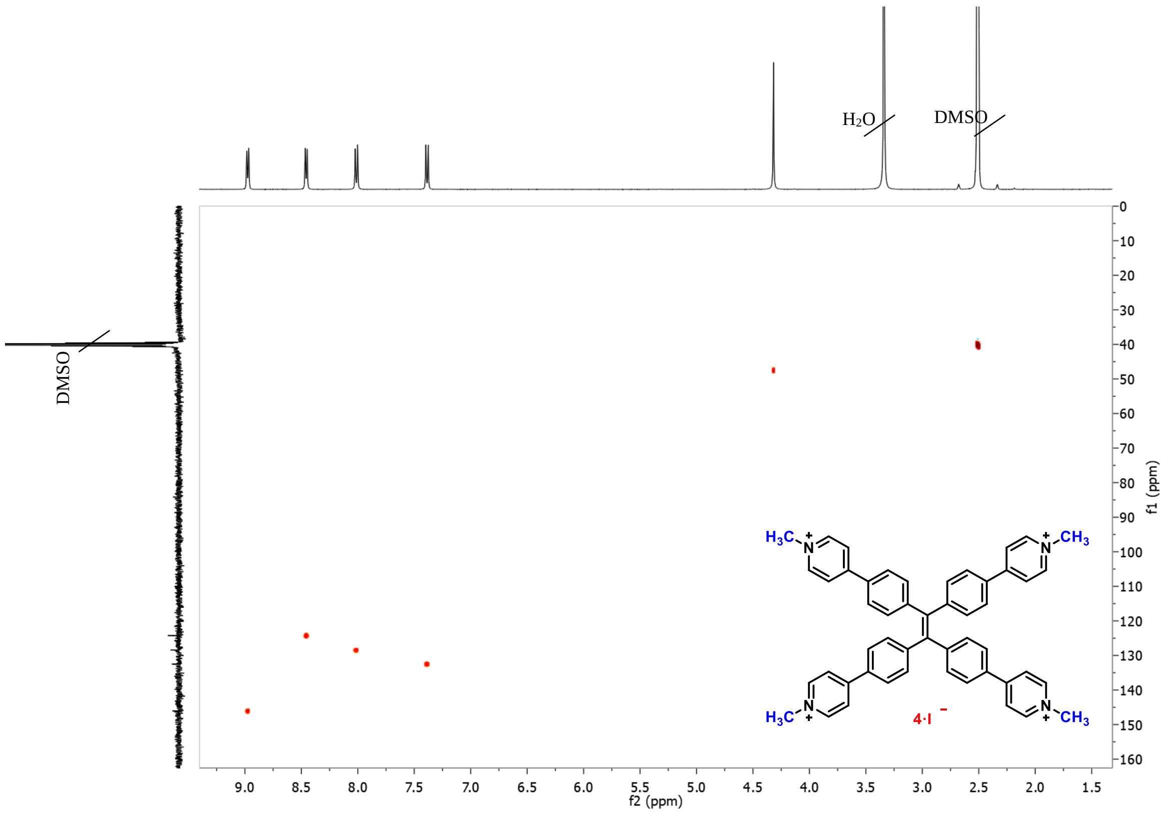

Fig. S5. HSQC (DMSO-d$_6$) spectrum of **1.I.**

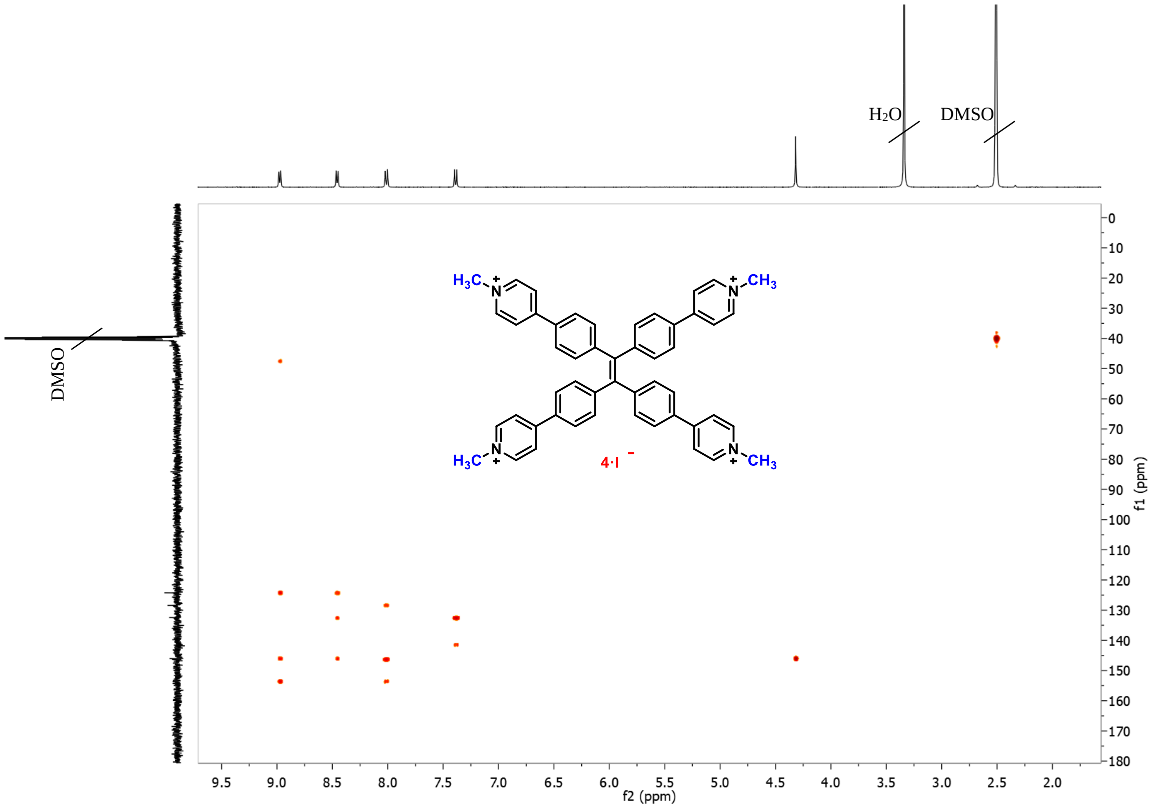

Fig. S6. HMBC (DMSO-d$_6$) spectrum of **1.I.**

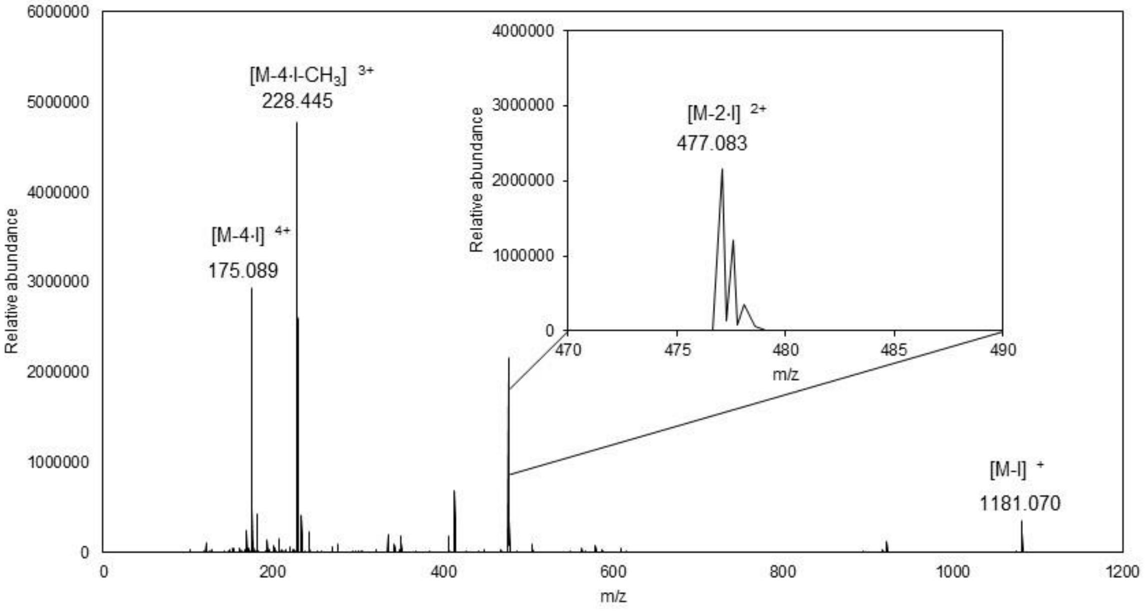

Fig. S7. ESI mass spectrum of **1.I.**

2.Br: 4,4',4'',4'''-(ethene-1,1,2,2-tetrayltetrakis(benzene-4,1-diyl))tetrakis(1-ethylpyridin-1-ium) bromide with a 62 % yield. **^{1}H NMR** (400 MHz, DMSO-d$_6$): δ (ppm) = 9.09 (d, J = 7.0 Hz, 8H), 8.48 (d, J = 7.0 Hz, 8H), 8.01 (t, J = 7.9 Hz, 8H), 7.39 (d, J = 8.5 Hz, 8H), 4.61 (q, J = 7.2 Hz, 8H), 1.54 (t, J = 7.3 Hz, 12H). **^{13}C NMR** (400 MHz, DMSO-d$_6$): δ (ppm)= 153.9, 146.3, 145.0, 141.5, 132.6, 132.5, 128.5, 124.7, 55.9, 16.8

HR-MS (ESI) m/z: $[M]^+$ calcd for $C_{54}H_{52}Br_4N_4$, 1072.09; found, 458.13 $[M\text{-}2{\cdot}Br]^+$

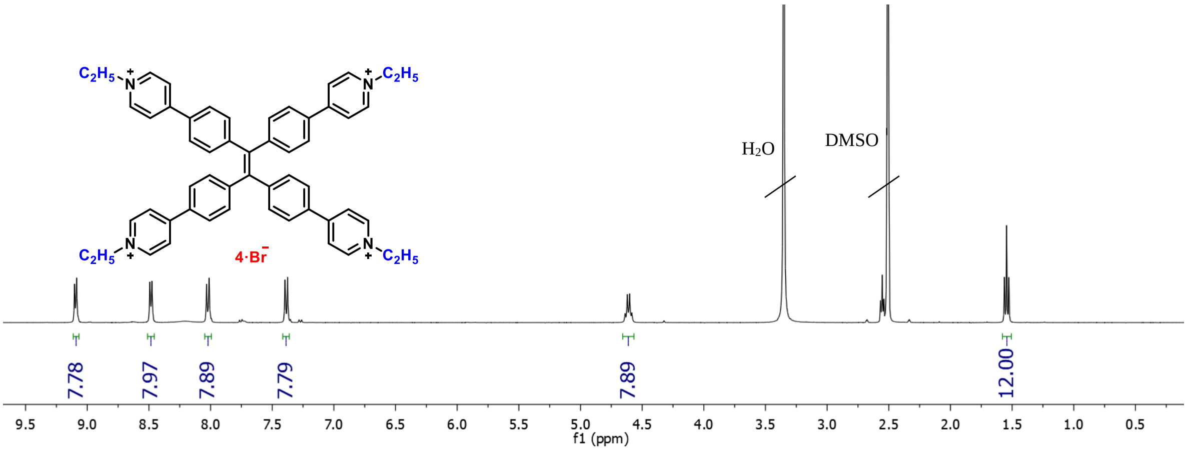

Fig. S8. ^{1}H-NMR (DMSO-d$_6$, 400 MHz) spectrum of **2.Br.**

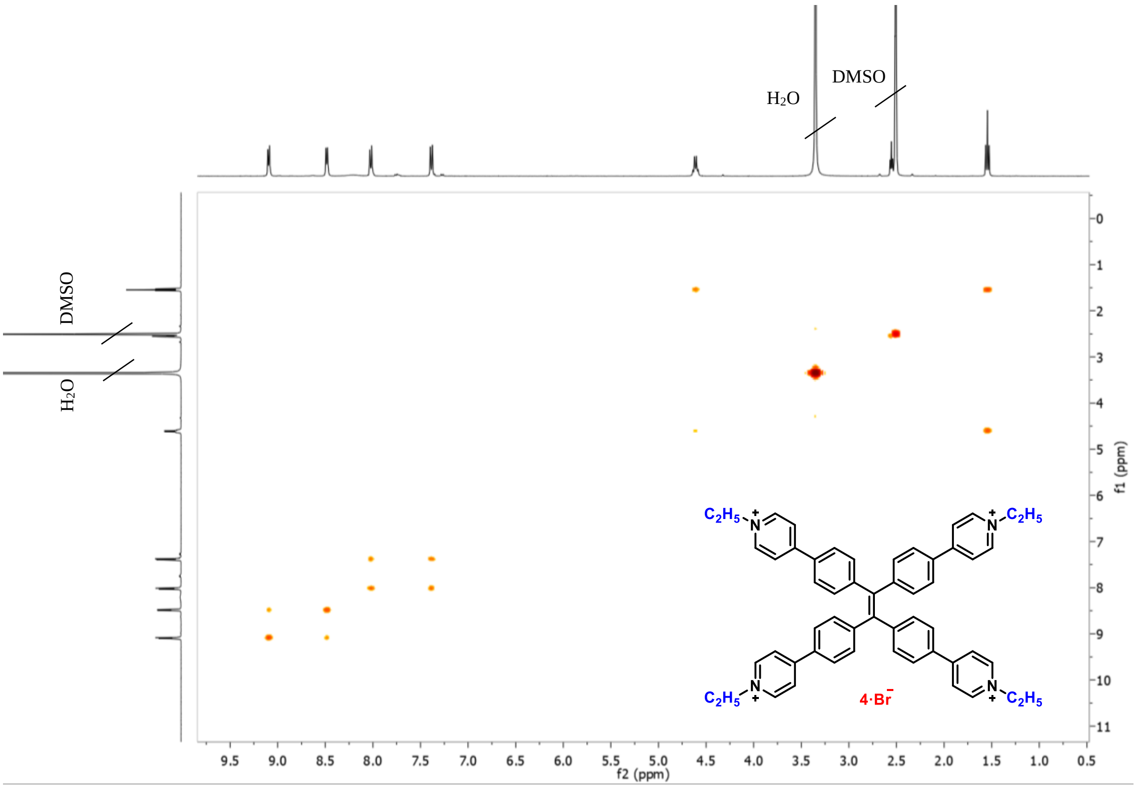

Fig. S9. COSY NMR (DMSO-d$_6$) spectrum of **2.Br.**

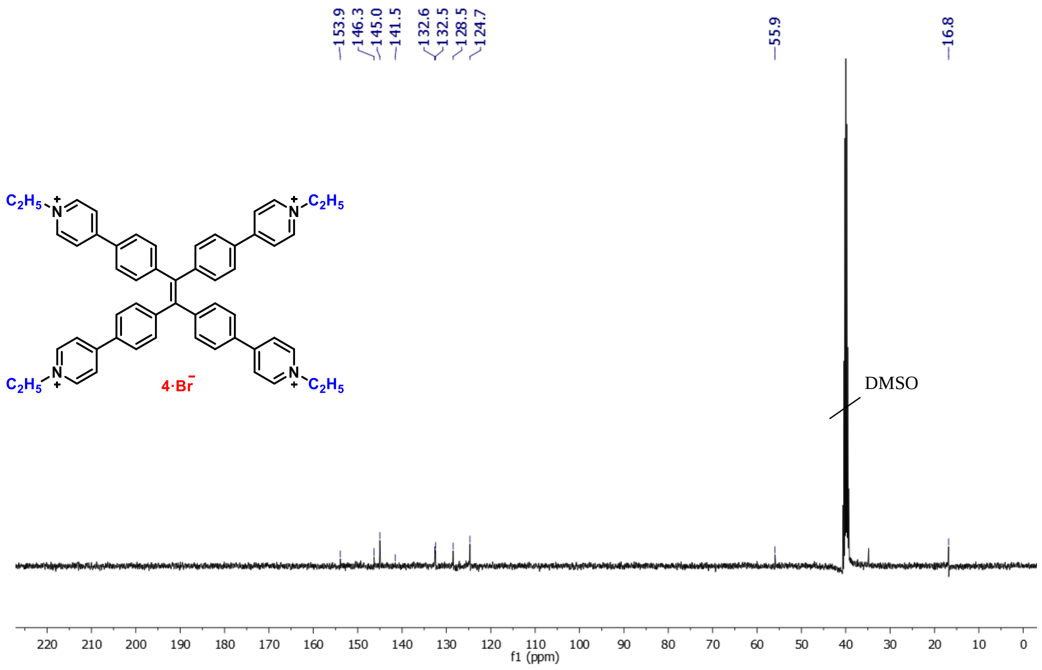

Fig. S10. ^{13}C-NMR (DMSO-d_6, 100 MHz) spectrum of **2.Br.**

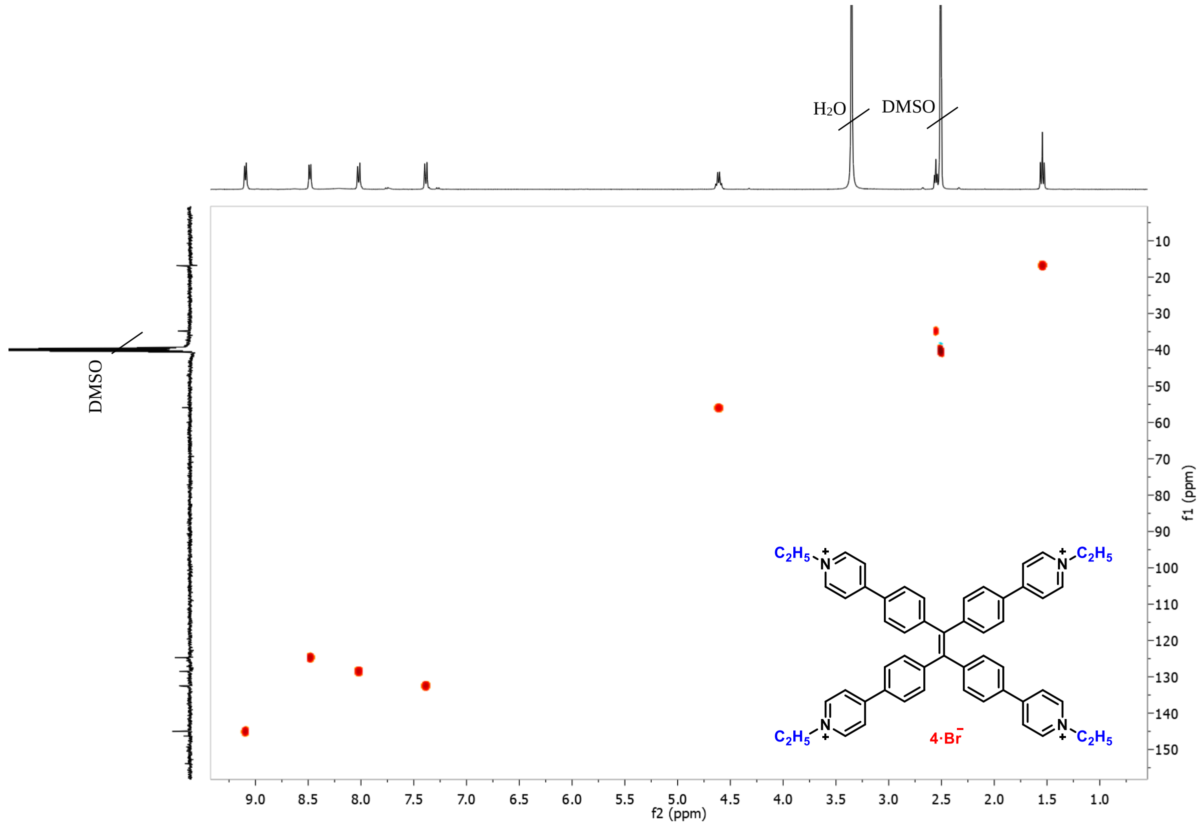

Fig. S11. HSQC (DMSO-d_6) spectrum of **2.Br.**

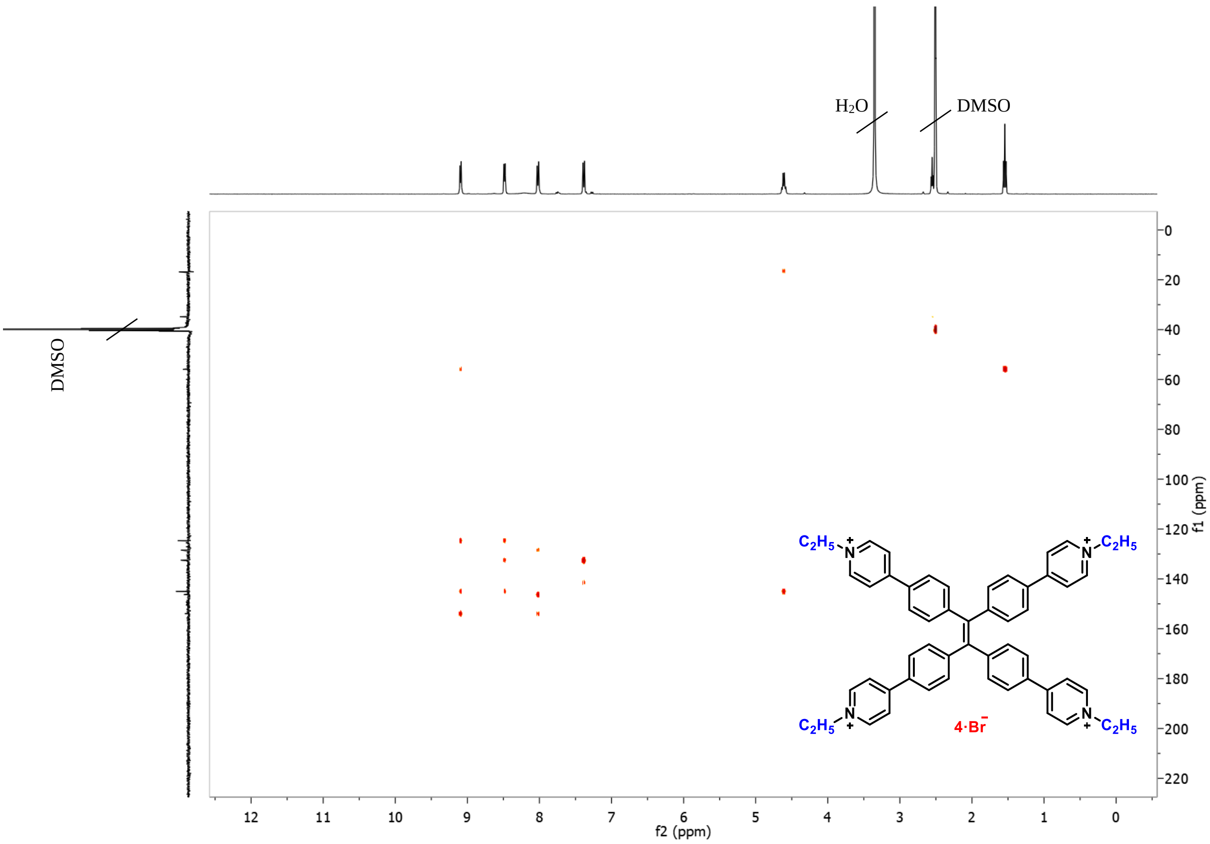

Fig. S12. HMBC (DMSO-d_6) spectrum of **2.Br.**

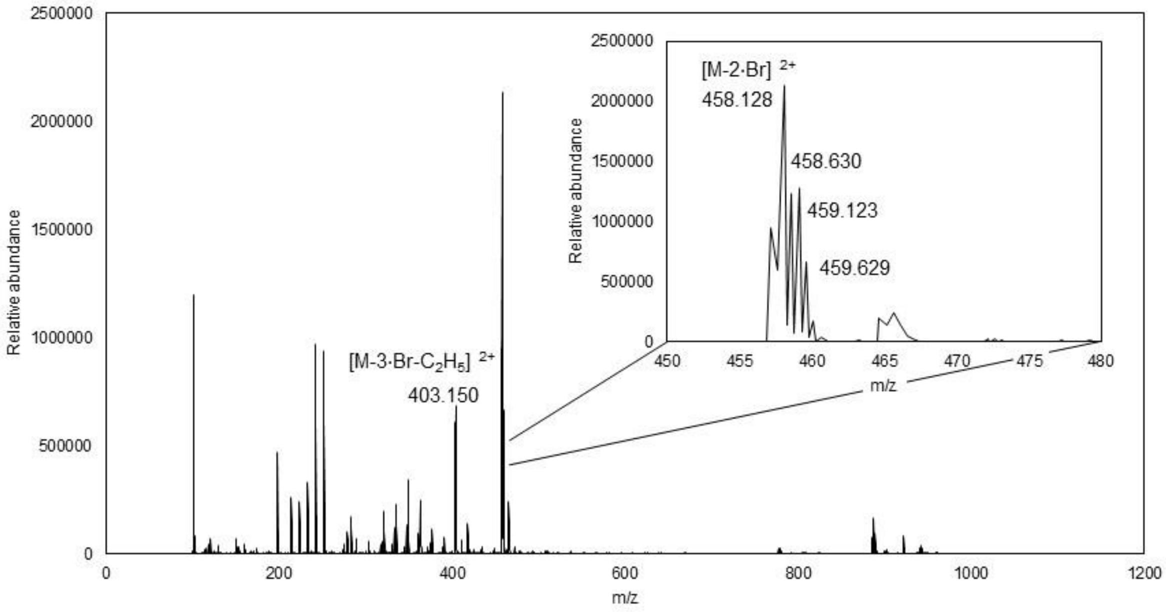

Fig. S13. ESI mass spectrum of **2.Br**

Condition (b) for 4.I, 6.Br and 12.Br. 25 mg of TPPE (0.04 mmol) were dissolved in DMF (26 mL/mmol) and heated at 120 ºC until complete solution. Following by the addition of the corresponding alkyl halide (0.4 mmol, 2.5 eq). Reaction was heated overnight. A solid precipitated in the reaction mixture. Reaction was allowed first to cool at room temperature and then, it was quenched with MeOH. Solvent was removed under reduced pressure and the final solid was cleaned by crushing it with Et_2O, followed by filtration. Additional purification by size-exclusion chromatography (BioBeads in CH_2Cl_2) was carried out to remove small impurities of **12.Br**.

4.I: 4,4',4'',4'''-(ethene-1,1,2,2-tetrayltetrakis(benzene-4,1-diyl))tetrakis(1-buthylpyridin-1-ium) iodine. with a 71 % yield. **^{1}H NMR** (400 MHz, DMSO-d_6): δ (ppm) = 9.06 (d, J = 7.0 Hz, 8H), 8.47 (d, J = 7.0 Hz, 8H), 8.01 (d, J = 8.5 Hz, 8H), 7.38 (d, J = 8.5 Hz, 8H), 4.57 (t, J = 7.4 Hz, 8H), 1.90 (m, 8H), 1.29 (dt, J = 14.6, 7.4 Hz, 8H), 0.92 (t, J = 7.4 Hz, 12H). **^{13}C NMR** (400 MHz, DMSO-d_6): δ (ppm)= 154.0, 146.4, 145.2, 141.6, 132.7, 132.5, 128.6, 124.8, 60.2, 33.1, 19.3, 13.9

HR-MS (ESI) m/z: $[M]^+$ calcd for $C_{62}H_{68}I_4N_4$, 1376.16; found, 1249.26 $[M\text{-}I]^+$

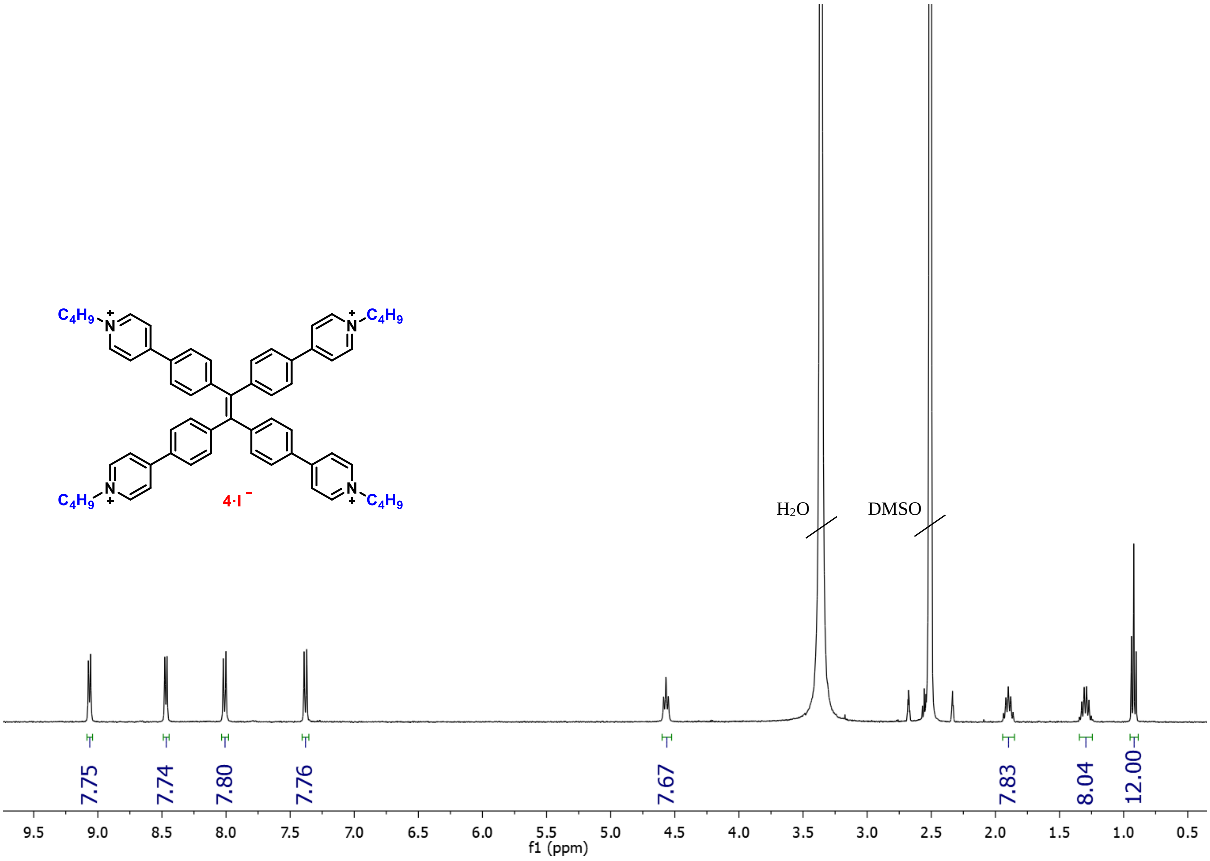

Fig. S14. ^{1}H-NMR (DMSO-d6, 400 MHz) spectrum of **4.I**

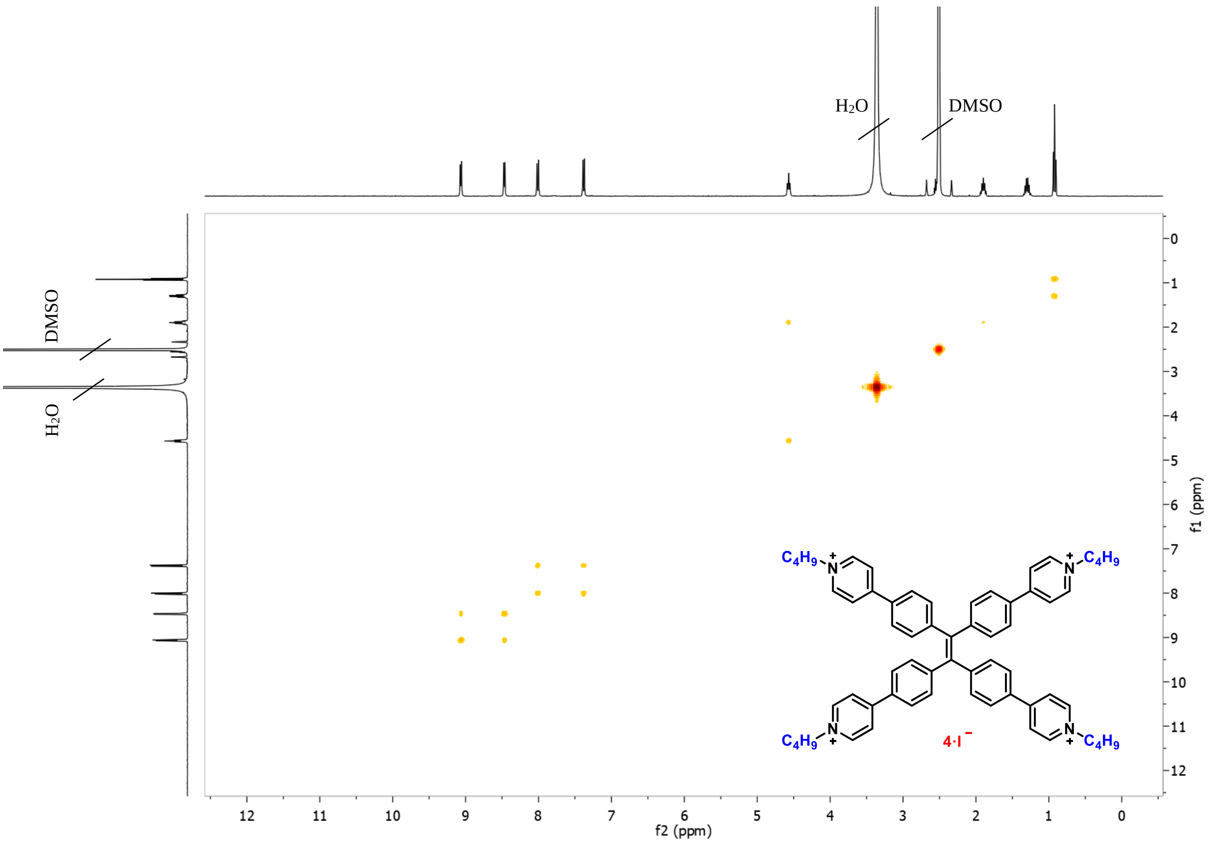

Fig. S15. COSY NMR (DMSO-d$_6$) spectrum of **4.I**

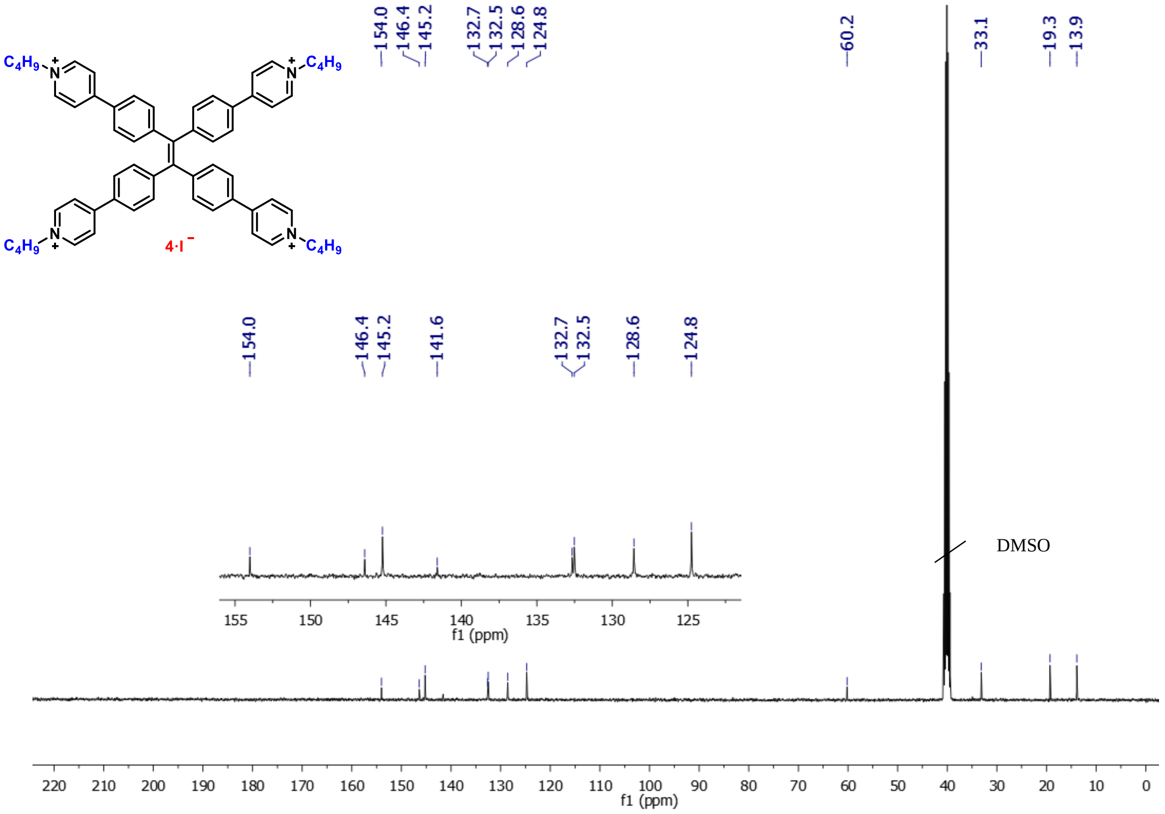

Fig. S16. ^{13}C-NMR (DMSO-d$_6$, 100 MHz) spectrum of **4.I.**

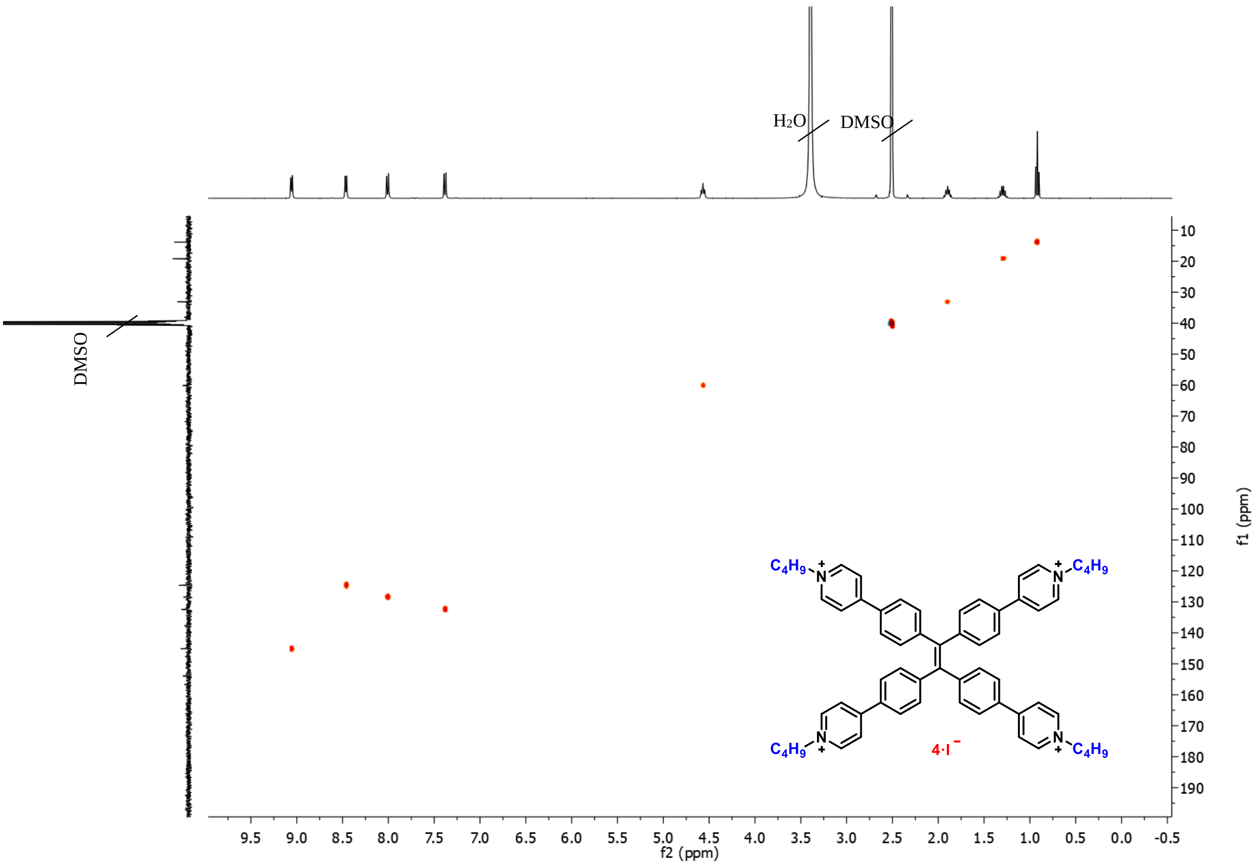

Fig. S17. HSQC (DMSO-d$_6$) spectrum of **4.I.**

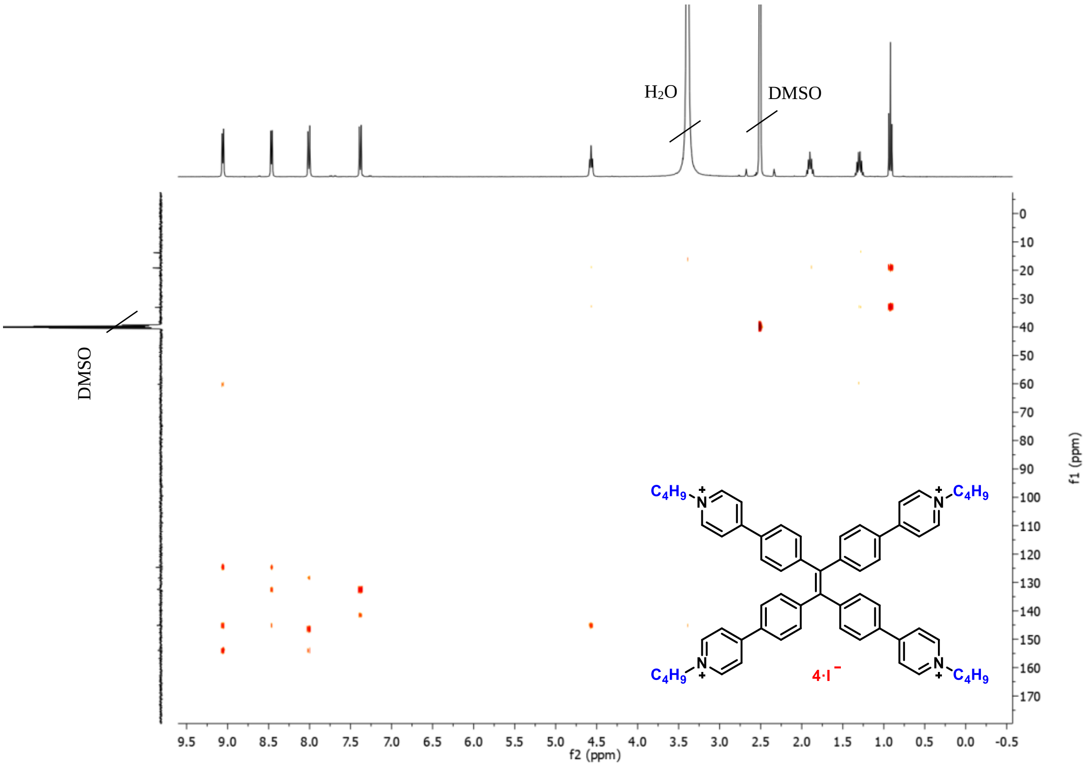

Fig. S18. HMBC (DMSO-d$_6$) spectrum of **4.I.**

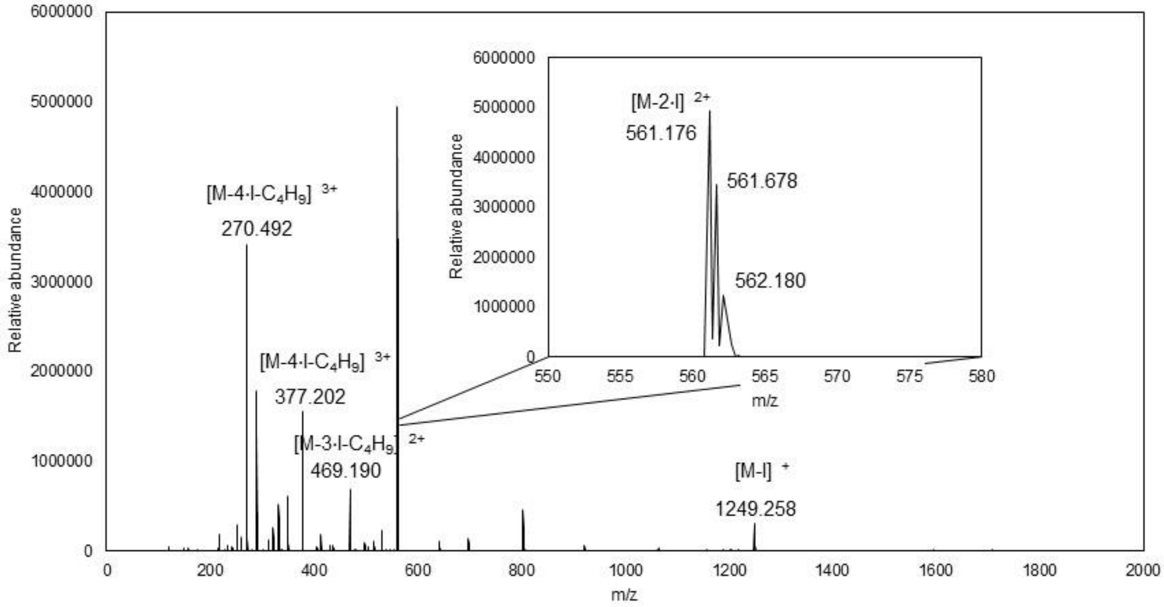

Fig. S19. ESI mass spectrum of **4.I**

6.Br: 4,4',4'',4'''-(ethene-1,1,2,2-tetrayltetrakis(benzene-4,1-diyl))tetrakis(1-hexylpyridin-1-ium) bromine, with a 60 % yield. ^{1}**H NMR** (400 MHz, DMSO-d$_6$): δ (ppm) = 9.06 (d, J = 6.2 Hz, 8H), 8.47 (d, J = 6.4 Hz, 8H), 8.01 (d, J = 8.4 Hz, 8H), 7.38 (d, J = 8.4 Hz, 8H), 4.56 (t, J = 7.1 Hz, 8H), 1.91 (s, 9H), 1.28 (s, 28H), 0.86 (t, J = 6.8 Hz, 14H). ^{13}**C NMR** (400 MHz, DMSO-d$_6$): δ (ppm)= 153.5, 145.9, 144.8, 141.1, 132.2, 132.0, 128.1, 124.3, 59.8, 30.7, 30.6, 25.1, 21.9, 13.9

HR-MS (ESI) m/z: $[M]^+$ calcd for $C_{70}H_{84}Br_4N_4$, 1296.34; found, 570.25 $[M\text{-}2{\cdot}Br]^+$

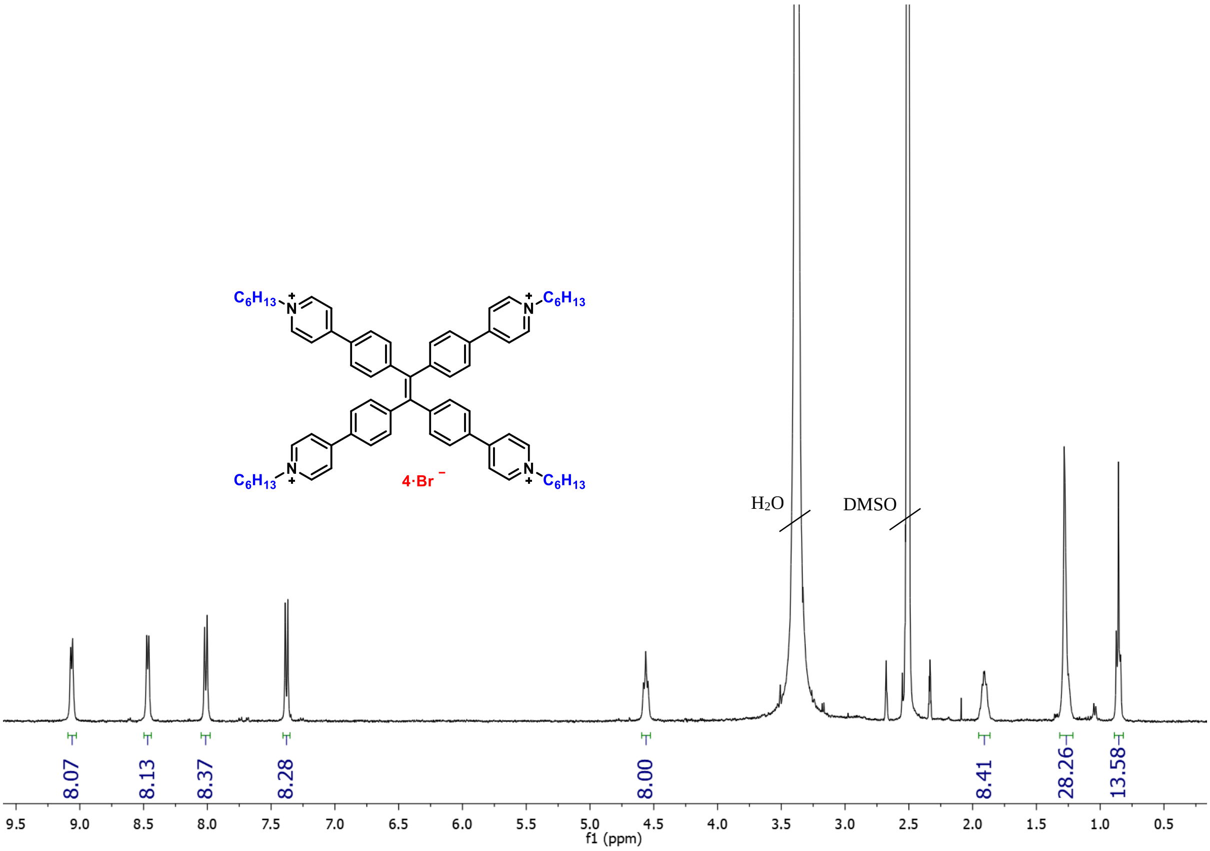

Fig. S20. ^{1}H-NMR (DMSO-d6, 400 MHz) spectrum of **6.Br**

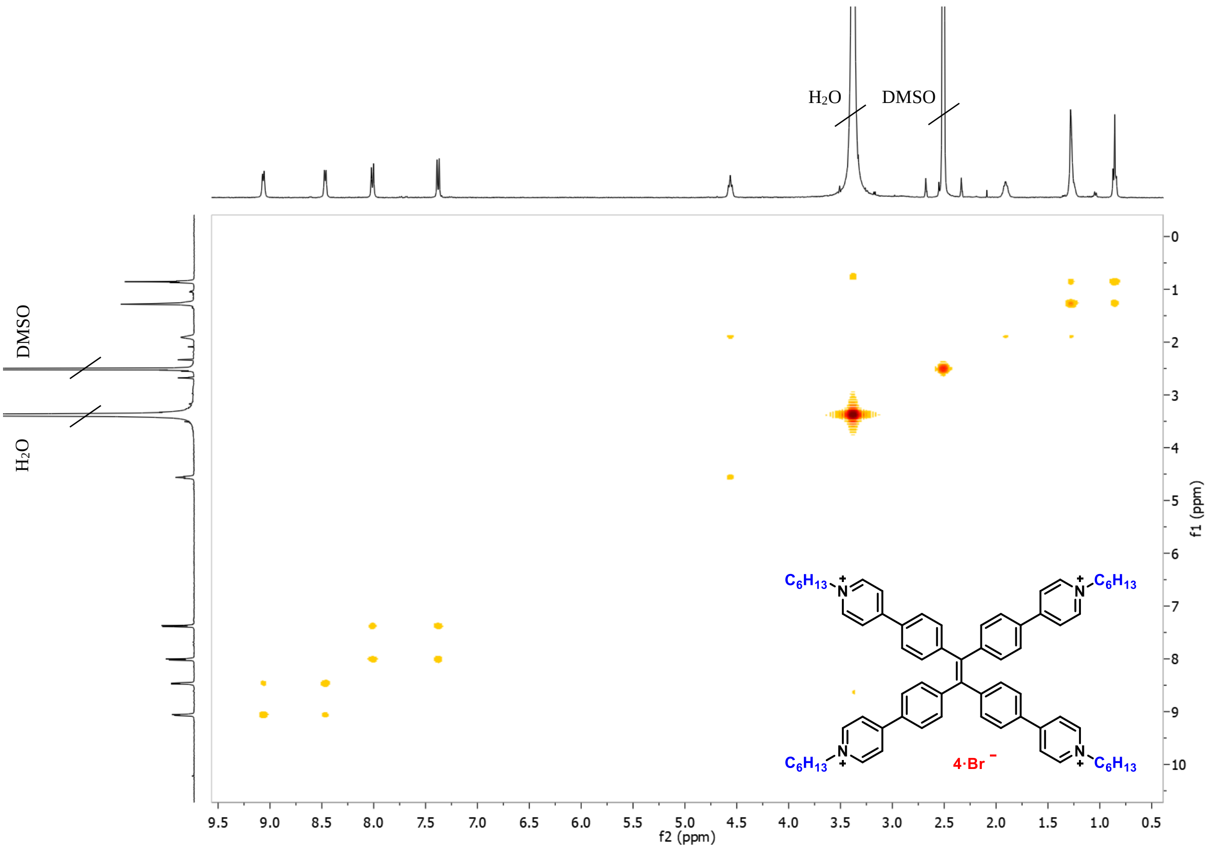

Fig. S21: COSY (DMSO-d6) spectrum of **6.Br**

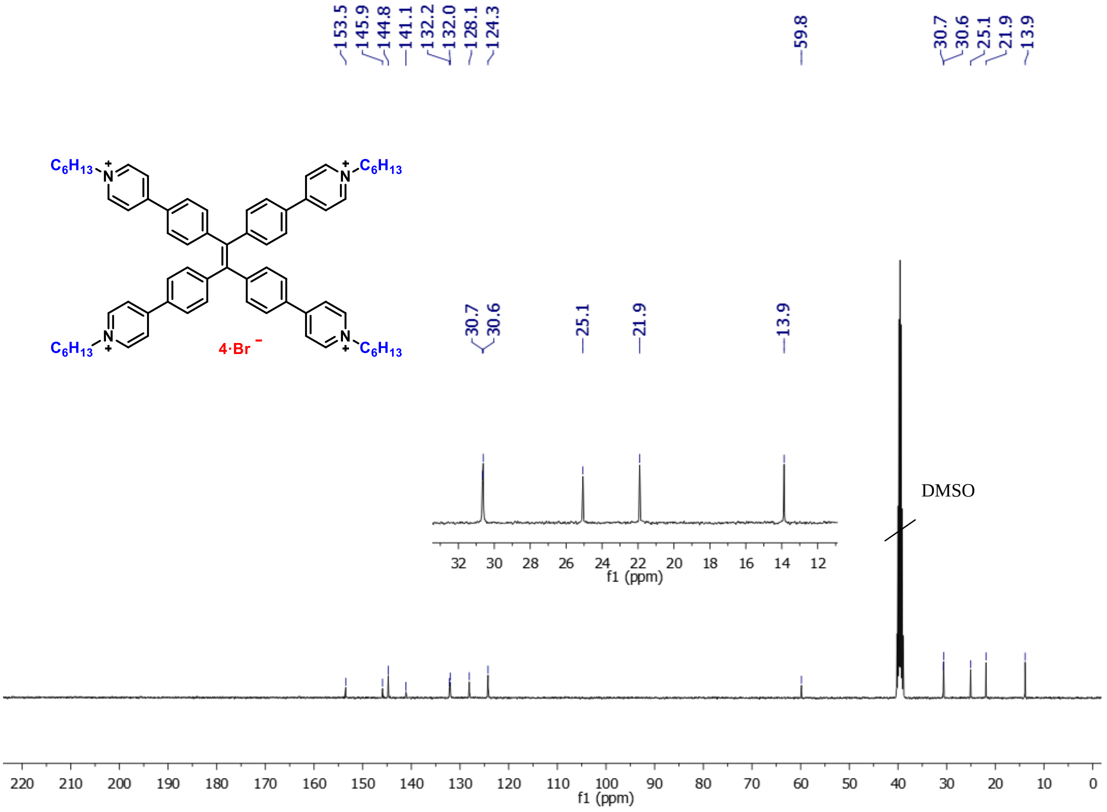

Fig. S22. ^{13}C-NMR (DMSO-d6, 100 MHz) spectrum of **6.Br.**

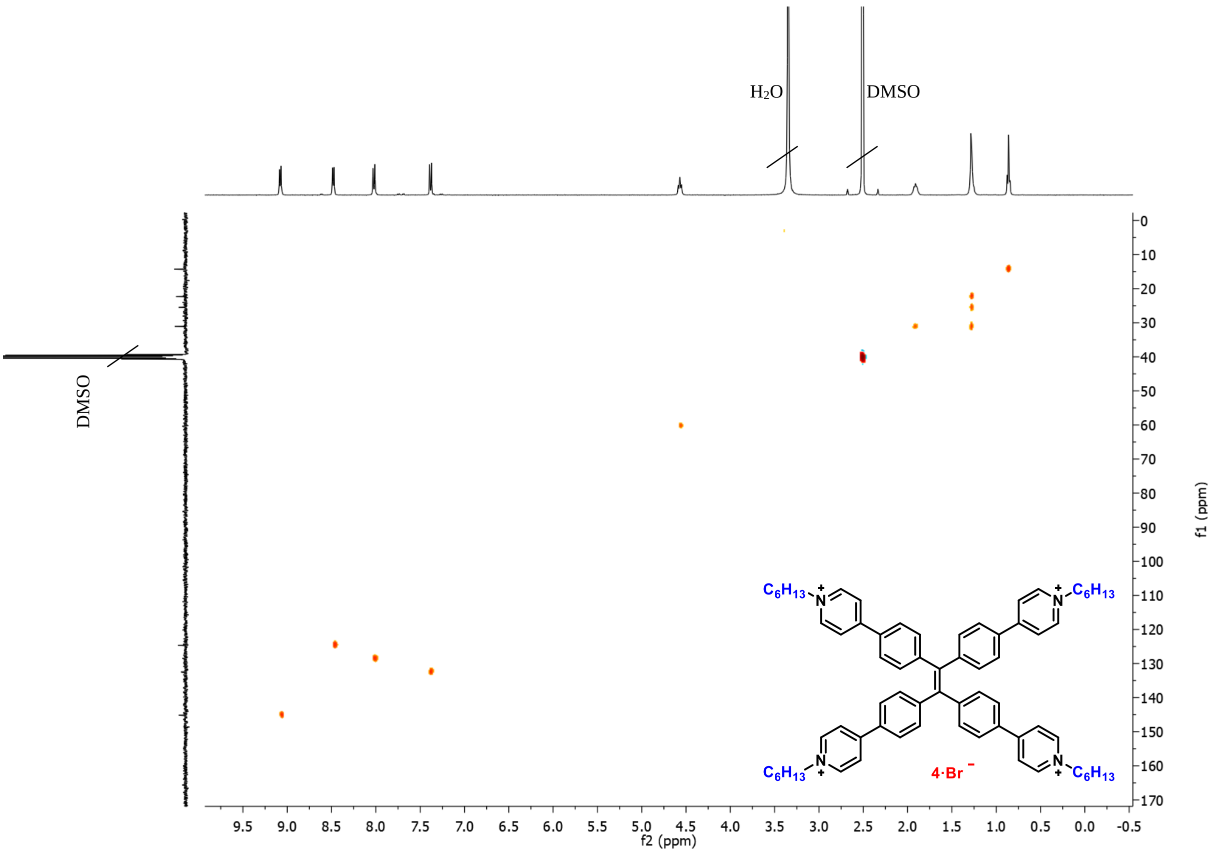

Fig. S23. HSQC (DMSO-d$_6$) spectrum of **6.Br**

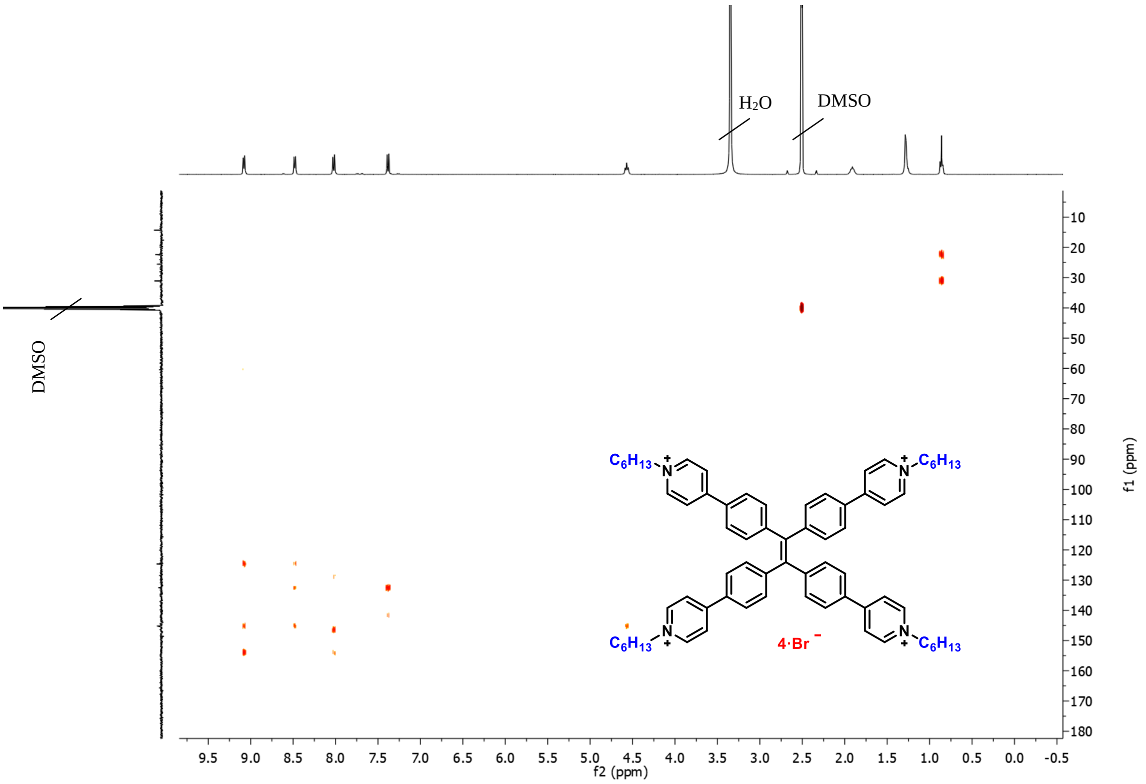

Fig. S24. HMBC (DMSO-d$_6$) spectrum of **6.Br.**

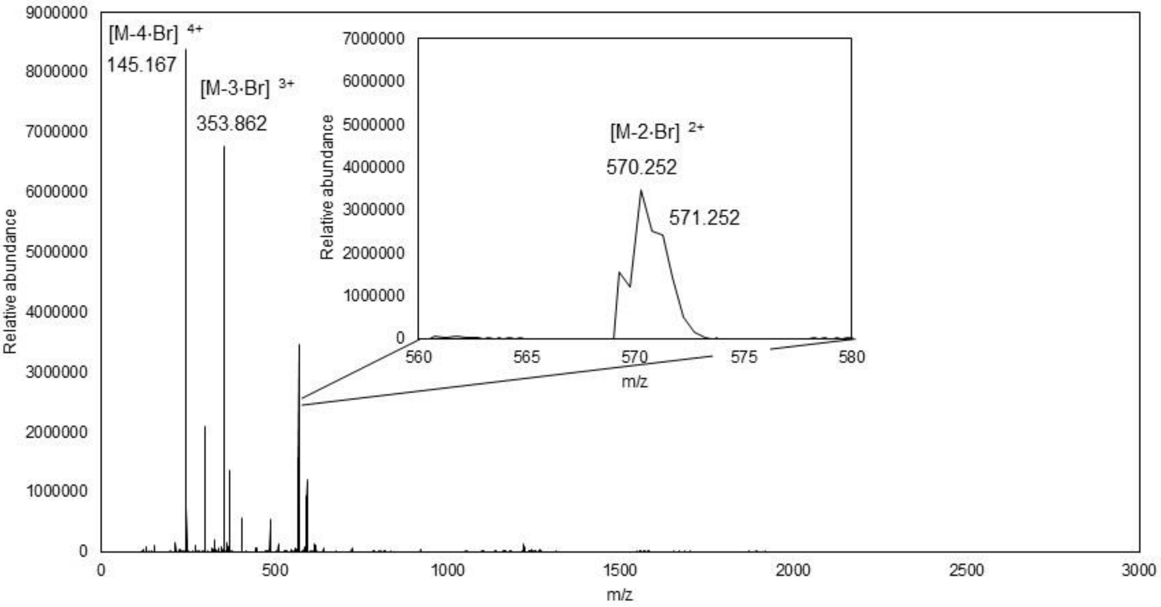

Fig. S25. ESI mass spectrum of **6.Br**

12.Br: 4,4',4'',4'''-(ethene-1,1,2,2-tetrayltetrakis(benzene-4,1-diyl))tetrakis(1-dodecylpyridin-1-ium) bromine with a 20 % yield. **^{1}H NMR** (400 MHz, DMSO) δ (ppm)= 9.04 (d, J = 6.5 Hz, 8H), 8.45 (d, J = 6.6 Hz, 8H), 7.98 (d, J = 8.4 Hz, 8H), 7.33 (d, J = 8.4 Hz, 8H), 4.52 (t, J = 6.4 Hz, 7H), 1.85 (d, J = 6.3 Hz, 8H), 1.19 (m, 71H), 0.80 (m, 14H). **^{13}C NMR** (400 MHz, DMSO-d_6): δ (ppm)= 154.0, 146.4, 145.2, 141.6, 132.7, 132.5, 128.6, 124.7, 60.4, 31.8, 31.2, 29.5, 29.4, 29.3, 29.2, 28.9, 25.9, 22.6, 14.5

HR-MS (ESI) m/z: $[M]^+$ calcd for $C_{94}H_{132}Br_4N_4$, 1632.72; found, 738.44 $[M\text{-}2{\cdot}Br]^+$

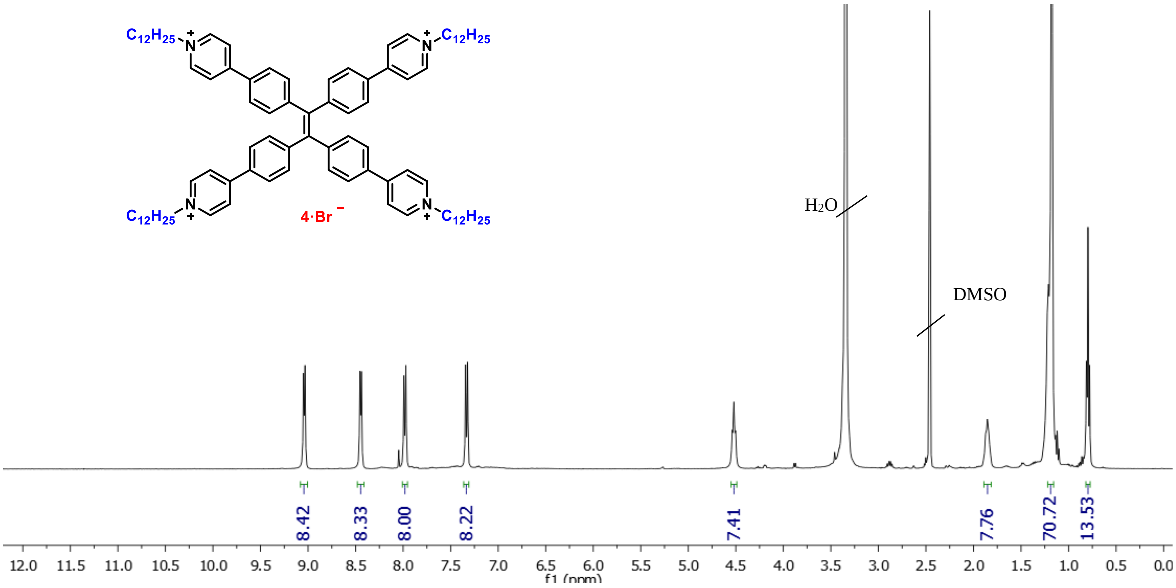

Fig. S26. ^{1}H-NMR (DMSO-d_6, 400 MHz) spectrum of **12.Br**

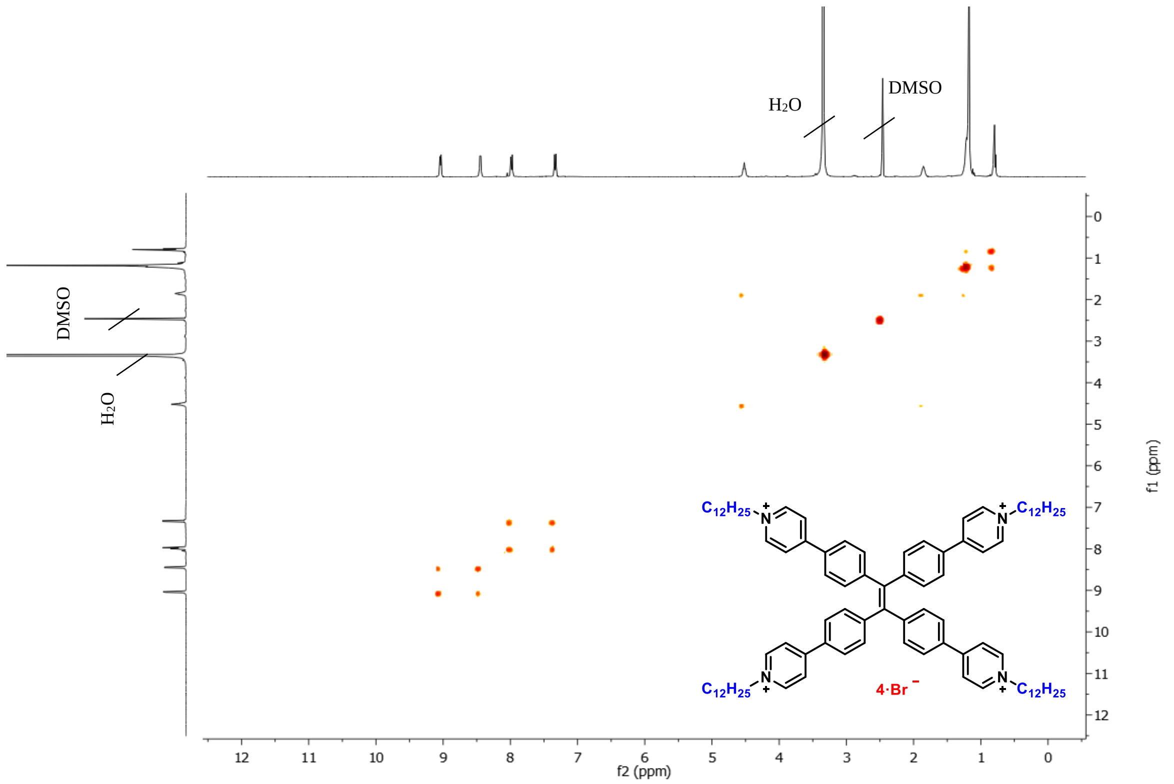

Fig. S27. COSY NMR (DMSO-d_6) spectrum of **12.Br** (DMSO-d_6)

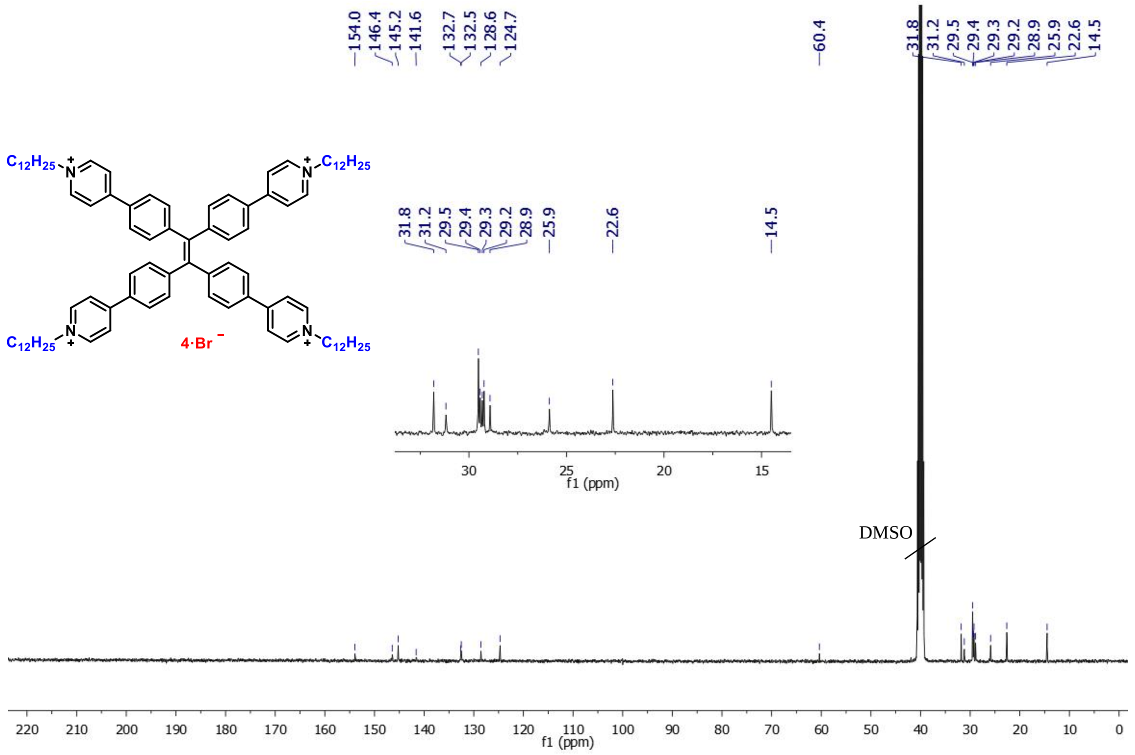

Fig. S28. ^{13}C-NMR (DMSO-d_6, 100MHz) spectrum of **12.Br**

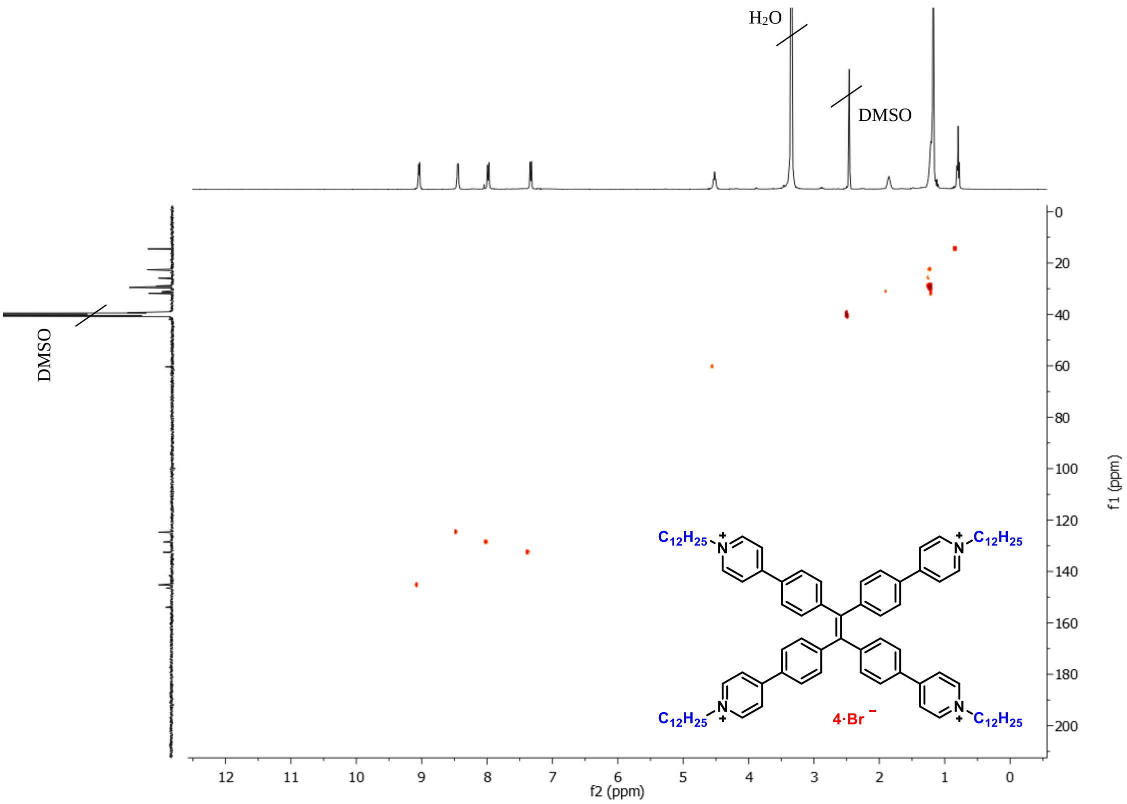

Fig. S29. HSQC (DMSO-d$_6$) spectrum of **12.Br.**

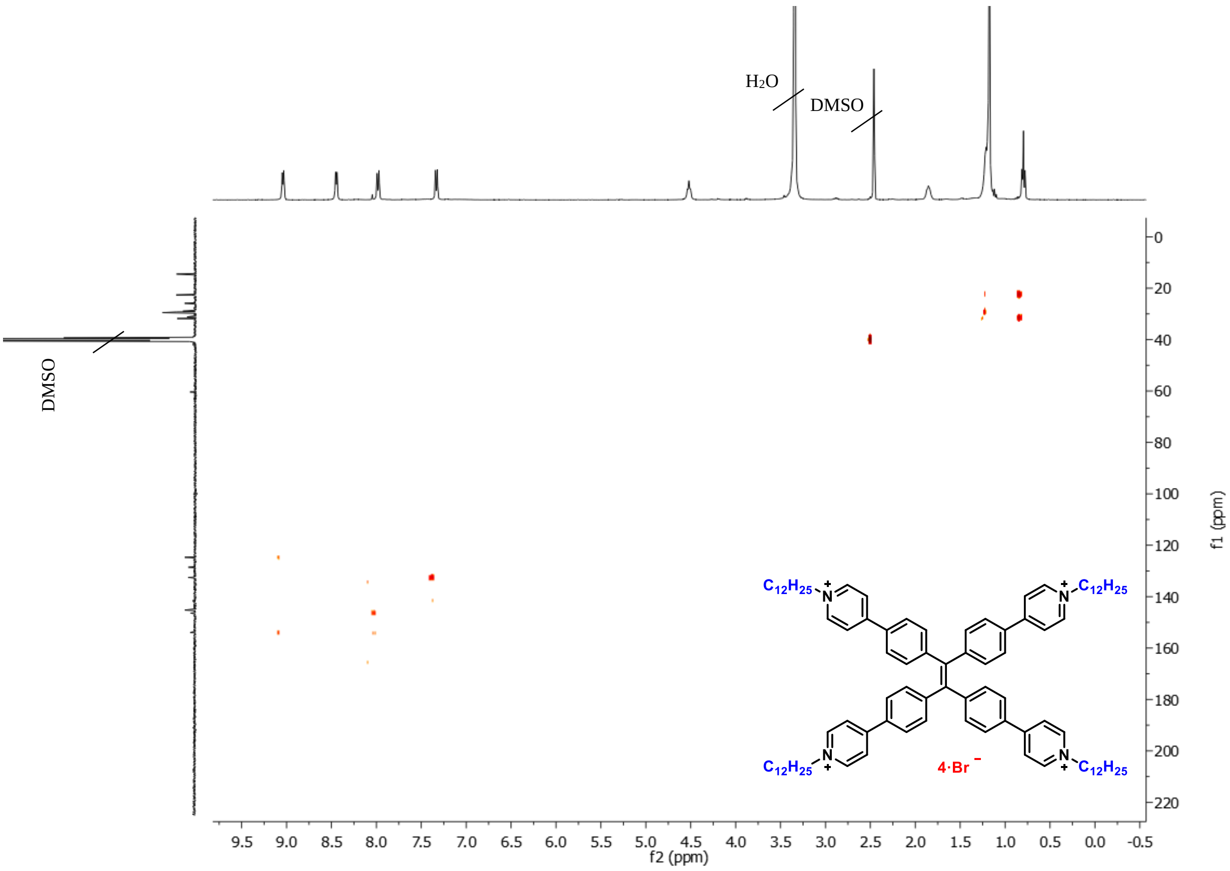

Fig. S30. HMBC (DMSO-d$_6$) spectrum of **12.Br.**

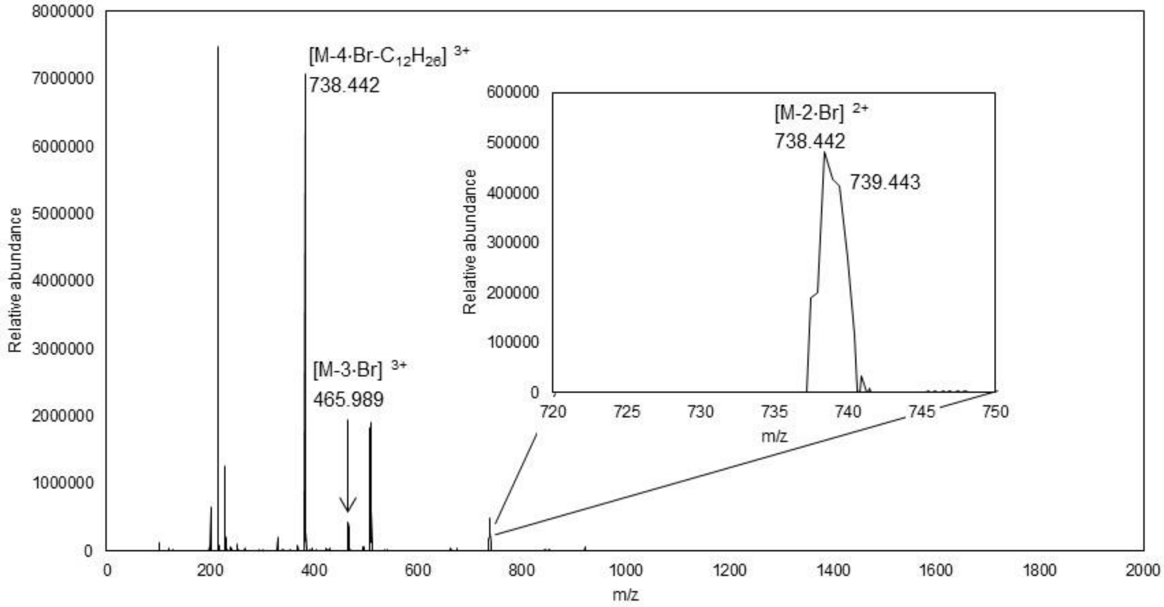

Fig. S31. ESI mass spectrum **12.Br**

Synthesis of PF6 TPPE-alkyl salts. Halide TPPE-alkyl salts were dissolved in a minimum amount of MeOH or water (for **1.I**). If any precipitation was observed the sample was centrifuge and only the supernatant was taken. In addition, a saturated solution of NH_4PF_6 in the same solvent was prepared. Directly, drop by drop NH_4PF_6 was added to the halide TPPE-alkyl salts dissolved. A solid precipitated immediately and it was separated by filtration.

1.PF6: 4,4',4'',4'''-(ethene-1,1,2,2-tetrayltetrakis(benzene-4,1-diyl))tetrakis(1-methylpyridin-1-ium) hexafluorophosphate with a 91 % yield. **^{1}H NMR** (400 MHz, DMSO-d_6): δ (ppm) = 8.96 (d, J = 7.0 Hz, 8H), 8.43 (d, J = 7.1 Hz, 8H), 7.99 (d, J = 8.6 Hz, 8H), 7.38 (d, J = 8.5 Hz, 8H), 4.31 (s, 12H). **^{13}C NMR** (400 MHz, DMSO-d_6): δ (ppm)= 153.6, 146.3, 146.0, 132.6, 132.5, 128.4, 124.2, 47.5

HR-MS (ESI) m/z: $[M]^+$ calcd for $C_{50}H_{44}F_{24}N_4P_4$, 1280.21; found, 1135.25 $[M\text{-}PF_6]^+$

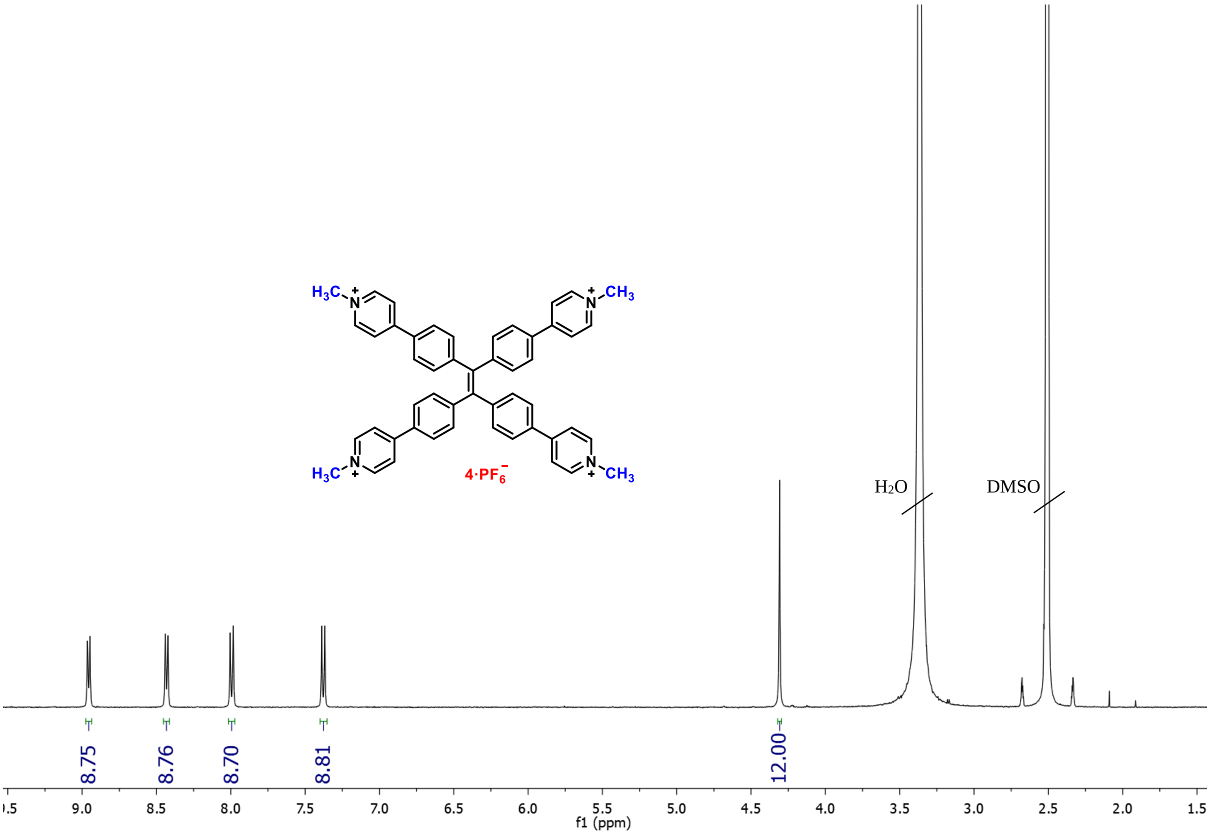

Fig. S32. ^{1}H-NMR (DMSO-d6, 400 MHz) spectrum of **$1.PF_6$**

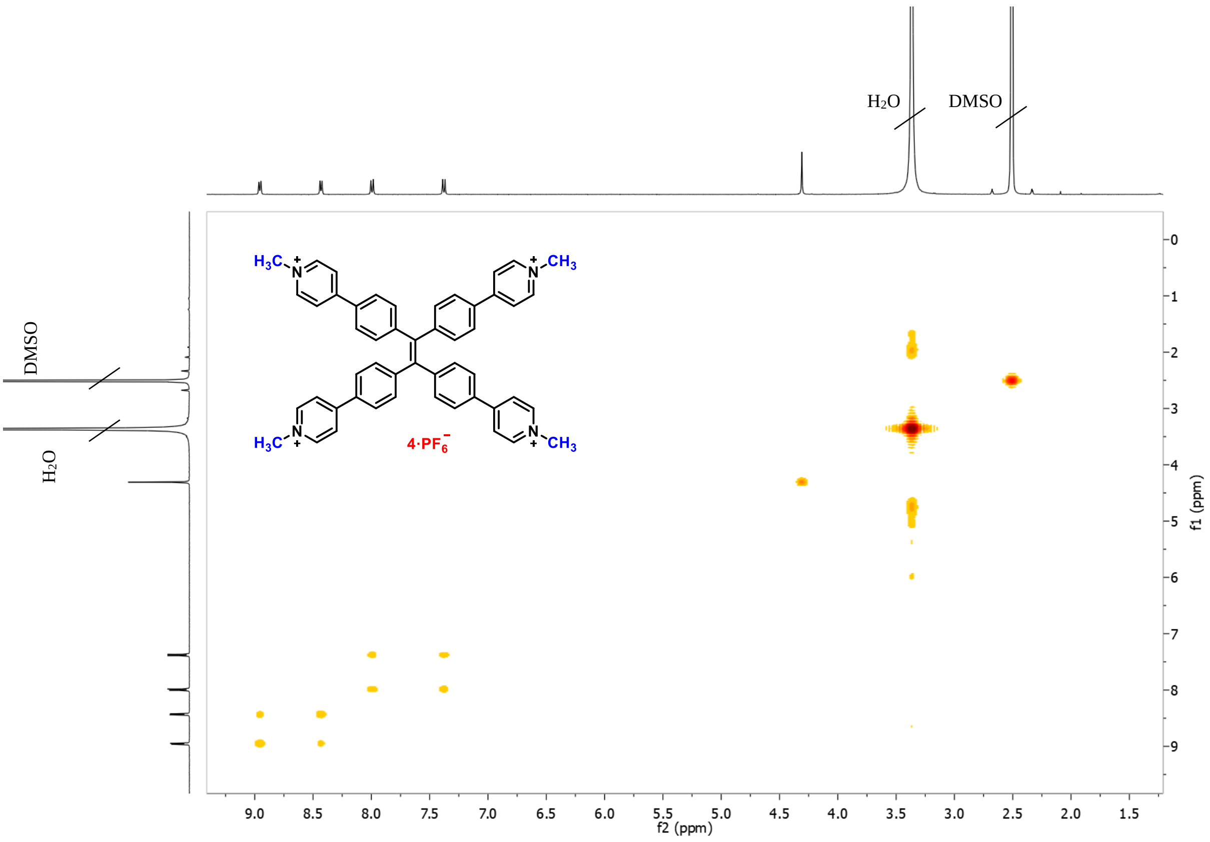

Fig. S33. COSY NMR (DMSO-d$_6$) spectrum of **1.PF$_6$**

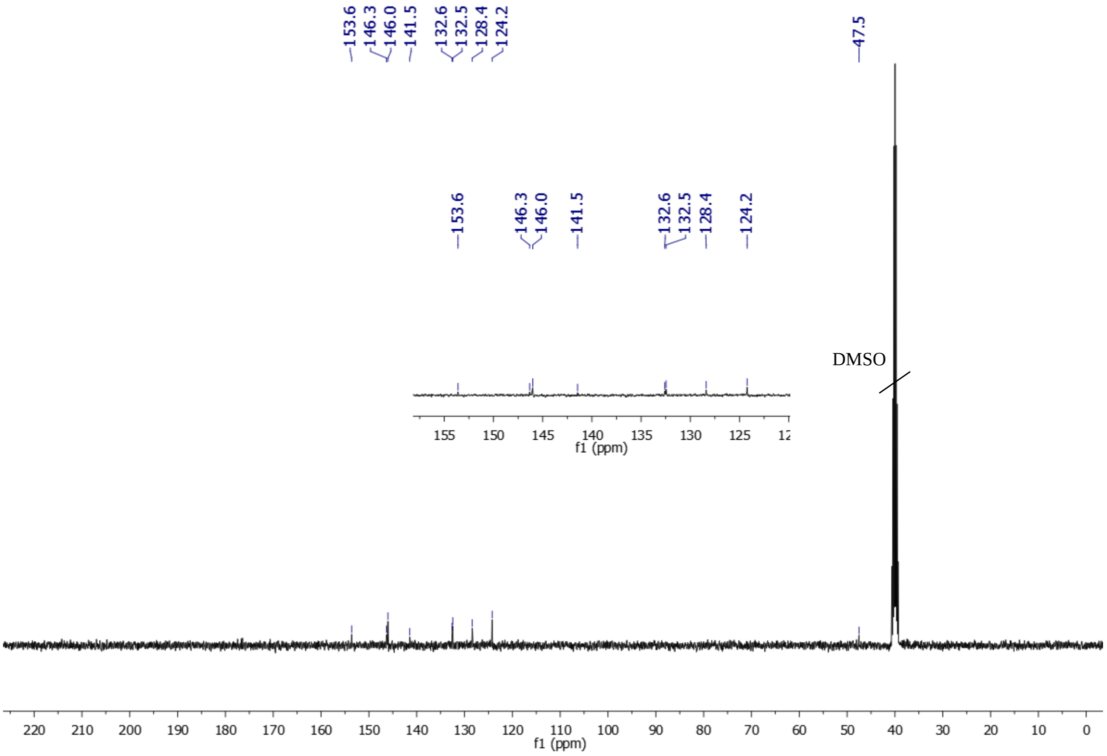

Fig. S34. ^{13}C-NMR (DMSO-d$_6$, 100 MHz) spectrum of **1.PF$_6$**

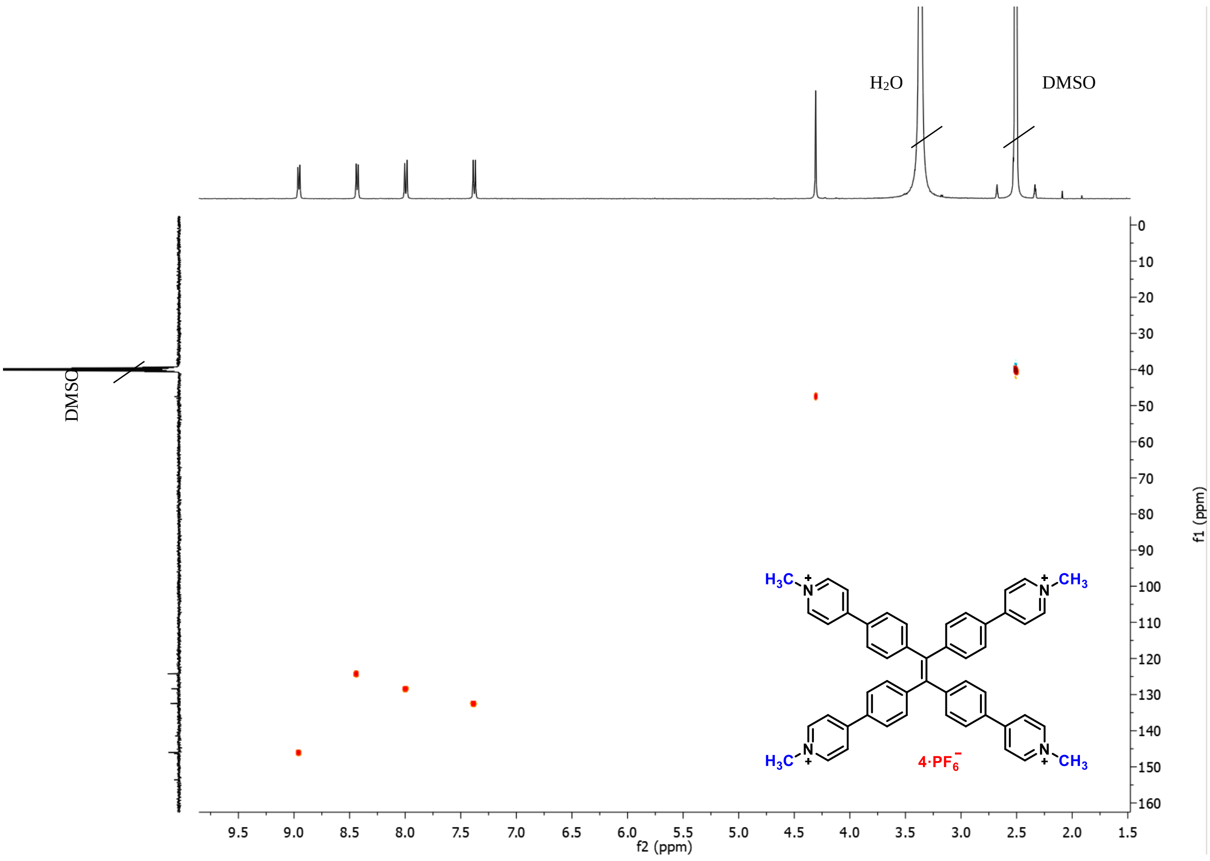

Fig. S35. HSQC (DMSO-d$_6$) spectrum of **1.PF$_6$**

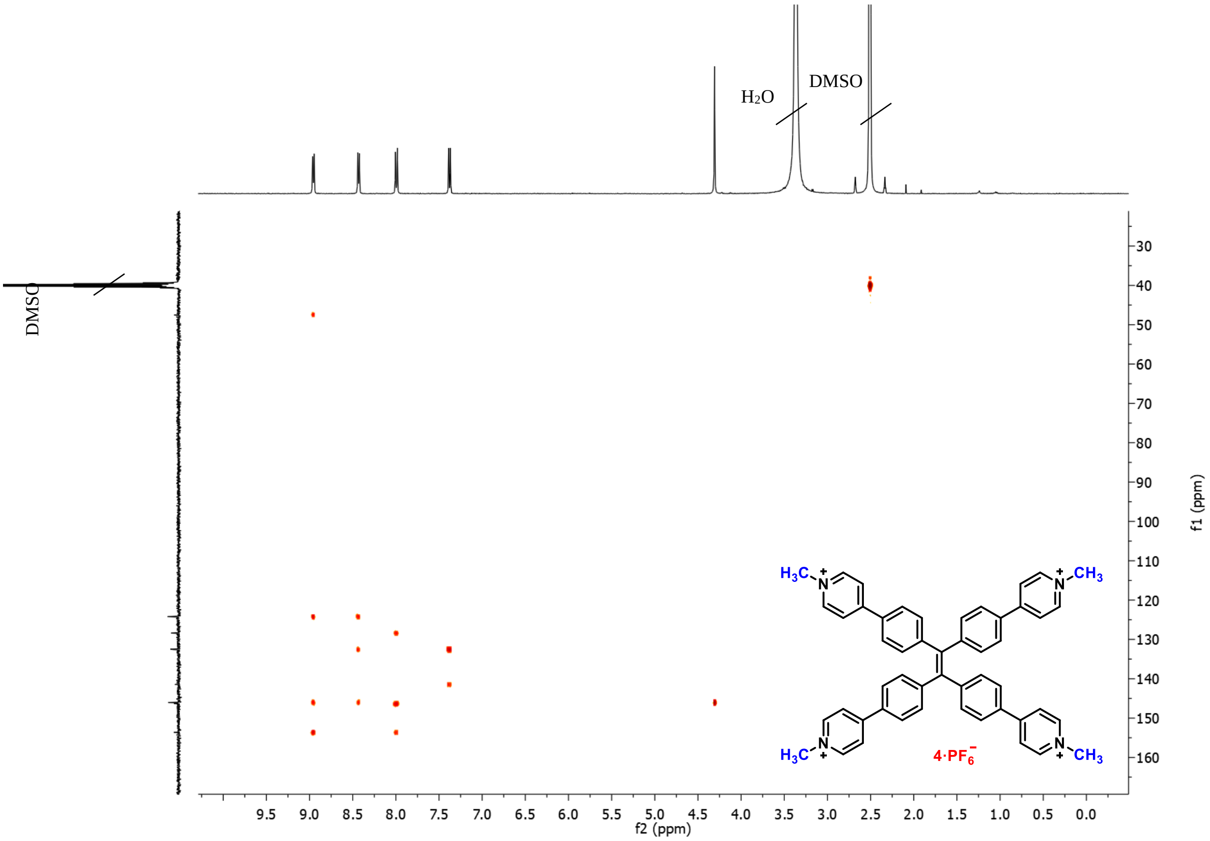

Fig. S36. HMBC (DMSO-d$_6$) spectrum of **1.PF$_6$**

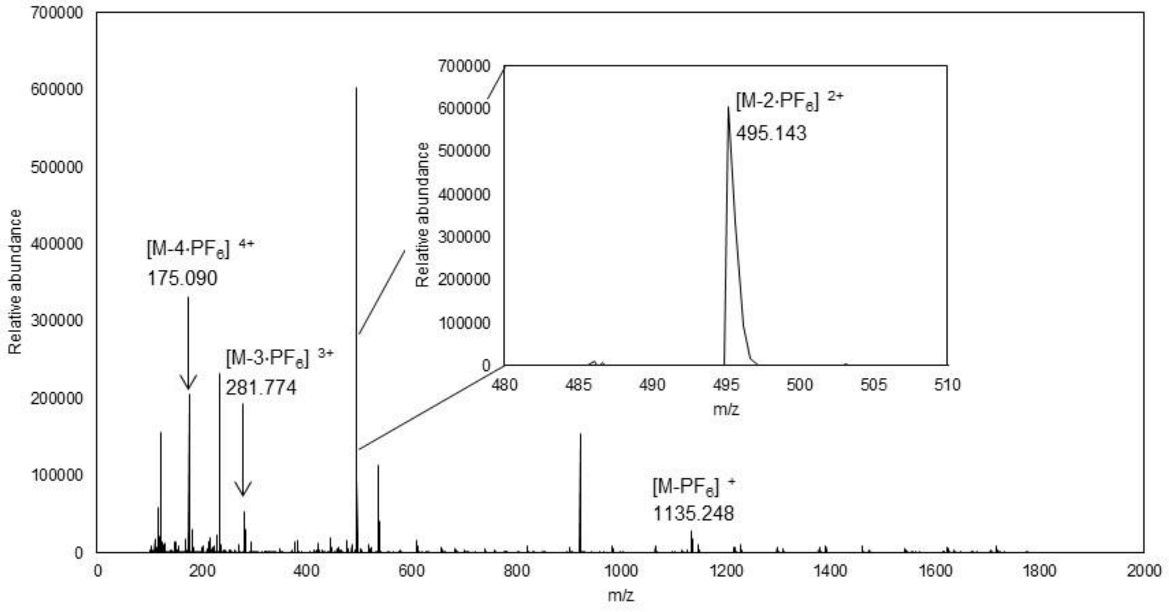

Fig. S37. ESI mass spectrum **1.PF$_6$**

2.PF6: 4,4',4'',4'''-(ethene-1,1,2,2-tetrayltetrakis(benzene-4,1-diyl))tetrakis(1-ethylpyridin-1-ium) hexafluorophosphate with a 95 % yield. **^{1}H NMR** (400 MHz, DMSO-d$_6$): δ (ppm) = 9.07 (d, J = 7.0 Hz, 8H), 8.46 (d, J = 7.0 Hz, 8H), 8.00 (d, J = 8.5 Hz, 8H), 7.38 (d, J = 8.4 Hz, 8H), 4.60 (q, J = 7.2 Hz, 8H), 1.54 (t, J = 7.3 Hz, 12H). **^{13}C NMR** (400 MHz, DMSO-d$_6$): δ (ppm)= 154.0, 146.4, 145.0, 132.7, 132.5, 128.5, 124.7, 56.0, 16.8

HR-MS (ESI) m/z: $[M]^+$ calcd for $C_{54}H_{52}F_{24}N_4P_4$, 1336.27; found, 1191.31 $[M-PF_6]^+$

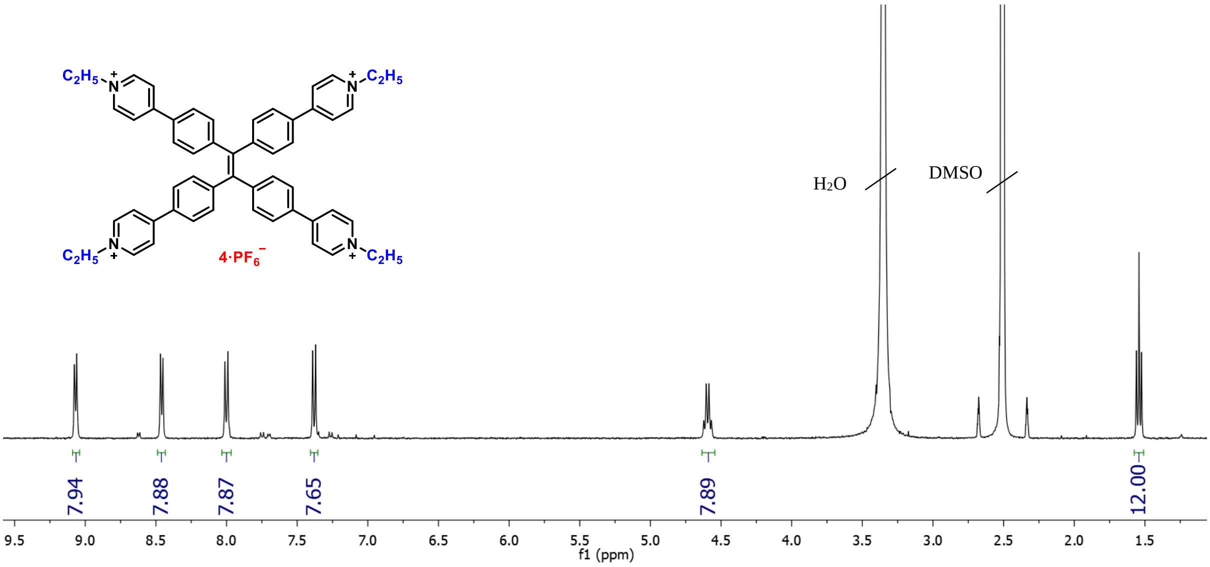

Fig. S38. ^{1}H-NMR (DMSO-d6, 400 MHz) spectrum of **2.PF$_6$**

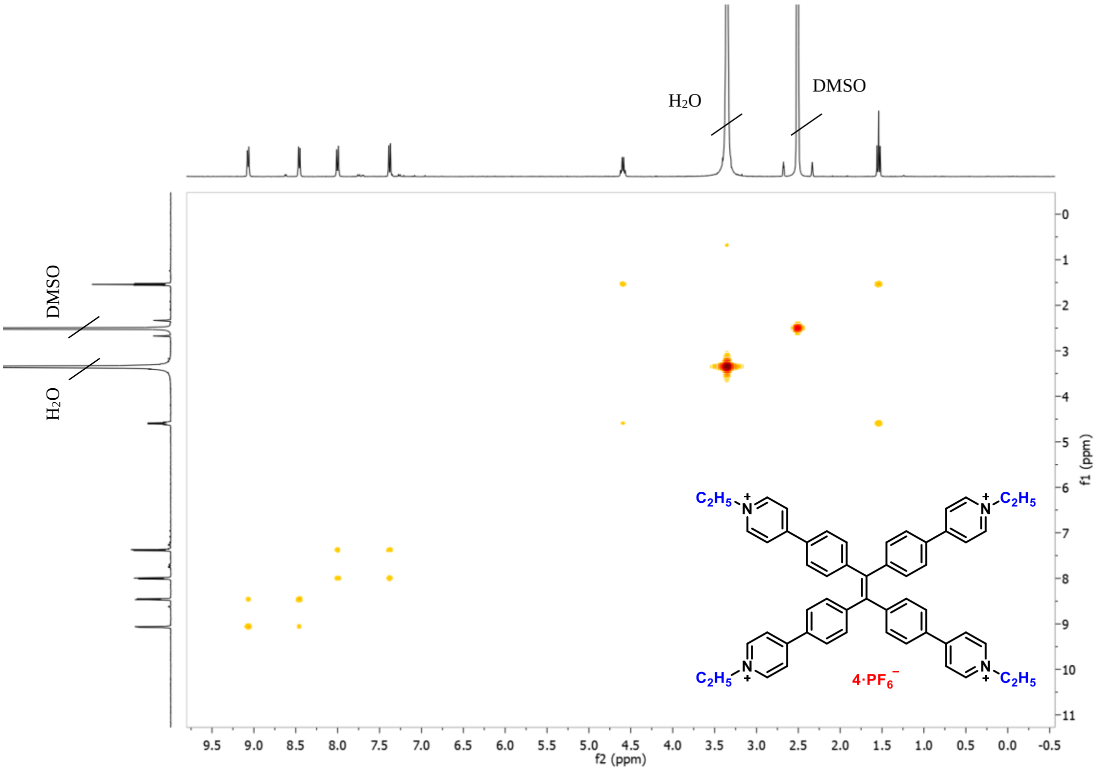

Fig. S39. COSY NMR (DMSO-d$_6$) spectrum of **2.PF$_6$**

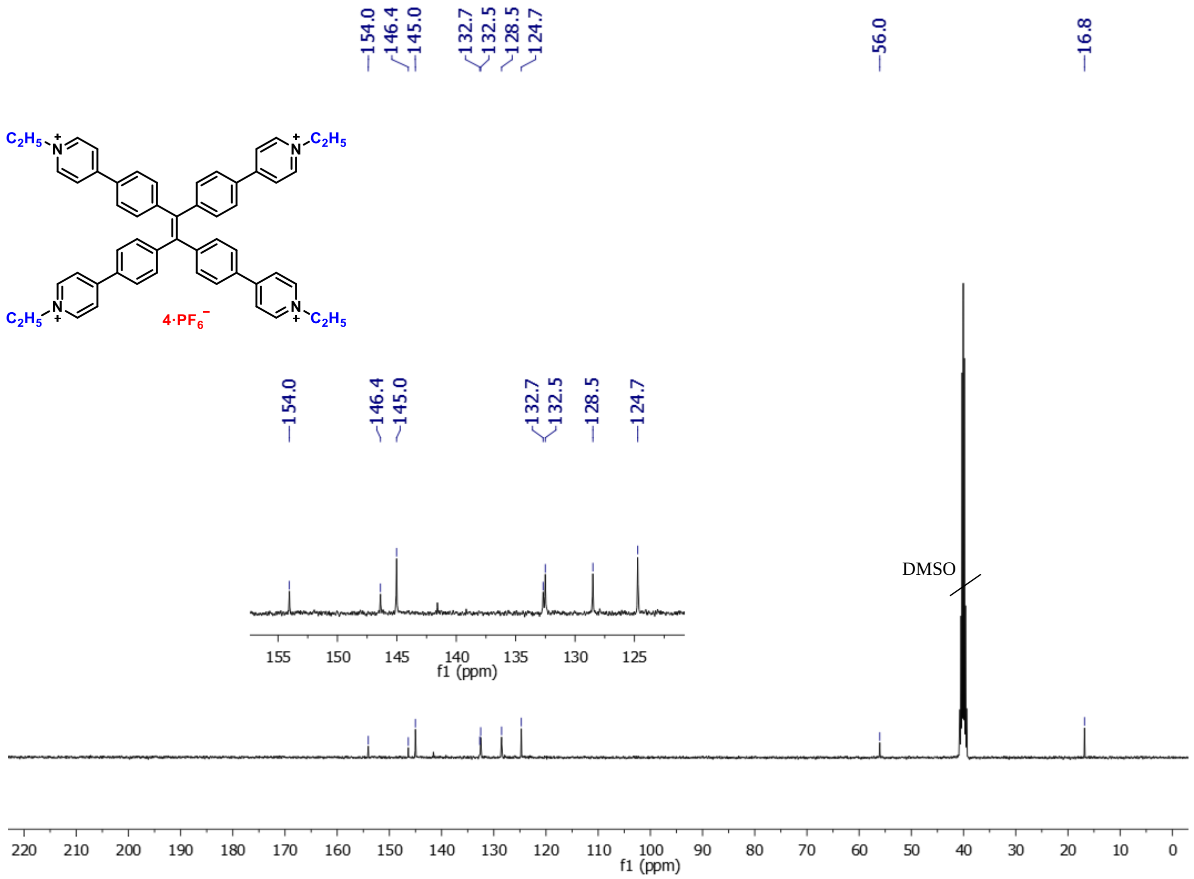

Fig. S40. ^{13}C-NMR (DMSO-d$_6$, 100 MHz) spectrum of **2.PF$_6$**

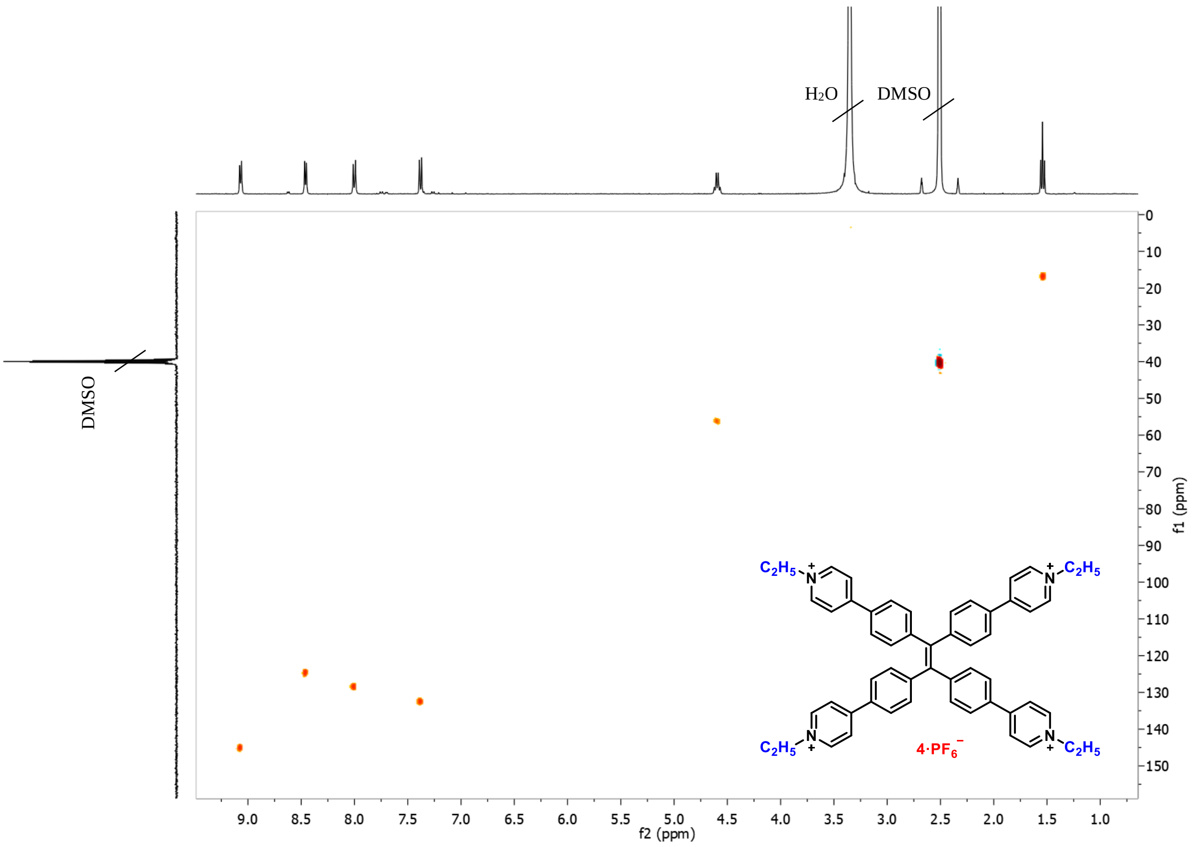

Fig. S41. HSQC (DMSO-d$_6$) spectrum of **2.PF$_6$**

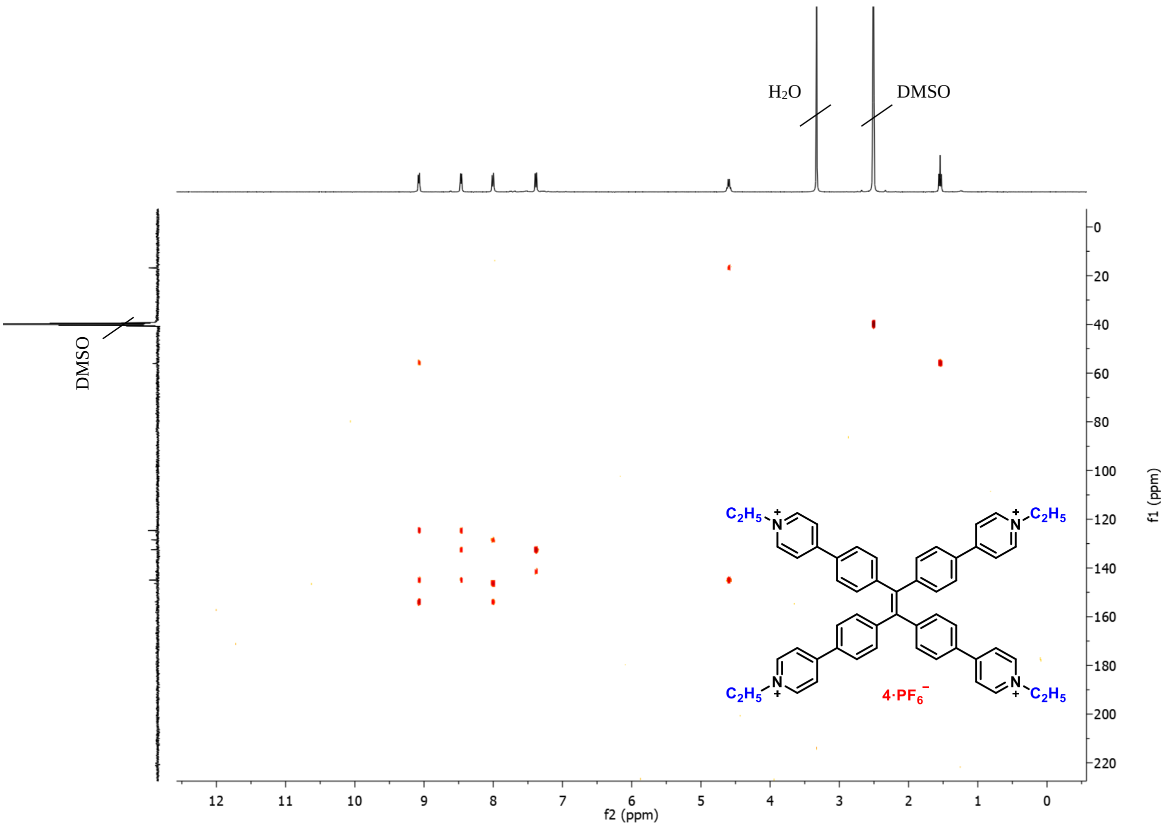

Fig. S42. HMBC (DMSO-d$_6$) spectrum of **2.PF$_6$**

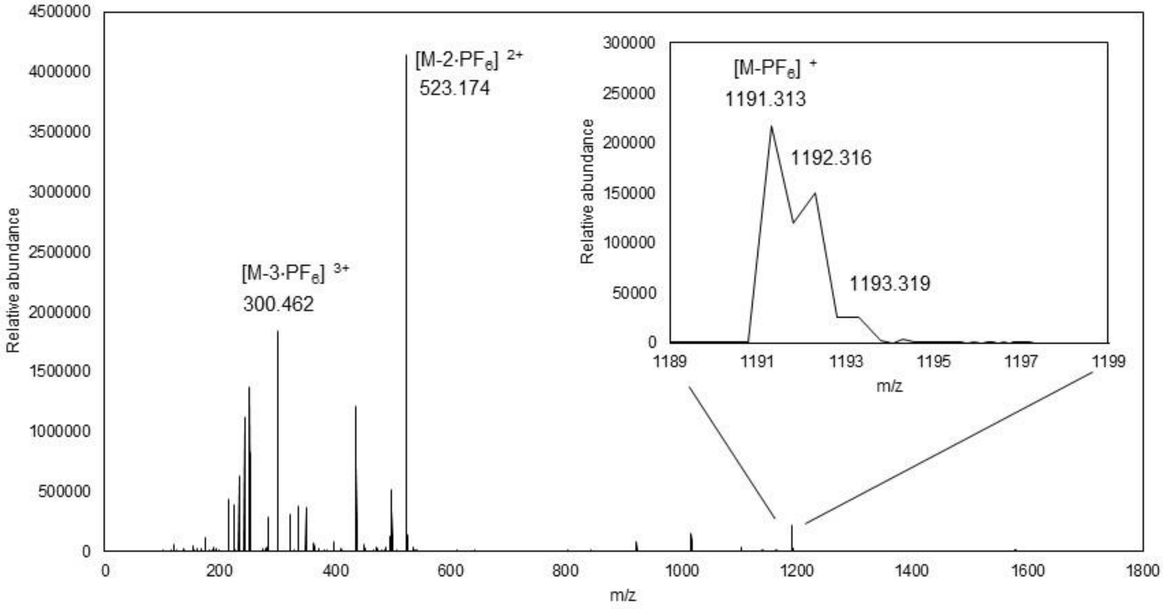

Fig. S43. ESI mass spectrum **2.PF$_6$**

4.PF6: 4,4',4'',4'''-(ethene-1,1,2,2-tetrayltetrakis(benzene-4,1-diyl))tetrakis(1-buthylpyridin-1-ium) hexafluorophosphate with a 80 % yield ^{1}**H NMR** (400 MHz, DMSO-d_6): δ (ppm) =9.06 (d, J = 7.0 Hz, 8H), 8.46 (d, J = 7.1 Hz, 8H), 8.00 (d, J = 8.5 Hz, 8H), 7.38 (d, J = 8.5 Hz, 8H), 4.56 (t, J = 7.1 Hz, 8H), 1.93 – 1.85 (m, 8H), 1.29 (dt, J = 14.8, 7.4 Hz, 9H), 0.92 (t, J = 7.4 Hz, 12H). . ^{13}**C NMR** (400 MHz, DMSO-d_6): δ (ppm)= 154.1, 146.4, 145.2, 141.6, 132.7, 132.5, 128.5, 124.7, 60.2, 33.1, 19.3, 13.8

HR-MS (ESI) m/z: $[M]^+$ calcd for $C_{62}H_{68}F_{24}N_4P_4$, 1448.40; found, 579.24 $[M\text{-}2{\cdot}PF_6]^+$

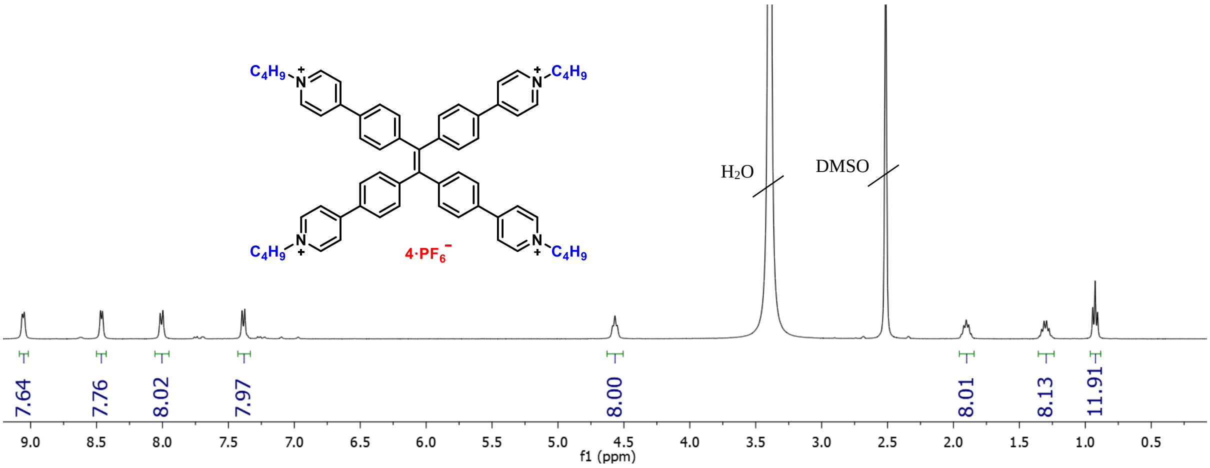

Fig. S44. ^{1}H-NMR (DMSO-d6, 400 MHz) spectrum of **4.PF$_6$**

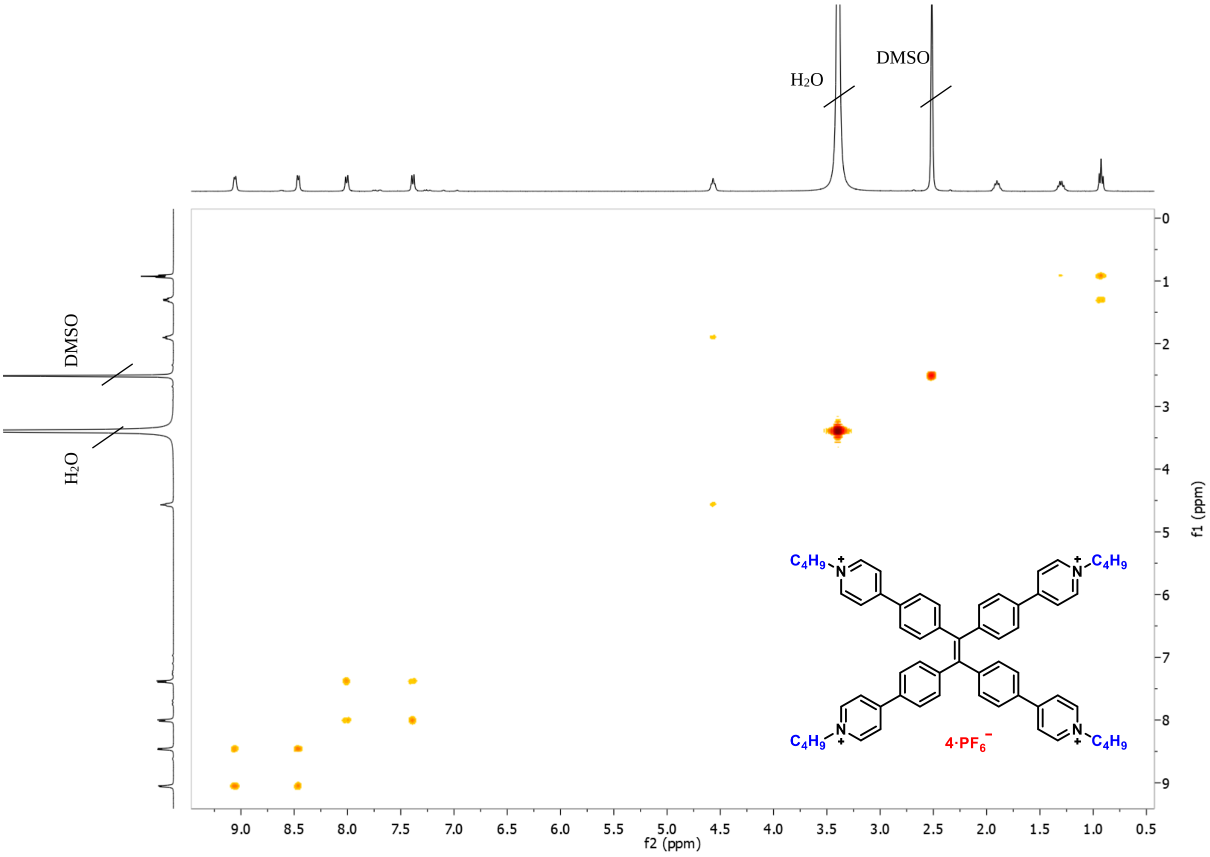

Fig. S45. COSY NMR (DMSO-d_6) spectrum of **4.PF$_6$**

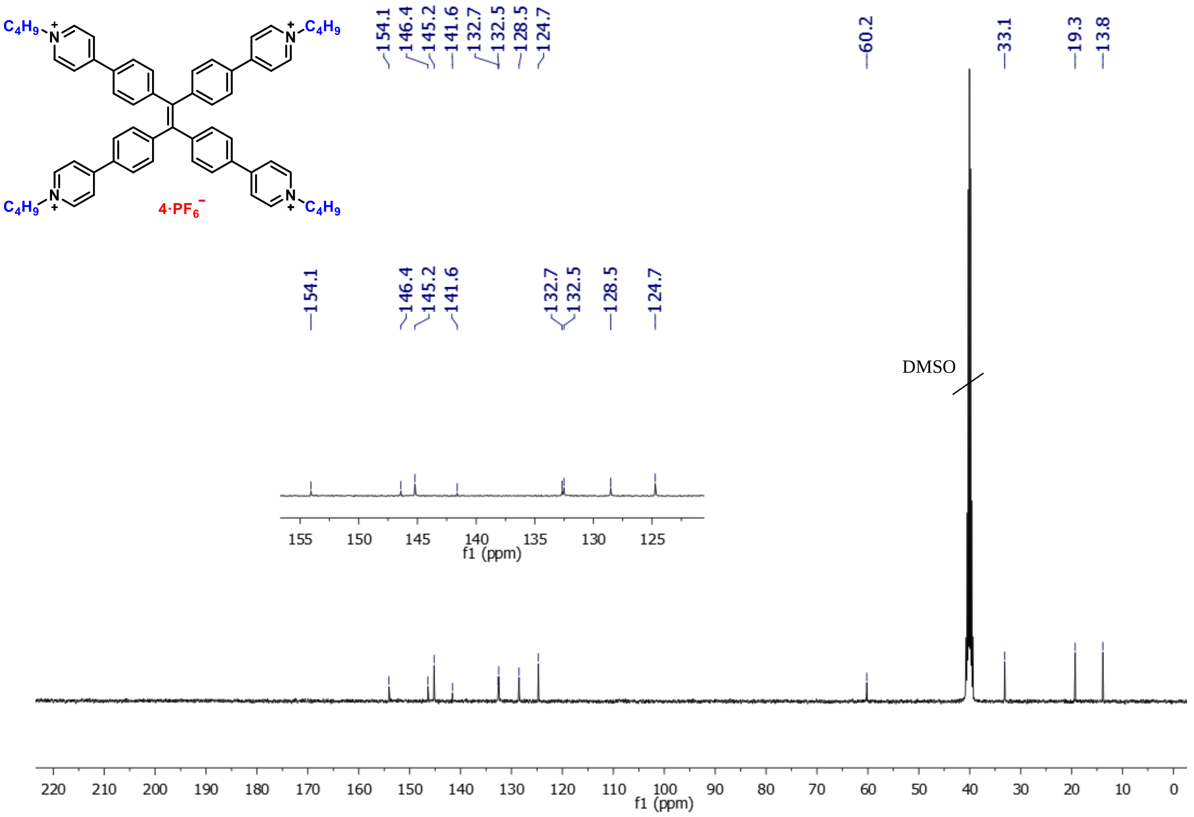

Fig. S46. ^{13}C-NMR (DMSO-d$_6$) spectrum of **4.PF$_6$**

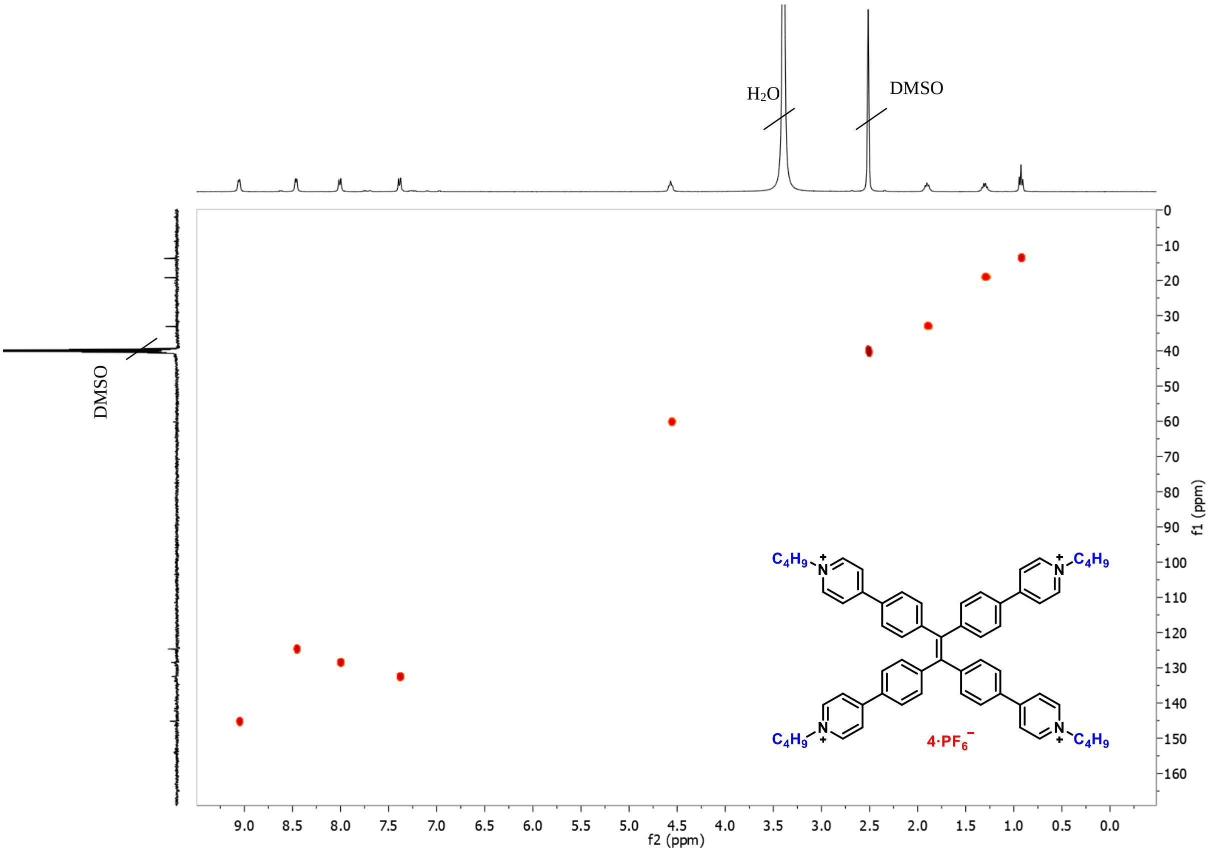

Fig. S47. HSQC (DMSO-d$_6$) spectrum of **4.PF$_6$**

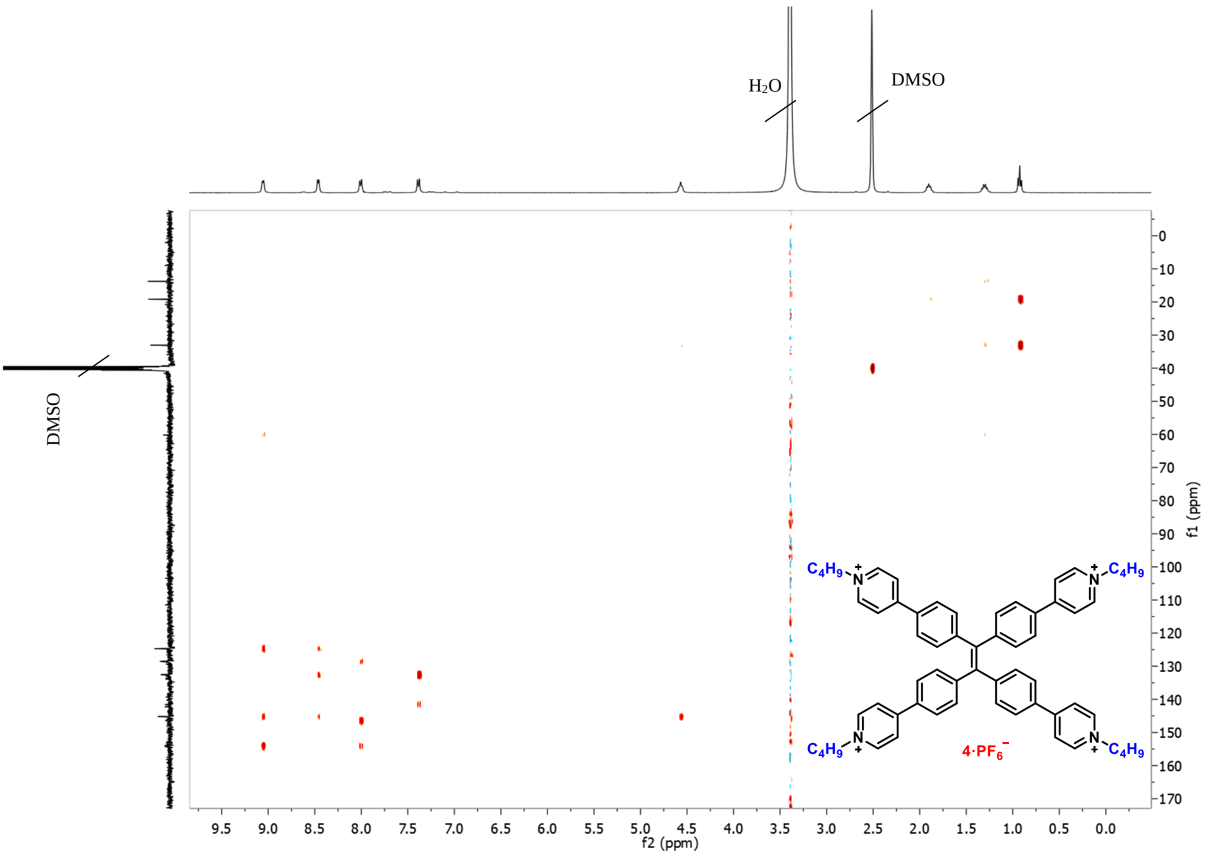

Fig. S48. HMBC (DMSO-d$_6$) spectrum of **4.PF$_6$**

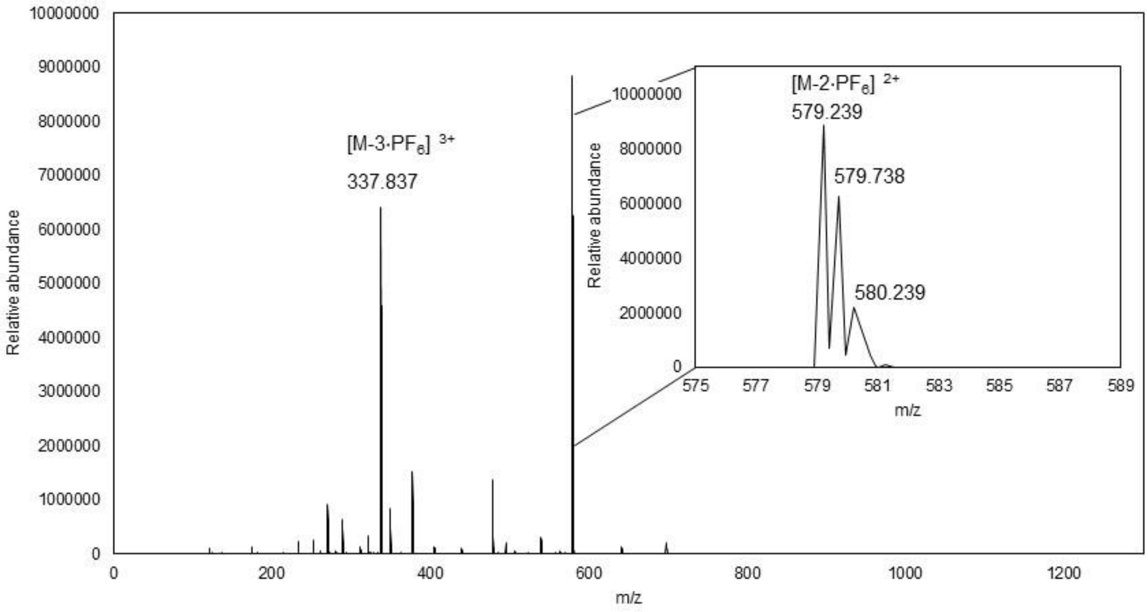

Fig. S49. ESI mass spectrum **4.PF$_6$**

6.PF6: 4,4',4'',4'''-(ethene-1,1,2,2-tetrayltetrakis(benzene-4,1-diyl))tetrakis(1-hexylpyridin-1-ium) hexafluorophosphate with 94 % yield. **^{1}H NMR** (400 MHz, DMSO-d$_6$): δ (ppm) = 9.06 (d, J = 6.9 Hz, 8H), 8.46 (d, J = 7.0 Hz, 8H), 8.01 (d, J = 8.5 Hz, 8H), 7.38 (d, J = 8.5 Hz, 8H), 4.56 (t, J = 7.2 Hz, 8H), 1.91 (s, 8H), 1.28 (s, 26H), 0.86 (t, J = 6.7 Hz, 13H). **^{13}C NMR** (400 MHz, DMSO-d$_6$): δ (ppm)= 150.0, 146.4, 145.2, 141.6, 132.7, 132.5, 128.5, 124.7, 60.5, 31.1, 31.1, 25.6, 22.4, 14.3

HR-MS (ESI) m/z: [M]$^+$ calcd for $C_{70}H_{84}F_{24}N_4P_4$, 1416.56; found, [M-PF$_6$]$^+$

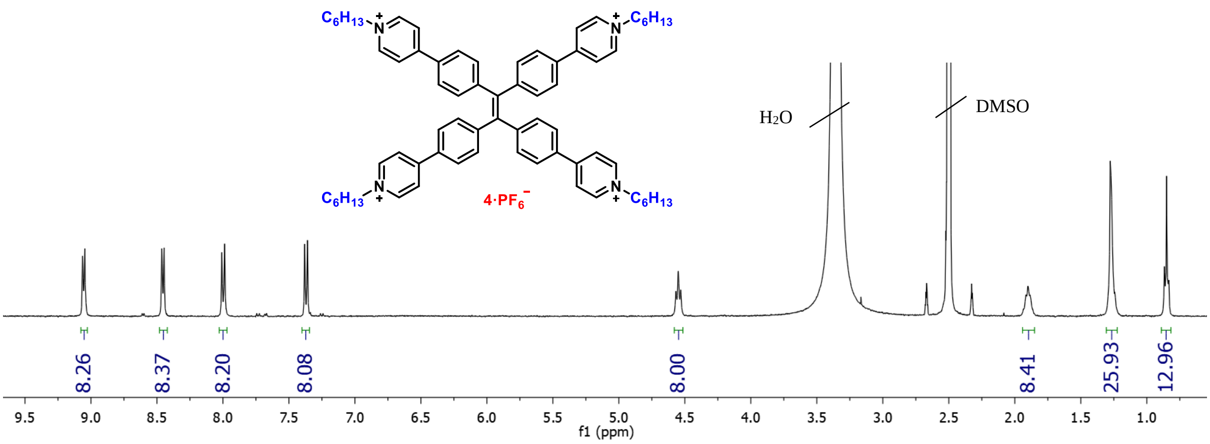

Fig. S50. ^{1}H-NMR (DMSO-d6, 400 MHz) spectrum of **6.PF$_6$**

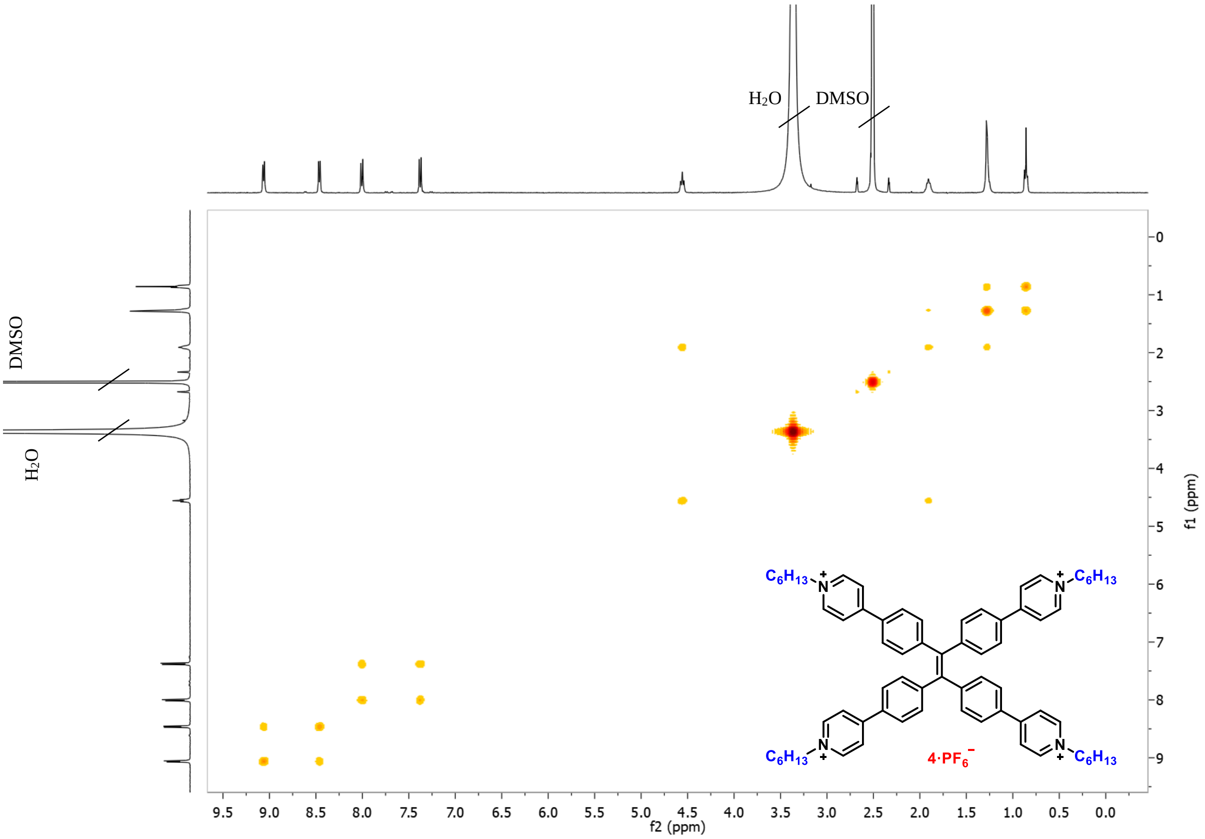

Fig. S51. COSY NMR (DMSO-d$_6$) spectrum of **6.PF$_6$**

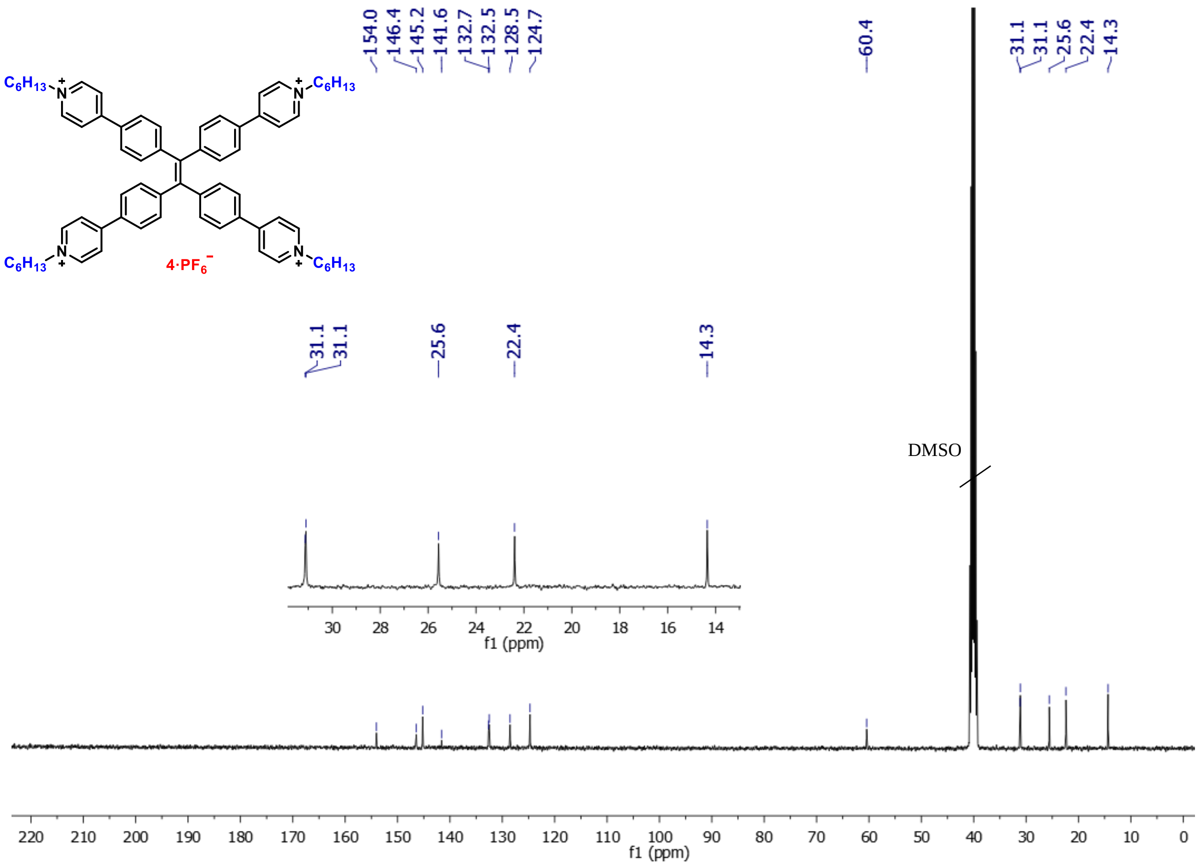

Fig. S52. ^{13}C-NMR (DMSO-d$_6$, 100 MHz) spectrum of **6.PF$_6$**

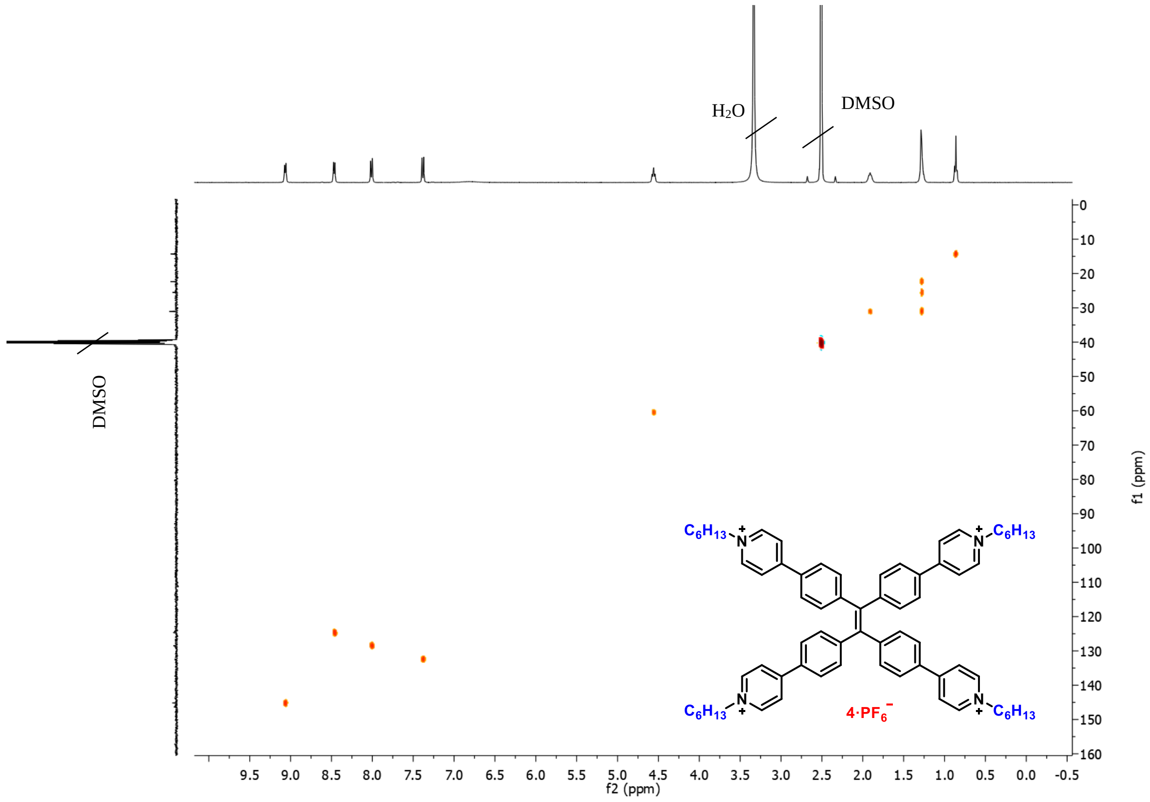

Fig. S53. HSQC (DMSO-d$_6$) spectrum of **6.PF$_6$**

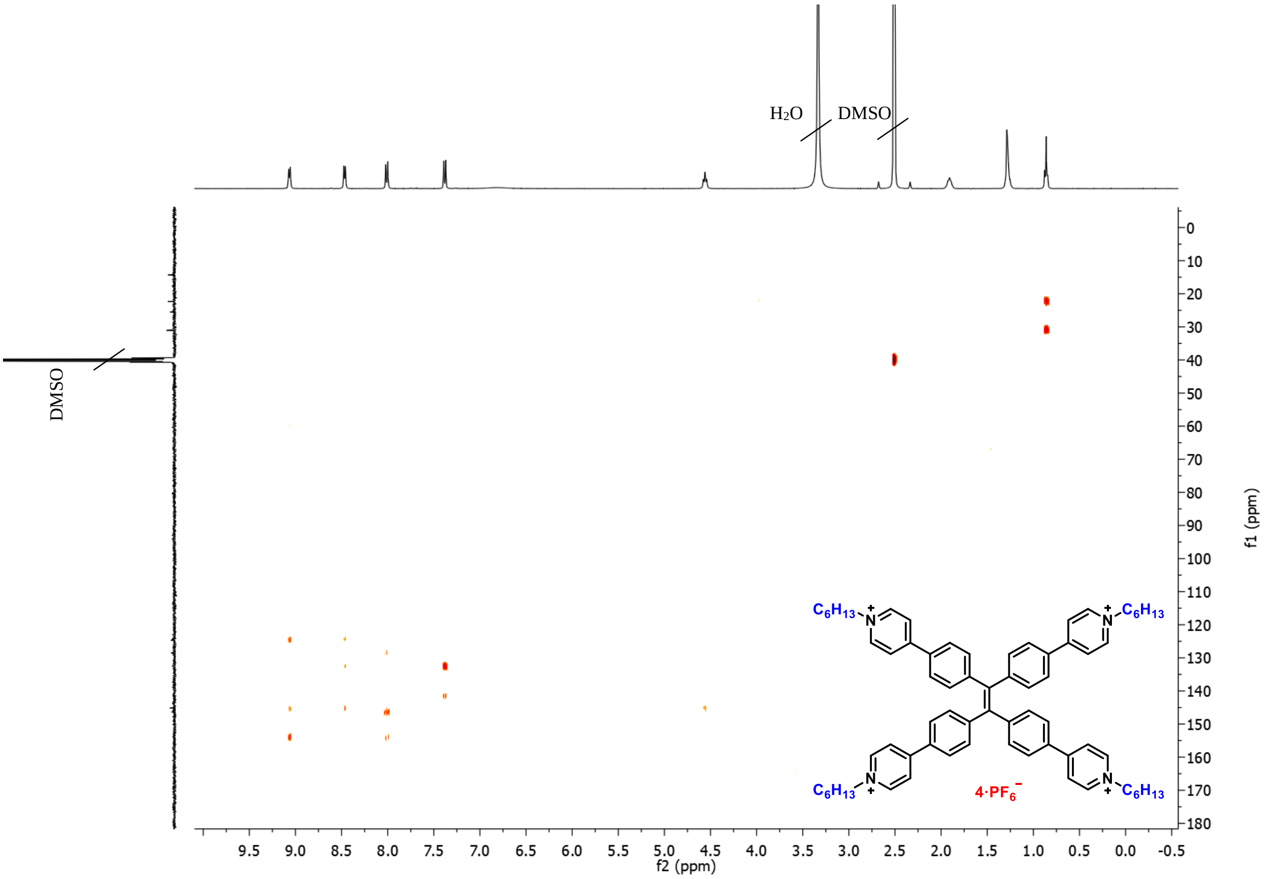

Fig. S54. HMBC (DMSO-d$_6$) spectrum of **6.PF$_6$**

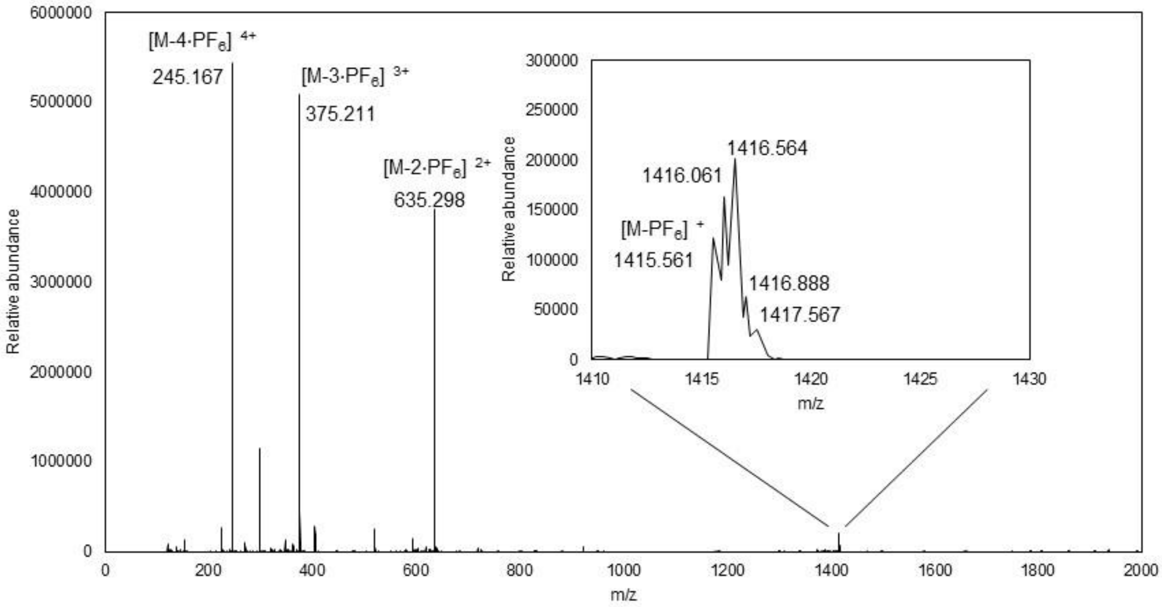

Fig. S55. ESI mass spectrum of **6.PF$_6$**

12.PF6: 4,4',4'',4'''-(ethene-1,1,2,2-tetrayltetrakis(benzene-4,1-diyl))tetrakis(1-dodecylpyridin-1-ium) hexafluorophosphate with 85% yield. ^{1}H NMR (400 MHz, DMSO) δ (ppm) = 9.06 (d, J = 6.8 Hz, 1H), 8.46 (d, J = 6.9 Hz, 1H), 8.00 (d, J = 8.5 Hz, 1H), 7.38 (d, J = 8.4 Hz, 1H), 4.55 (t, J = 7.0 Hz, 8H), 1.91 (d, J = 6.5 Hz, 1H), 1.25 (d, J = 14.1 Hz, 10H), 0.85 (t, J = 6.9 Hz, 2H). **^{13}C NMR** (400 MHz, DMSO-d_6): δ (ppm)= 154.0, 146.3, 145.1, 141.6, 132.6, 132.4, 128.4, 124.6, 60.4, 31.7, 31.1, 29.4, 29.3, 29.2, 29.1, 28.8, 25.8, 22.5, 14.4

HR-MS (ESI) m/z: $[M]^+$ calcd for $C_{94}H_{132}F_{24}N_4P_4$, 803.90; found, $[M\text{-}2{\cdot}PF_6]^+$

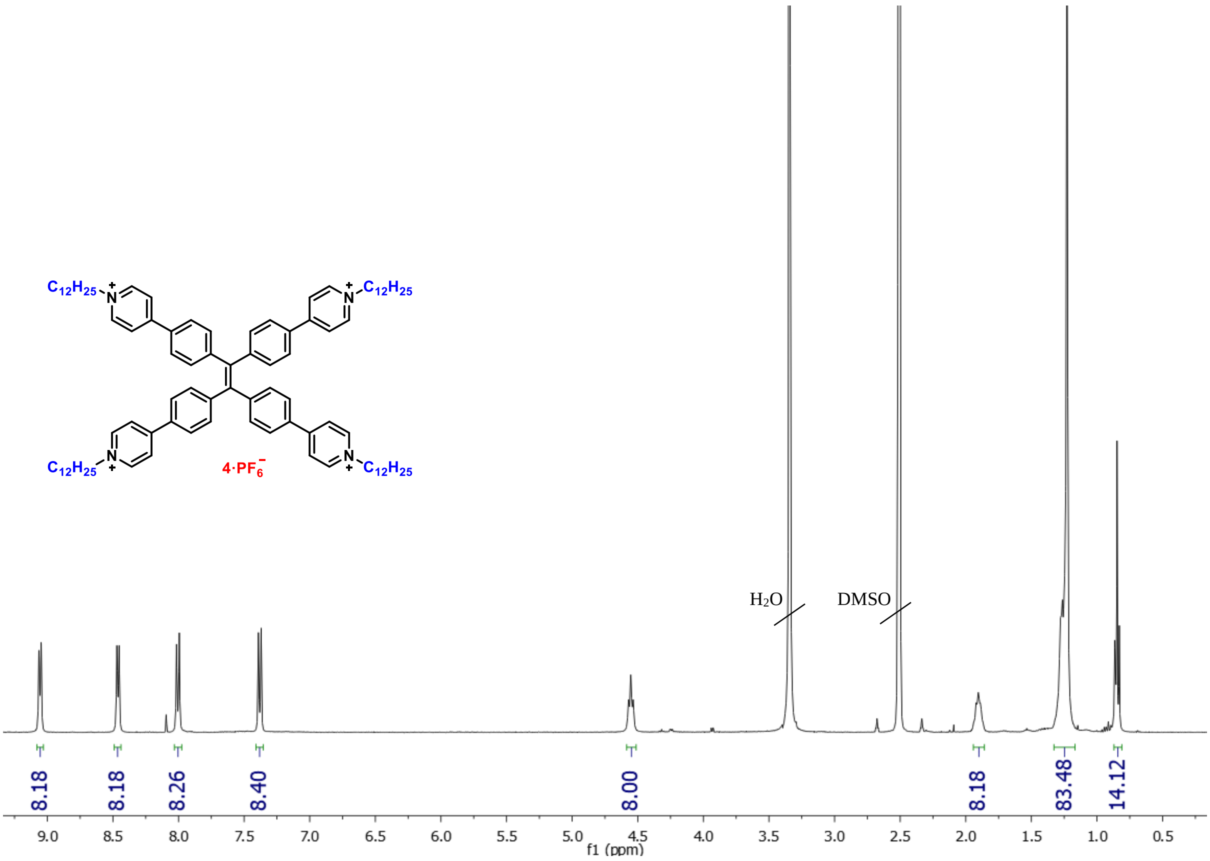

Fig. S56. ^{1}H-NMR (DMSO-d_6, 400 MHz) spectrum of **12.PF$_6$**

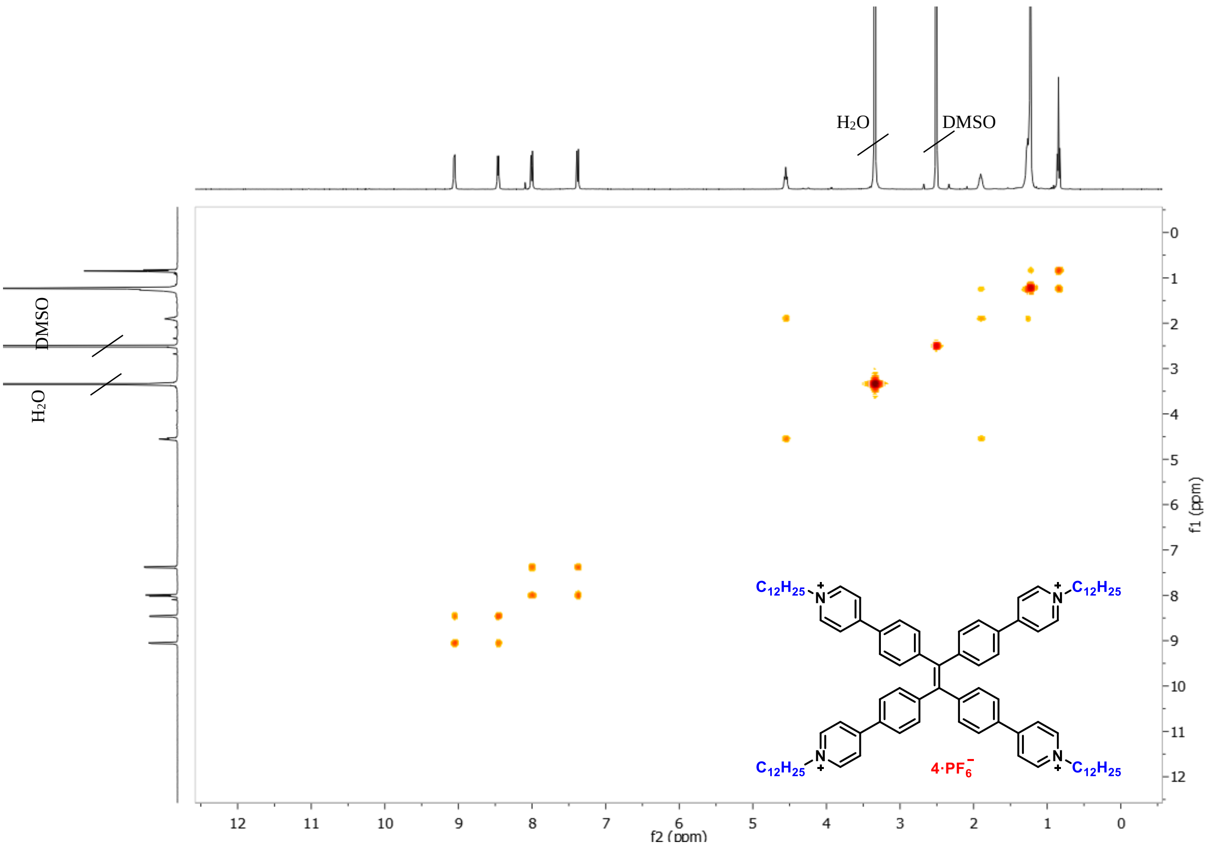

Fig. S57. COSY NMR (DMSO-d6) spectrum of **12.PF$_6$**

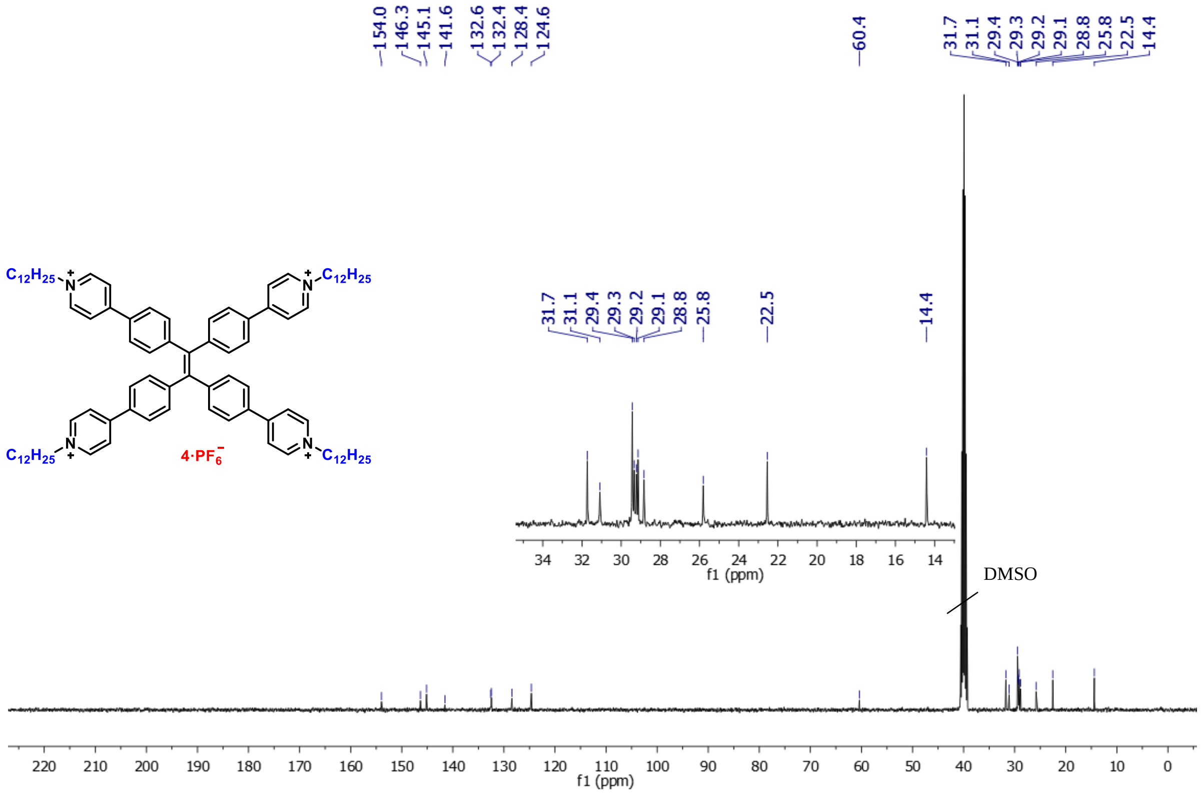

Fig. S58. ^{13}C-NMR (DMSO-d$_6$, 100 MHz) spectrum of **12.PF$_6$**

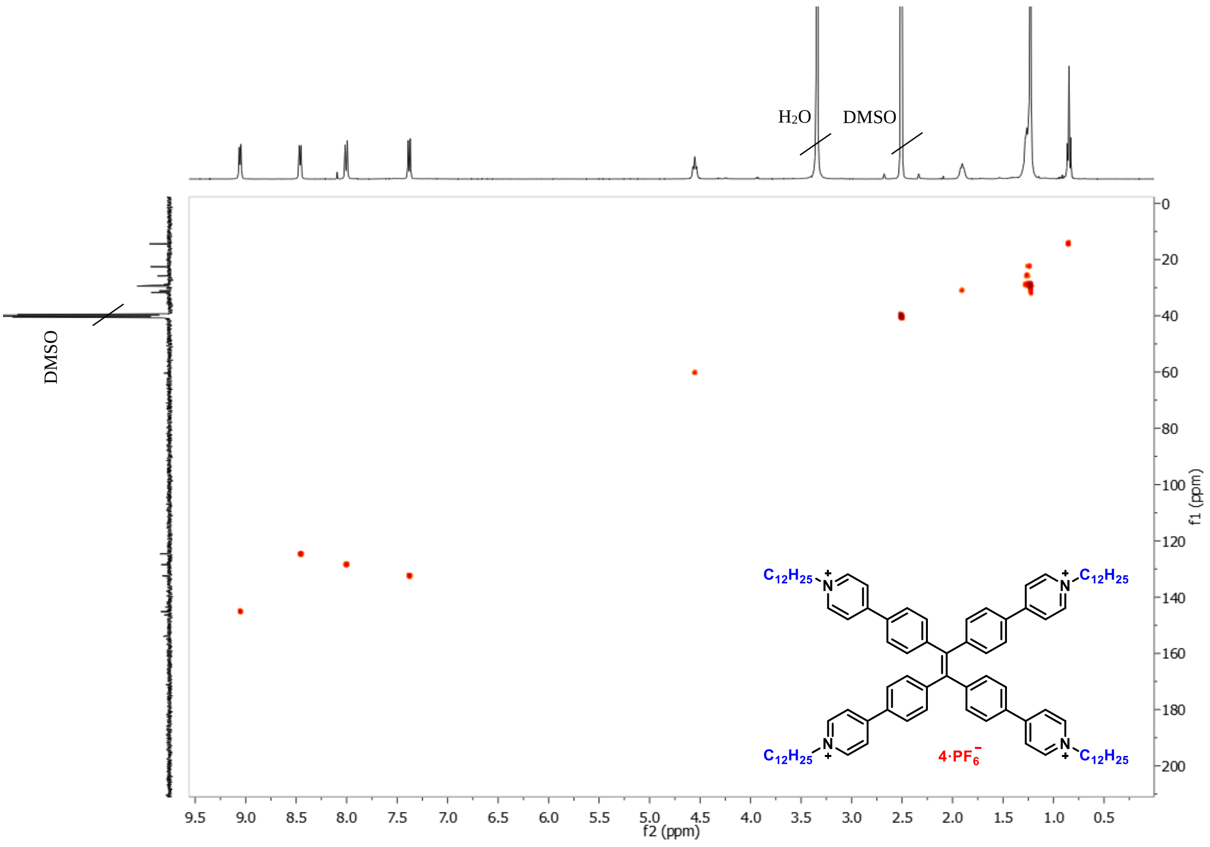

Fig. S59. HSQC (DMSO-d$_6$)spectrum of **12.PF$_6$**

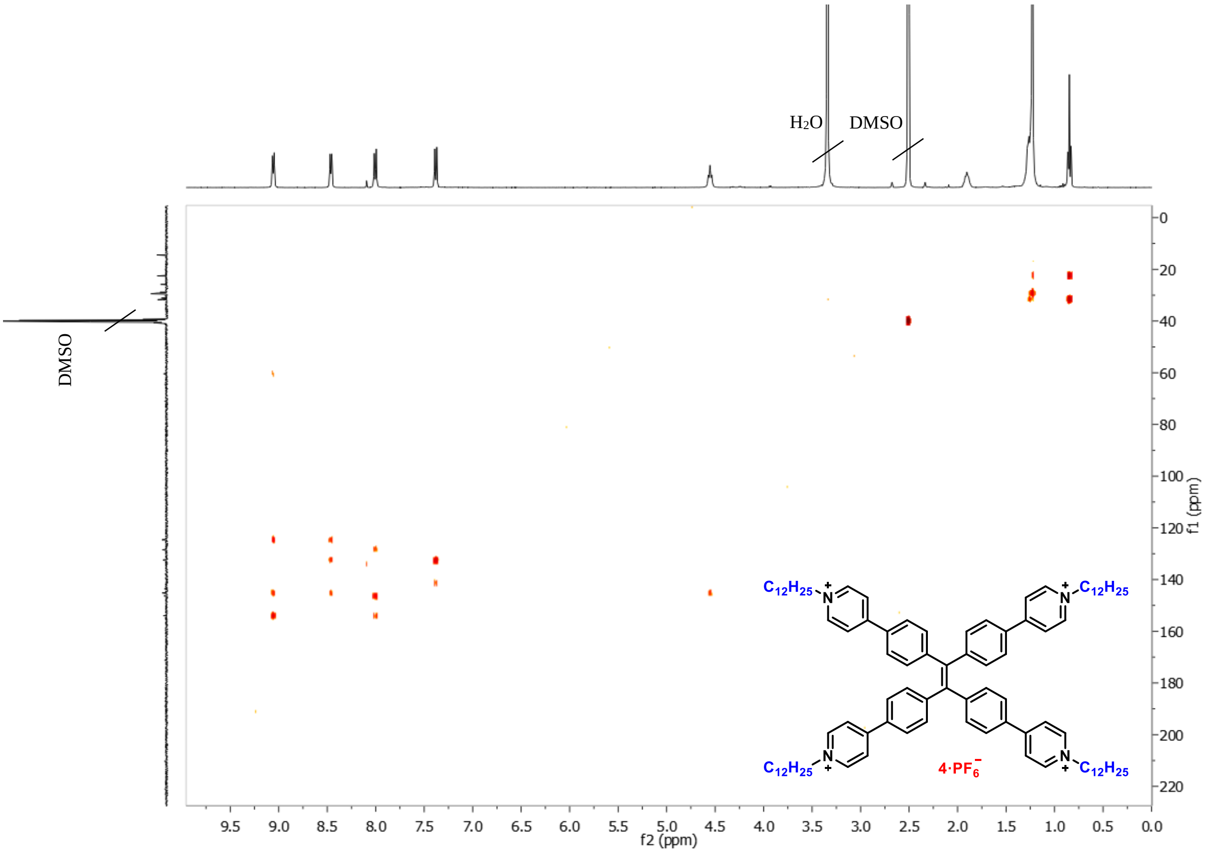

Fig. S60. HMBC (DMSO-d$_6$) spectrum of **12.PF$_6$**

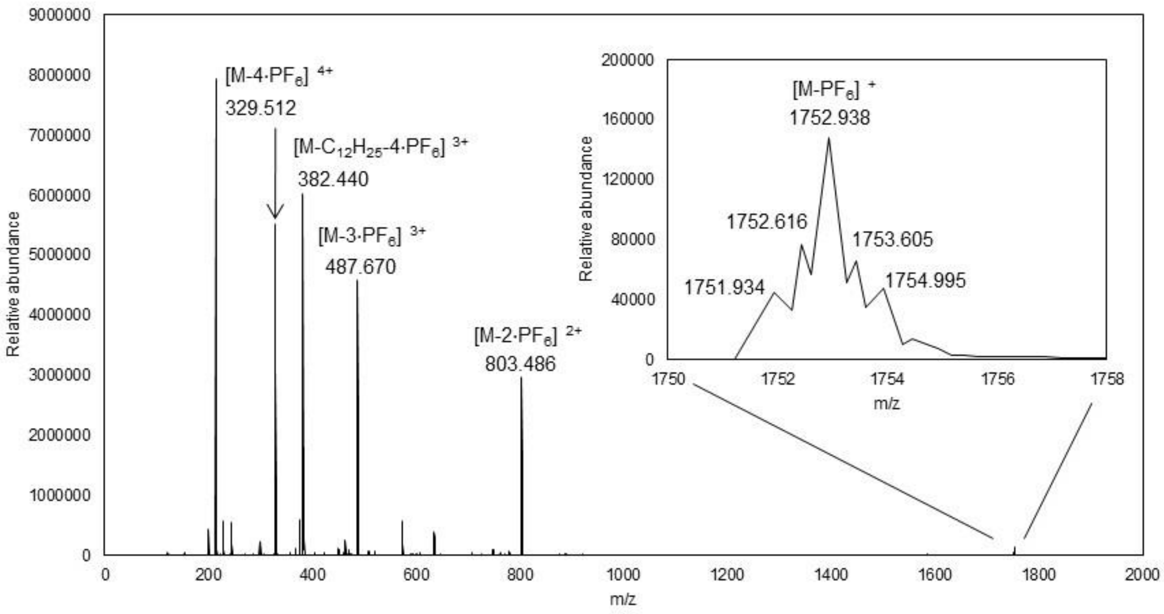

Fig. S61. ESI mass spectrum **$12.PF_6$**

Additional absorbance and emission experiments

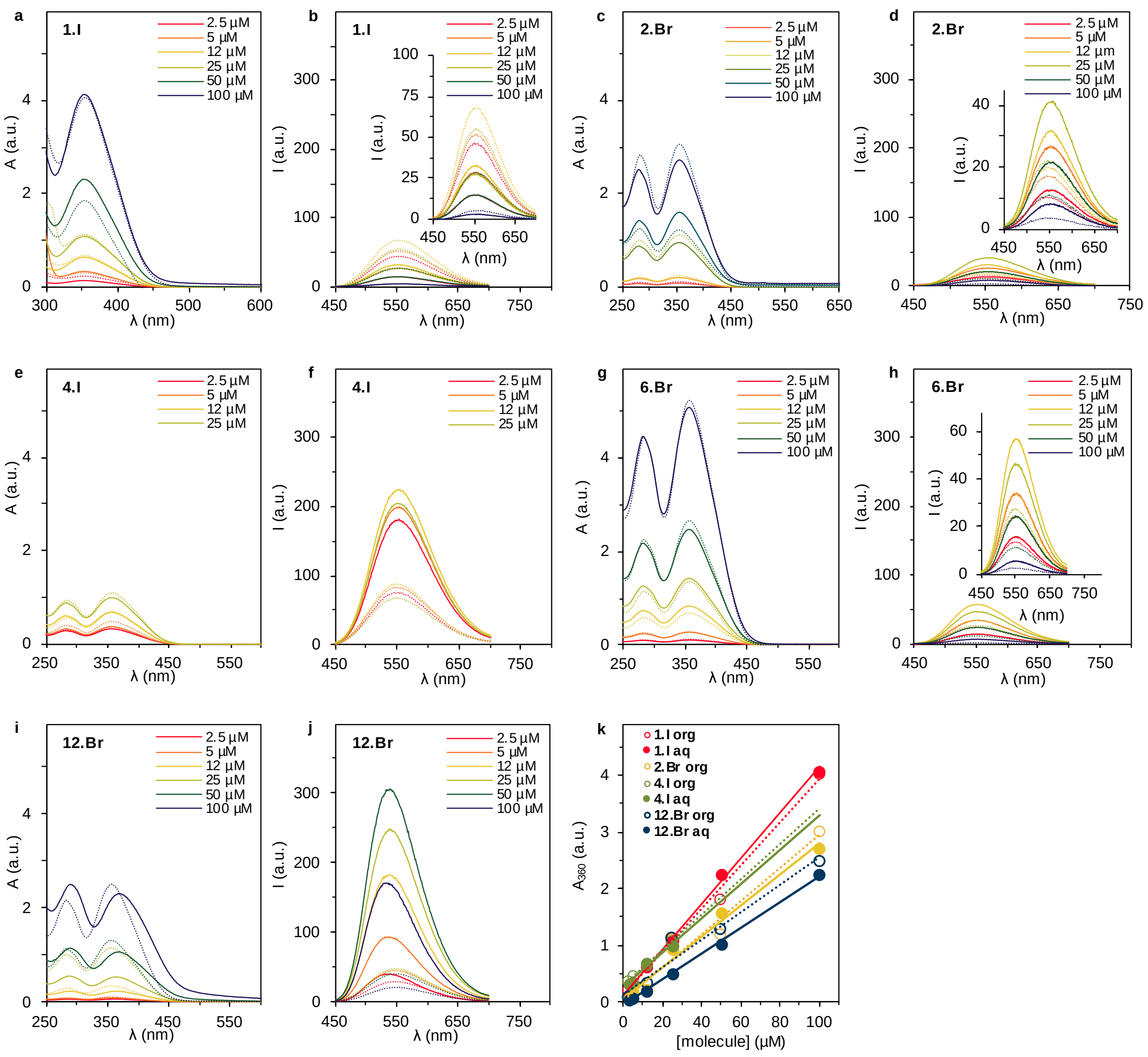

Fig. S62. UV-Vis absorbance and emission spectrum at different concentrations (full and dashed lines for sample in aqueous and organic media, respectively) for (a-b) **1.I** (c-d) **2.Br** (e-f) **4.I** (g-h) **6.Br** and (i-j) **12.Br**. k) A_{360} vs concentration of the halide series in organic solvent (empty circles) and aqueous media (full circles). Trendlines are represented with the same color in dashed and full lines, corresponding to organic solvent and aqueous media, respectively.

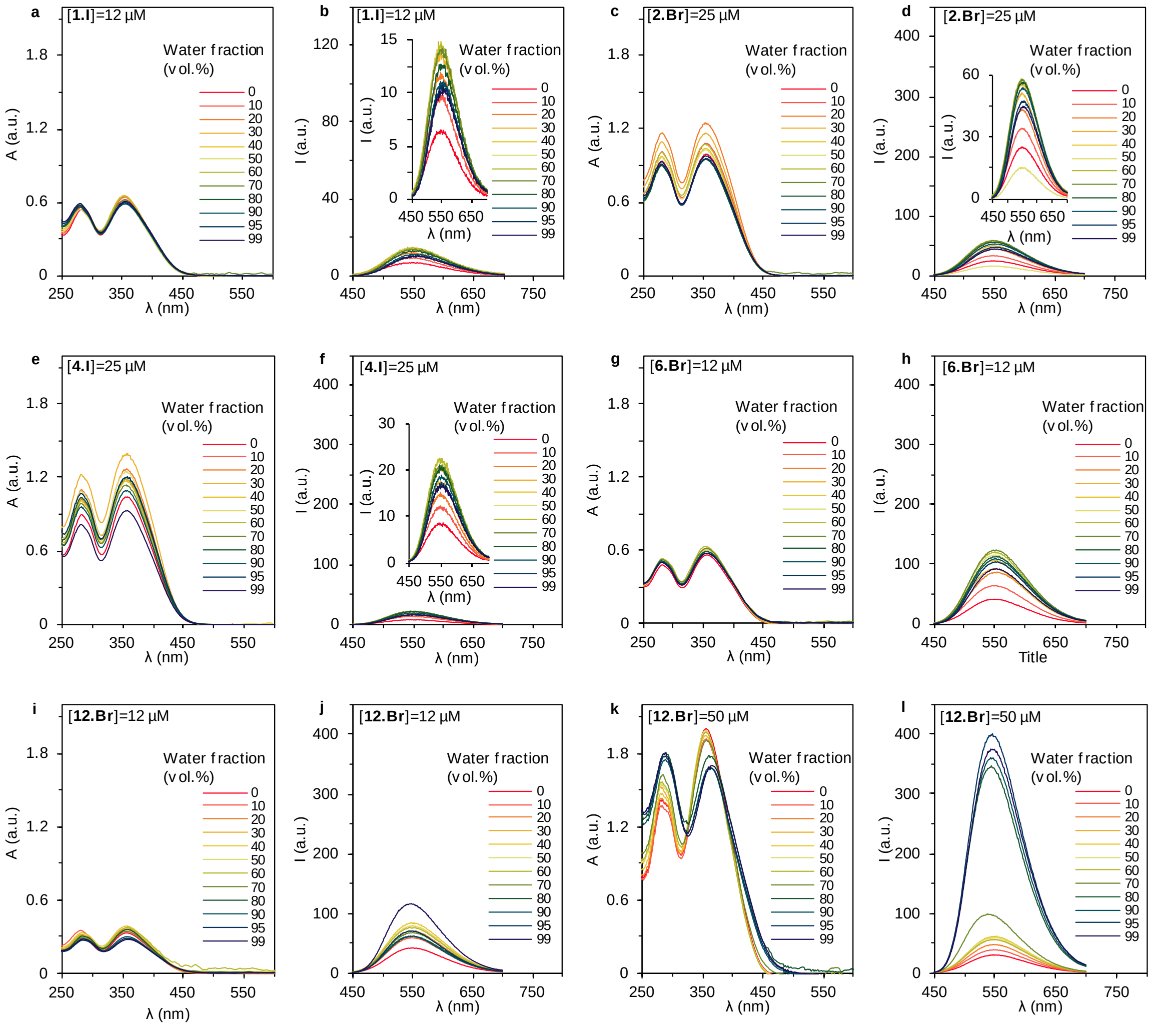

Fig. S63. UV-Vis absorbance and emission spectrum at different water percentages for (a-b) **1.I** at 12 µM (c-d) **2.Br** at 25 µM (e-f) **4.I** at 25 µM (g-h) **6.Br** at 12 µM (i-j) **12.Br** at 12 µM and (k-l) **12.Br** at 50 µM, respectively.

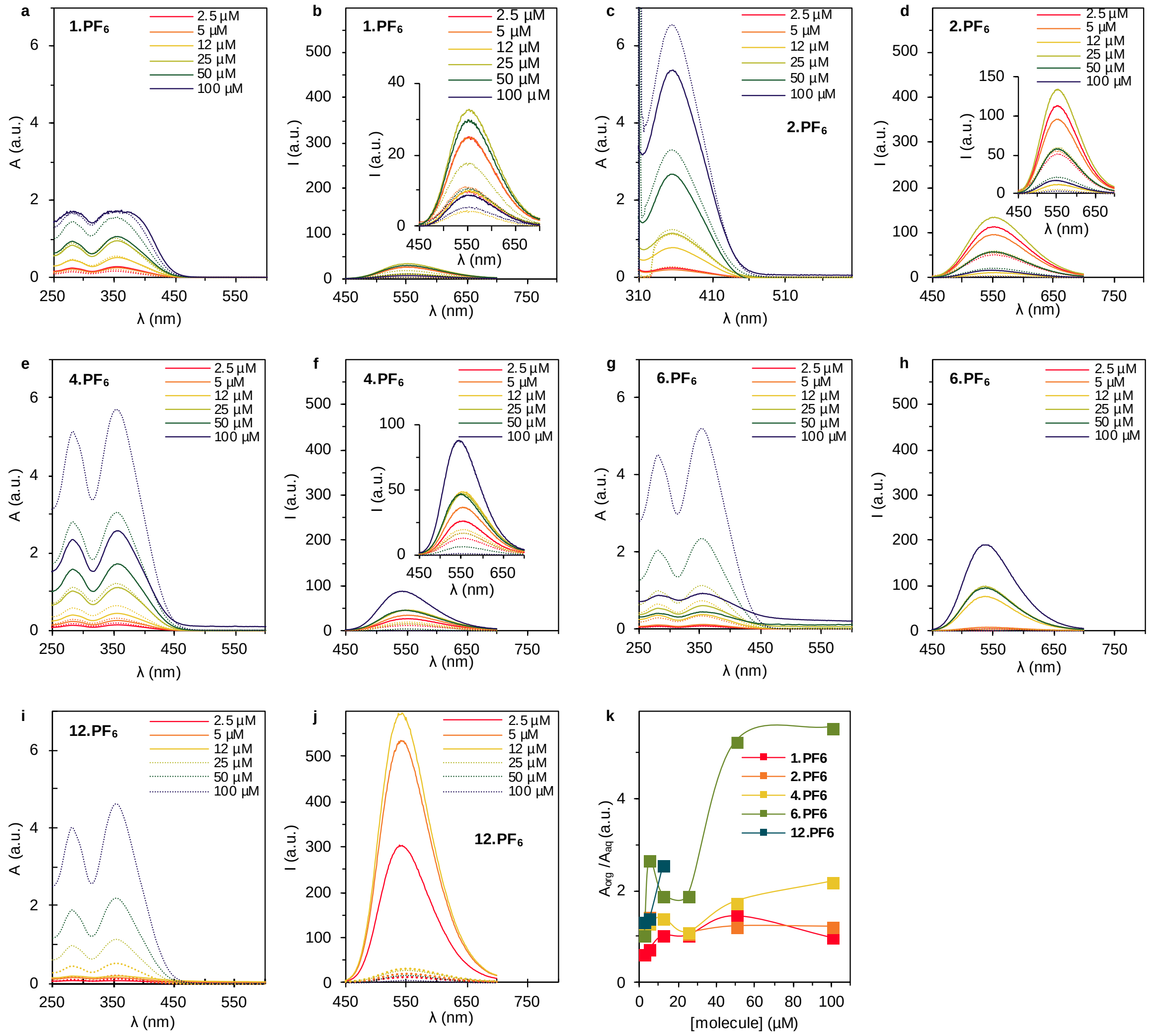

Fig. S64. UV-Vis absorbance and emission spectrum at different concentrations (full and dashed lines for sample in aqueous and organic media, respectively) for (a-b) **1.PF$_6$** (c-d) **2.PF$_6$** (e-f) **4.PF$_6$** (g-h) **6.PF$_6$** at slit 2.5 and (i-j) **12.PF$_6$**, respectively. (k) Ratio A_{org}/ A_{aq} at different concentrations for **1.PF$_6$**, **2.PF$_6$**, **4.PF$_6$**, **6.PF$_6$** and **12.PF$_6$** at 360 nm. For **12.PF$_6$** concentrations higher than 12 µM in aqueous media lead to visible flocculation and particle precipitation.

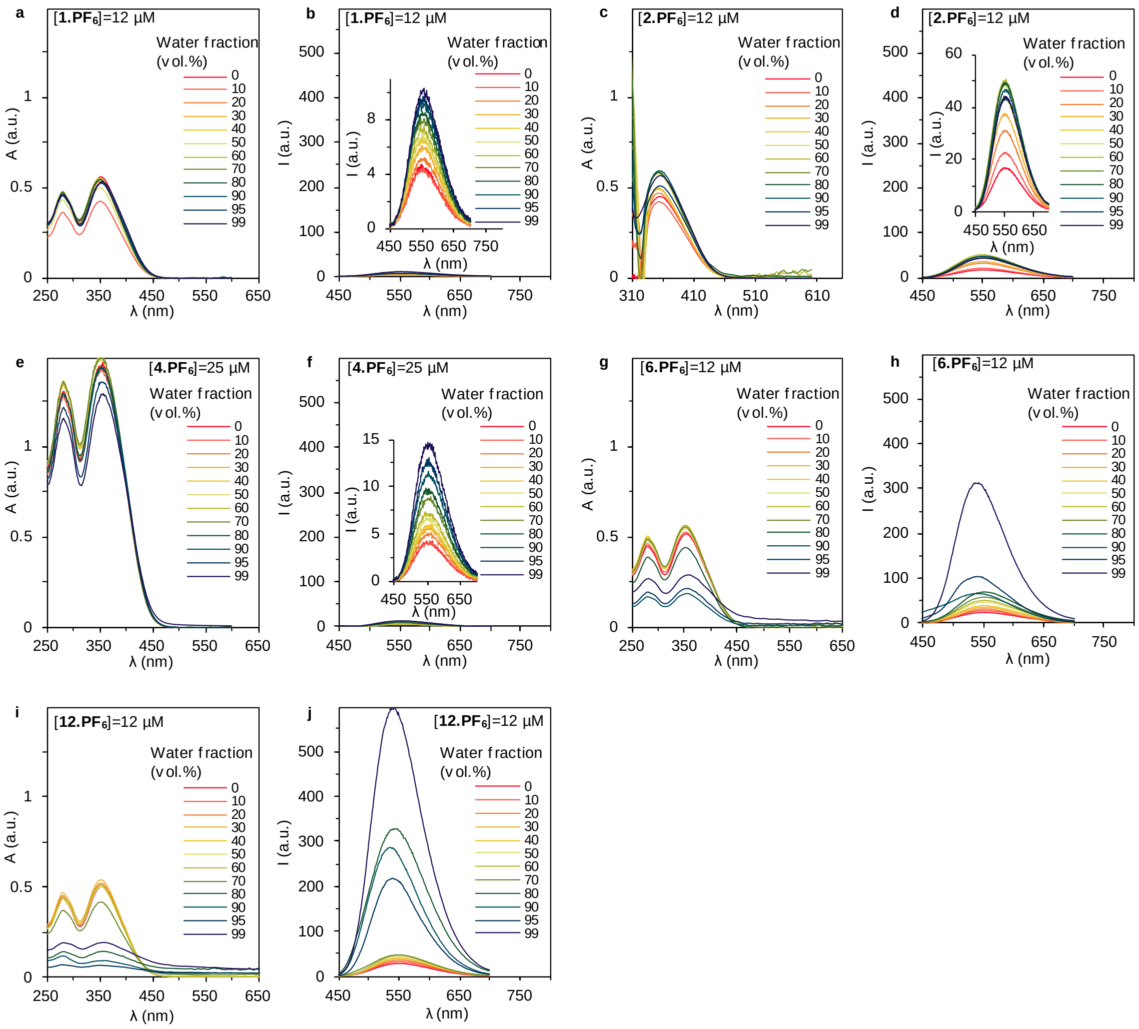

Fig. S65. UV-Vis absorbance and emission spectrum at different water percentages for (a-b) **1.PF₆** at 12 μM (c-d) **2.PF₆** at 12 μM (e-f) **4.PF₆** at 25 μM (g-h) **6.PF₆** at 12 μM and (i-j) 1**2 PF₆** at 12 μM, respectively.

Additional DLS experiments

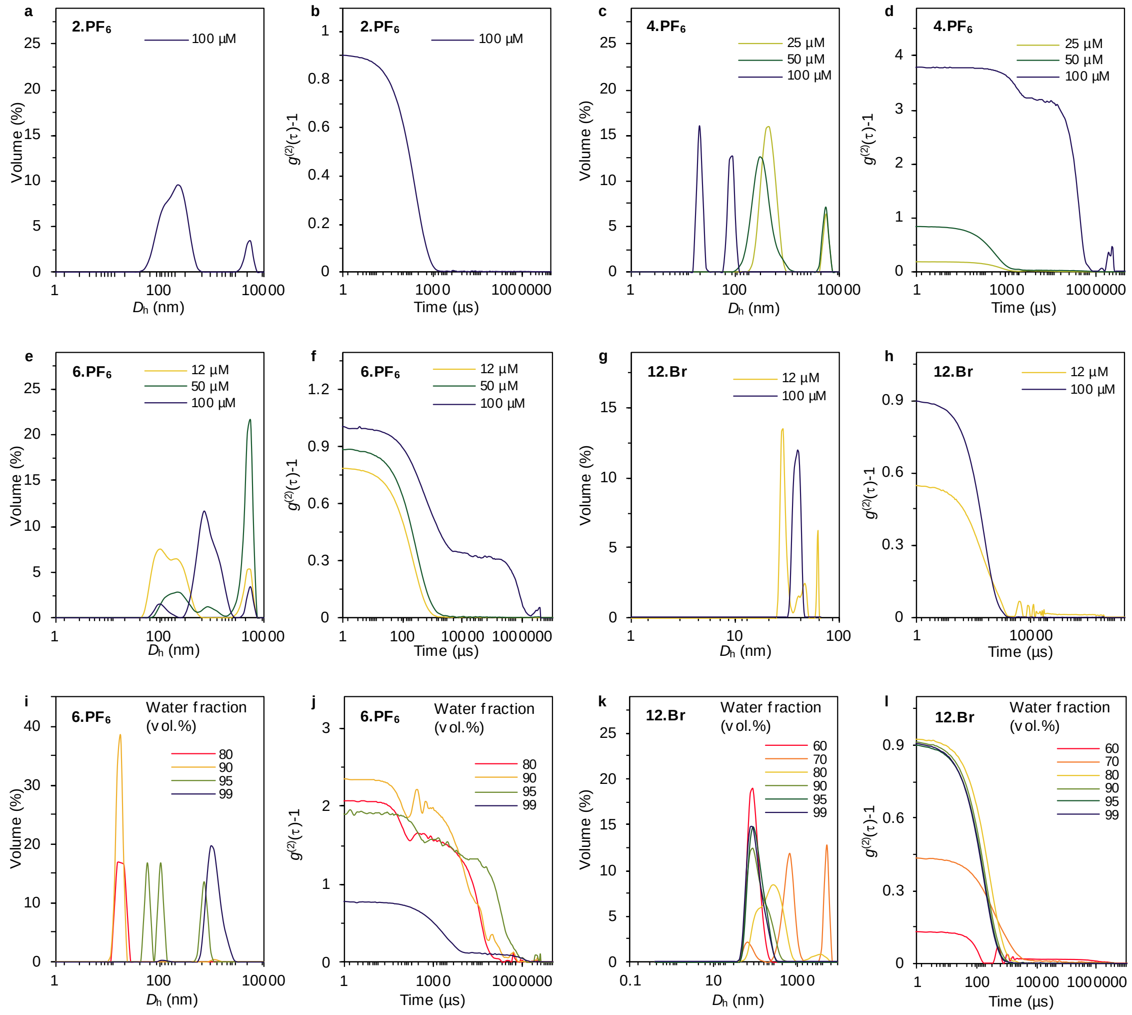

Fig. S66. Particle size distribution and correlation coefficient of the samples in aqueous media measured by DLS at different concentrations (a-b) **2.PF$_6$** (c-d) **4.PF$_6$** (e-f) **6.PF$_6$** (g-h) **12.PF$_6$** and at different water percentages (i-j) titration [**6.PF$_6$**]= 100 µM (k-l) titration [**12.Br**]= 50 µM, respectively.

Square wave voltammetry

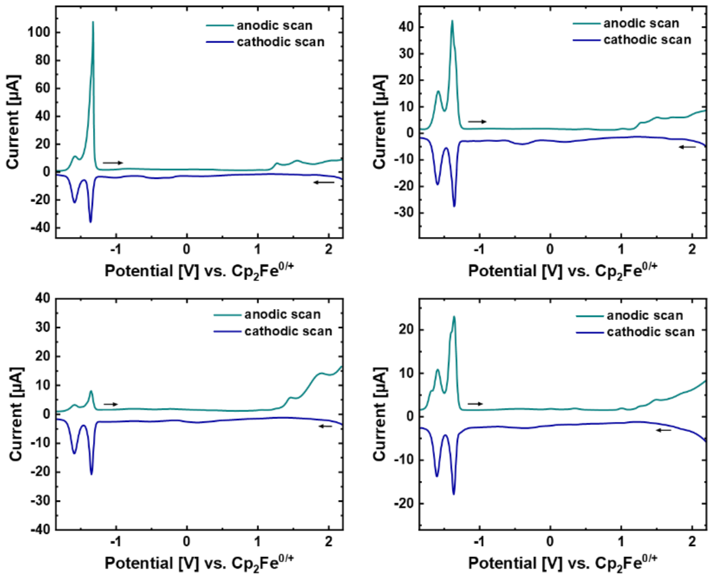

Fig. S67. Square wave voltammograms (step height = 5 mV, frequency = 25 Hz, amplitude = 20 mV) of **1.PF$_6$** (top left), **2.PF$_6$** (top right), **4.PF$_6$** (bottom left), **6.PF$_6$** (bottom right) in MeCN/NBu$_4$PF$_6$ (0.1 m), room temperature; working electrode: platinum, counter electrode: platinum, *quasi* reference electrode: Ag wire.

Normalized absorbance spectra of films in MeCN

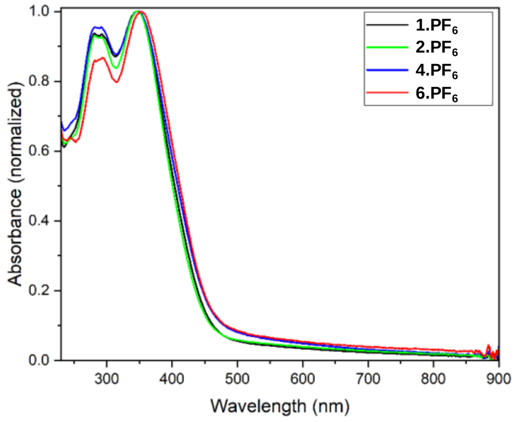

Fig. S68. UV-Vis absorption spectra normalized of **1.PF$_6$**, **2.PF$_6$**, **4.PF$_6$** and **6.PF$_6$** films in MeCN.

Films morphology by AFM

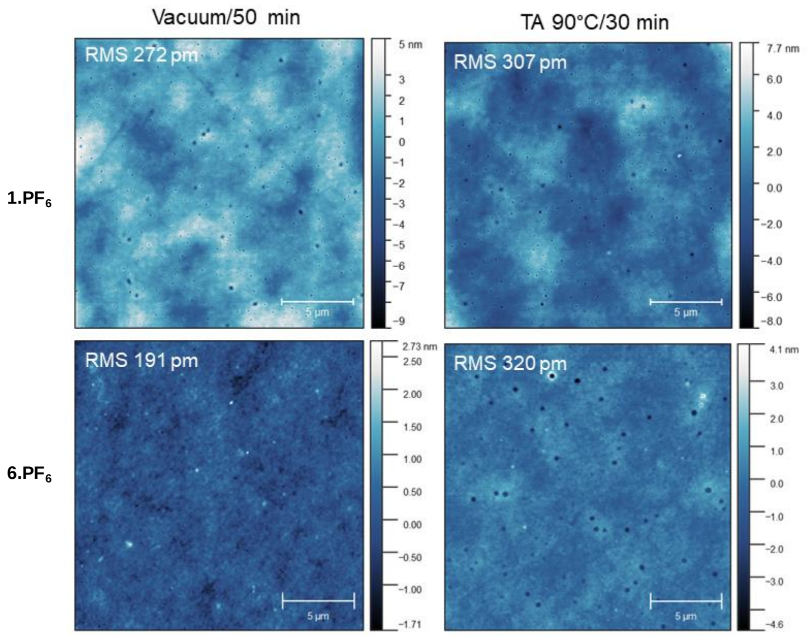

Fig. S69. Films morphology through Atomic Force Microscopy (AFM) of **1.PF$_6$** and **6.PF$_6$** dry under vacuum for 50 min (left) or with thermal annealing of 90 °C for 30 min (right).

Electrochemical impedance spectroscopy (EIS)

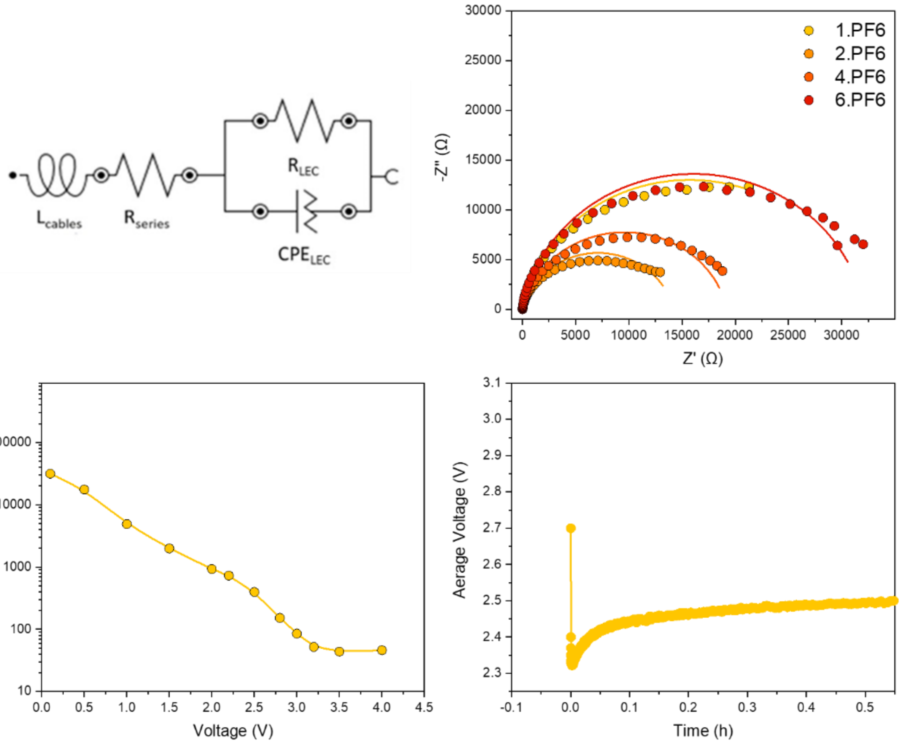

Fig. S70. Equivalent electric circuit scheme used for the fitting of Nyquist plots (top left). Nyquist plots at 0V of fresh **1.PF$_6$**, **2.PF$_6$**, **4.PF$_6$**, **6.PF$_6$** based LECs (top right). Example of R_{LEC} *versus* Voltage

profile from 0V to 5V (bottom left) and example of temporal evolution of the average voltage profile (bottom right).

Bibliography

1 E. Fresta, J. Dosso, J. Cabanillas-González, D. Bonifazi and R. D. Costa, *Adv Funct Mater*, 2020, **30**, 1906830.

2 A. Rananaware, A. N. Abraham, D. D. La, V. Mistry, R. Shukla and S. V. Bhosale, *Aust J Chem*, 2017, **70**, 652–659.
